# xCell: digitally portraying the tissue cellular heterogeneity landscape

**DOI:** 10.1186/s13059-017-1349-1

**Published:** 2017-11-15

**Authors:** Dvir Aran, Zicheng Hu, Atul J. Butte

**Affiliations:** Institute for Computational Health Sciences, University of California, San Francisco, California 94158 USA

## Abstract

Tissues are complex milieus consisting of numerous cell types. Several recent methods have attempted to enumerate cell subsets from transcriptomes. However, the available methods have used limited sources for training and give only a partial portrayal of the full cellular landscape. Here we present xCell, a novel gene signature-based method, and use it to infer 64 immune and stromal cell types. We harmonized 1822 pure human cell type transcriptomes from various sources and employed a curve fitting approach for linear comparison of cell types and introduced a novel spillover compensation technique for separating them. Using extensive in silico analyses and comparison to cytometry immunophenotyping, we show that xCell outperforms other methods. xCell is available at http://xCell.ucsf.edu/.

**Electronic supplementary material:**

The online version of this article (doi:10.1186/s13059-017-1349-1) contains supplementary material, which is available to authorized users.

## Background

In addition to malignant proliferating cells, tumors are also composed of numerous distinct non-cancerous cell types and activation states of those cell types. Together these are termed the tumor microenvironment, which has been in the research spotlight in recent years and is being further explored by novel techniques. The most studied set of non-cancerous cell types are the tumor-infiltrating lymphocytes (TILs). However, TILs are only part of a variety of innate and adaptive immune cells, stromal cells, and many other cell types that are found in the tumor and interact with the malignant cells. This complex and dynamic microenvironment is now recognized to be important both in promoting and inhibiting tumor growth, invasion, and metastasis [1, 2]. Understanding the cellular heterogeneity composing the tumor microenvironment is key for improving existing treatments, the discovery of predictive biomarkers, and development of novel therapeutic strategies.

Traditional approaches for dissecting the cellular heterogeneity in liquid tissues are difficult to apply in solid tumors [3]. Therefore, in the past decade, several methods have been published for digitally dissecting the tumor microenvironment using gene expression profiles [4–7] (reviewed in [8]). Recently, a multitude of studies have been published applying published and novel techniques on publicly available tumor sample resources, such as The Cancer Genome Atlas (TCGA) [6, 9–13]. Two general types of techniques are used: deconvolving the complete cellular composition and assessing enrichments of individual cell types.

At least seven major issues raise concerns that the in silico methods could be prone to errors and cannot reliably portray the cellular heterogeneity of the tumor microenvironment. First, current techniques depend on the expression profiles of purified cell types to identify reference genes and therefore rely heavily on the data source from which the references are inferred and could this be inclined to overfit these data. Second, current methods focus on only a very narrow range of the tumor microenvironment, usually a subset of immune cell types, and thus do not account for the further richness of cell types in the microenvironment, including blood vessels and other different forms of cell subsets [14, 15]. A third problem is the ability of cancer cells to “imitate” other cell types by expressing immune-specific genes, such as a macrophage-like expression pattern in tumors with parainflammation [16]; only a few of the methods take this into account. Fourth, the ability of existing methods to estimate cell abundance has not yet been comprehensively validated in mixed samples. Cytometry is a common method for counting cell types in a mixture and, when performed in combination with gene expression profiling, can allow validation of the estimations. However, in most studies that included cytometry validation, these analyses were performed on only a very limited number of cell types and a limited number of samples [7, 13].

A fifth challenge is that deconvolution approaches are prone to many different biases because of the strict dependencies among all cell types that are inferred. This could highly affect reliability when analyzing tumor samples, which are prone to form non-conventional expression profiles. A sixth problem comes with inferring an increasing number of closely related cell types [10]. Finally, deconvolution analysis heavily relies on the structure of the reference matrix, which limits its application to the resource used to develop the matrix. One such deconvolution approach is CIBESORT, the most comprehensive study to date, which allows the enumeration of 22 immune subsets [7]. Newman et al. [7] performed adequate evaluation across data sources and validated the estimations using cytometry immunophenotyping. However, the shortcomings of deconvolution approaches are apparent in CIBERSORT, which is limited to Affymetrix microarray studies.

On the other hand, gene set enrichment analysis (GSEA) is a simple technique which can be easily applied across data types and can be quickly applied for cancer studies. In GSEA each gene signature is used independently of all other signatures and it is thus protected from the limitations of deconvolution approaches. However, because of this independence, it is many times hard to differentiate between closely related cell types. In addition, gene signature-based methods only provide enrichment scores and thus do not allow comparison across cell types and cannot provide insights into the abundance of cell types in the mixture.

Here, we present xCell, a novel method that integrates the advantages of gene set enrichment with deconvolution approaches. We present a compendium of newly generated gene signatures for 64 cell types, spanning multiple adaptive and innate immunity cells, hematopoietic progenitors, epithelial cells, and extracellular matrix cells derived from thousands of expression profiles. Using in silico mixtures, we transform the enrichment scores to a linear scale, and using a spillover compensation technique we reduce dependencies between closely related cell types. We evaluate these adjusted scores in RNA-seq and microarray data from primary cell type samples from various independent sources. We examine their ability to digitally dissect the tumor microenvironment by in silico analyses, and perform the most comprehensive comparison to date with cytometry immunophenotyping. We compare our inferences with available methods and show that scores from xCell are more reliable for digital dissection of mixed tissues. Finally, we apply our method on TCGA tumor samples to portray a full tumor microenvironment landscape across thousands of samples. We provide these estimations to the community and hope that this resource will allow researchers to gain a better perspective of the complex cellular heterogeneity in tumor tissues.

## Results

### Generating a gene signature compendium of cell types

**Fig. 1:**
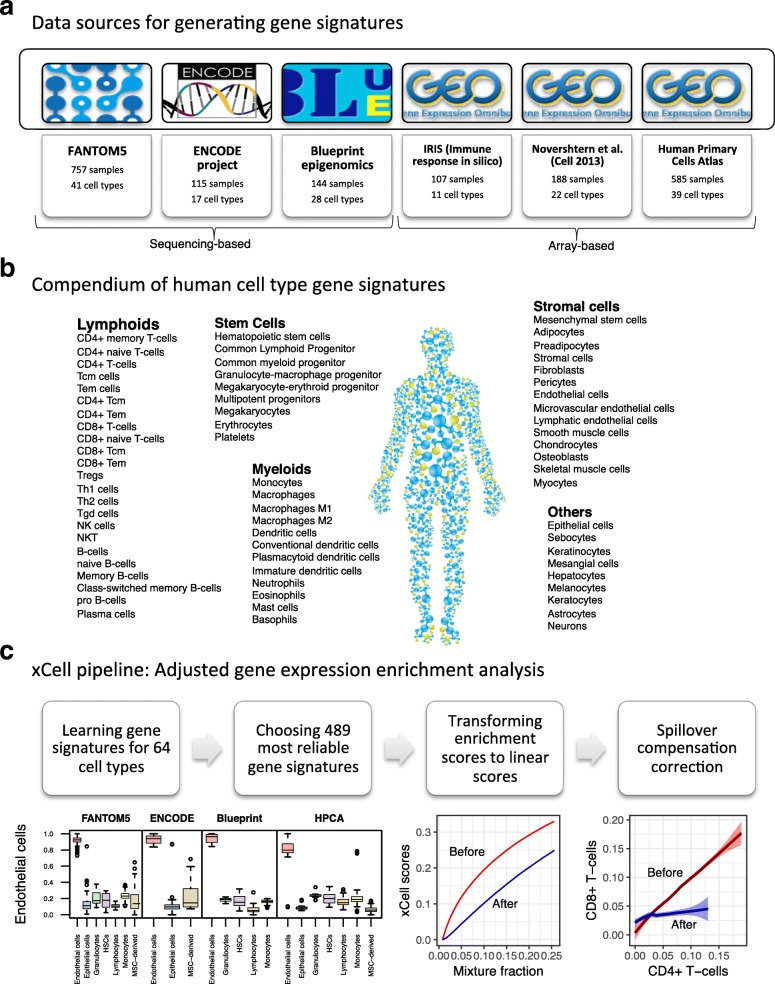
xCell study design. **a** A summary of the data sources used in the study to generate the gene signatures, showing the number of pure cell types and number of samples curated from them. **b** Our compendium of 64 human cell type gene signatures grouped into five cell type families. **c** The xCell pipeline. Using the data sources and based on different thresholds, we derived gene signatures for 64 cell types. Of this collection of 6573 signatures, we chose the 489 most reliable cell types, three for each cell type from each data source where available. The raw score is then the average single-sample GSEA (ssGSEA) score of all signatures corresponding to the cell type. Using simulations of gene expression for each cell type, we derived a function to transform the non-linear association between the scores to a linear scale. Using the simulations we also derive the dependencies between cell type scores and apply a spillover compensation method to adjust the scores

To generate our compendium of gene signatures for cell types, we collected gene expression profiles from six sources: the FANTOM5 project, from which we annotated 719 samples from 39 cell types analyzed by the Cap Analysis Gene Expression (CAGE) technique [17]; the ENCODE project, from which we annotated 115 samples from 17 cell types analyzed by RNA-seq [18]; the Blueprint project, from which we annotated 144 samples from 28 cell types analyzed by RNA-seq [19]; the IRIS project, from which we annotated 95 samples from 13 cell types analyzed by Affymetrix microarrays [20]; the Novershtern et al. [21] study, from which we annotated 180 samples from 24 cell types analyzed by Affymetrix microarrays; and the Human Primary Cells Atlas (HPCA), a collection of Affymetrix microarrays composed of many different Gene Expression Omnibus (GEO) datasets, from which we annotated 569 samples from 41 cell types [22] (Fig. 1a). Altogether we collected and curated gene expression profiles from 1822 samples of pure cell types, annotated to 64 distinct cell types and cell subsets (Fig. 1b; Additional file 1). Of those, 54 cell types were found in at least two of these data sources. For cell types with five or more samples in a data source, we left one sample out for testing. All together, 97 samples were left out, and all of the model training described below was performed on the remaining 1725 samples.

Our strategy for selecting reliable cell type gene signatures is shown in Fig. 1c (see Additional file 2: Figure S1 and “Methods” for a full description and technical details). For each data source independently we identified genes that are overexpressed in one cell type compared to all other cell types. We applied different thresholds for choosing sets of genes to represent the cell type gene signatures; hence, from each source, we generated dozens of signatures per cell type. This scheme yielded 6573 gene signatures corresponding to 64 cell types. Importantly, since our primary aim is to develop a tool for studying the cellular heterogeneity in the tumor microenvironment, we applied a methodology we previously developed [16] to filter out genes that tend to be overexpressed in a set of 634 carcinoma cell lines from the Cancer Cell Line Encyclopedia (CCLE) [23].

Next, we used single-sample GSEA (ssGSEA) to score each sample based on all signatures. ssGSEA is a well-known method for determining a single, aggregate score of the enrichment of a set of genes in the top of a ranked gene expression profile [24]. To choose the most reliable signatures we tested their performance in identifying the corresponding cell type in each of the data sources. To prevent overfitting, each signature learned from one data source was tested in other sources, but not in the data source from which it was originally inferred. To reduce biases resulting from a small number of genes and from the analysis of different platforms, instead of one signature per cell type, the top three ranked signatures from each data source were chosen. Altogether we generated 489 gene signatures corresponding to 64 cell types spanning multiple adaptive and innate immunity cells, hematopoietic progenitors, epithelial cells, and extracellular matrix cells (Additional file 3). Observing the scores in the 97 test primary cell type samples affirmed their ability to identify the corresponding cell type compared to other cell types across data sources (Additional file 2: Figure S2). We defined the raw enrichment score per cell type to be the average ssGSEA score from all the cell types’ corresponding signatures.

### Spillover compensation between closely related cell types

Our primary objective is to accurately identify enrichment of cell types in mixtures. To imitate such admixtures, we performed an array of simulations of gene expression combinations for different cell types to assess the accuracy and sensitivity of our gene signatures. We generated such in silico expression profiles using different data sources and different sets of cell types in mixtures and by choosing randomly one sample per cell type from all available samples in the data source. The simulations revealed that our raw scores reliably predict even small changes in the proportions of cell types, distinguish between most cell types, and are reliable in different transcriptomic analysis platforms (Additional file 2: Figure S3). However, the simulations also revealed that raw scores of RNA-seq samples are not linearly associated with the abundance and that they do not allow comparisons across cell types (Additional file 2: Figure S4). Thus, using the training samples we generated synthetic expression profiles by mixing the cell type of interest with other, non-related cell types. We then fit a formula that transforms the raw scores to cell type abundances. We found that the transformed scores showed resemblance to the known fractions of the cell types in simulations, thus enabling comparison of scores across cell types, and not just across samples (Additional file 2: Figure S5).

The simulations also revealed another limitation of the raw scores: closely related cell types tend to have correlating scores (Additional file 2: Figure S5). That is, scores may show enrichment for a cell type due to a “spillover effect” between closely related cell types. This problem mimics the spillover problem in flow cytometry, in which fluorescent signals correlate with each other due to spectrum overlaps. Inspired by the compensation method used in flow cytometry studies [25], we leveraged our simulations to generate a spillover matrix that allows correcting for correlations between cell types. To better compensate for low abundances in mixtures, we created a simulated dataset where each sample contains 25% of the cell type of interest with the rest from a non-related cell type and produced a spillover matrix, a representation of the dependencies of scores between different cell types.

**Fig. 2:**
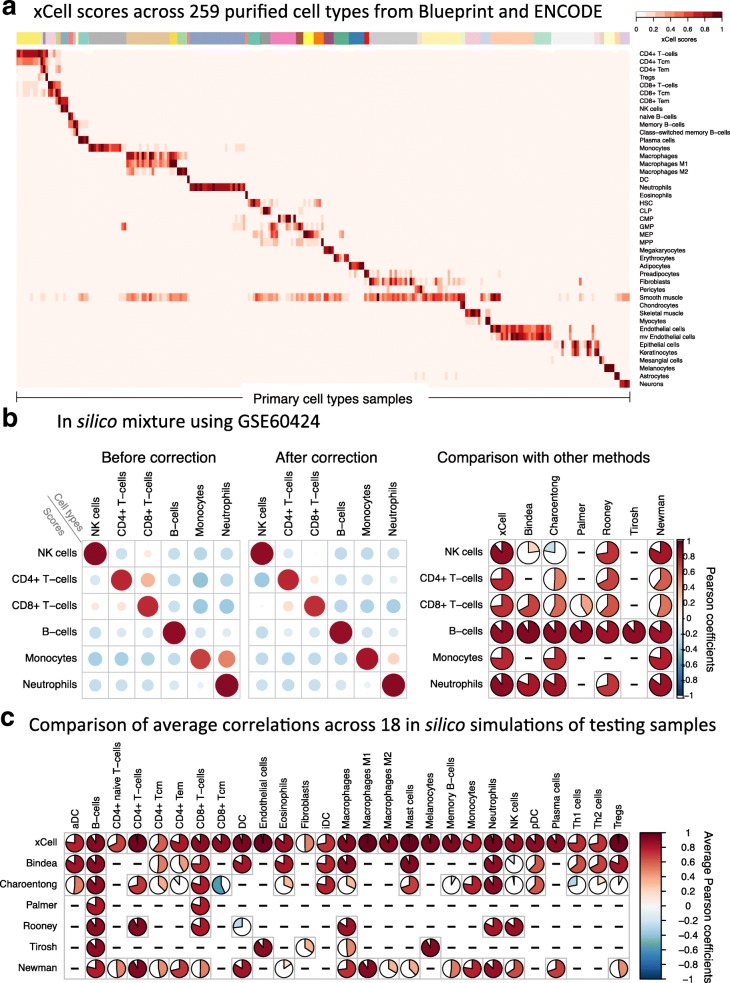
Evaluation of the performance of xCell using simulated mixtures. **a** An overview of adjusted scores for 43 cell types in 259 purified cell type samples from the Blueprint and ENCODE data sources (other data sources are in Additional file 2: Figure S4). Most signatures clearly distinguish the corresponding cell type from all other cell types. **b** A simulation analysis using GSE60424 as the data source [26], which was not used in the development of xCell. This data source contains 114 RNA-seq samples from six major immune cell types. *Left*: Pearson correlation coefficients using our method before spillover adjustment and after the adjustment. Dependencies between CD4+ T cells, CD8+ T cells, and NK cells were greatly reduced; spillover from monocytes to neutrophils was also removed. *Right*: Comparison of the correlation coefficients across the different methods. The first column corresponds to xCell’s predictions of the underlying abundances of the cell types in the simulations (both color and pie chart correspond to average Pearson coefficients). Bindea, Charoentong, Palmer, Rooney, and Tirosh represent sets of signatures for cell types from the corresponding manuscripts. Newman refers to the inferences produced using CIBERSORT on the simulations. xCell outperformed the other methods in 17 of 18 comparisons. **c** Comparison of the correlation coefficients across the different methods based on 18 simulations generated using the left-out testing samples. Here rows correspond to methods and columns show the average Pearson coefficient for the corresponding cell type across the simulations. Independent simulations are available in Additional file 2: Figure S6. xCell outperformed the other methods in 64 of 67 comparisons

Applying the spillover correction procedure on the pure cell types (Fig. 2a) and simulated expression profiles (Fig. 2b, c; Additional file 2: Figures S5 and S6) showed that this method was able to successfully reduce associations between closely related cell types. For example, we generated simulated mixtures using an independent data source of multiple cell types that was not used for the development of the method (GSE60424) [26], and used our method to infer the underlying abundances. We observed decent performance in recapitulating the cell type distributions. However, before correcting for spillovers, there were false associations between CD4+ and CD8+ T cells, as well as between monocytes and neutrophils. The spillover correction was able to reduce these associations significantly without harming the correlations on the diagonal (Fig. 2b). In addition, we generated simulated mixtures using the training samples (Additional file 2: Figure S5) and the test samples (Additional file 2: Figure S6). In the 18 simulated mixtures using the test samples, we observed an overall average decrease of 17.1% in significant correlations off the diagonal (Fig. 2c; Additional file 2: Figure S5). Unexpectedly, following the spillover compensation we observed slightly improved associations on the diagonal between the scores and the underlying abundances (1.4% average improvement).

Finally, many of the cell types we estimate are not expected to be in a given mixture; however, the pipeline we described will often produce non-zero scores. In the 18 test simulated mixtures, 56.4% of the scores for cell types that are not part of the mixture were non-negligible (> 0.001). To overcome this inadequacy, we introduce a statistical significance test of whether a produced enrichment score is not random—whether the cell type of interest is in the mixture. Using the reference training data sets, for each cell type we generated random mixtures of all cell types except the corresponding cell type, and calculated the cell type-adjusted scores. We then fit a beta distribution for each of the cell types and used these distributions to calculate the probability that the score of the corresponding cell type is present in the mixture by random (Additional file 2: Figure S7). Applying this procedure to the test simulated mixtures enabled detection of about half of the non-expected non-negligible scores as non-significant (46.9% change—from 56.4% non-negligible scores to 28.8% with *p* value > 0.2), while detecting as non-significant only 15.3% of non-negligible scores for cell types used for generating the mixture (from 88.6% non-negligible scores to 75.1%) (Additional file 4).

This pipeline for generating adjusted cell type enrichment scores from gene expression profiles, which we named xCell, is available as an R package and a simple web tool (http://xCell.ucsf.edu/).

### Validation of enrichment scores in simulated expression profiles

We next compared the ability of xCell scores to infer the underlying cell type enrichments in simulated mixtures with a set of 53 previously published signatures corresponding to 26 cell types [6, 12, 27, 28] (Additional file 5). Our analyses showed that xCell outperformed the previously published signatures in recapitulating the underlying abundances, in mixtures generated using the training samples (Additional file 2: Figure S5) and the test samples (Additional file 2: Figure S6) and an independent data source (GSE60424 [26]) (Fig. 2b), in the vast majority of the comparable cell types (51 of 53 comparisons of mixtures generated using training samples, 46 of 49 using test samples, and 17 of 18 using GSE60424) (Fig. 2c). xCell showed overall better performance with all data sources used, proving its versatility across platforms. Importantly, our compensation technique was able to completely remove associations between cell types, while previously published signatures showed considerate dependencies between closely related cell types, such as between CD8+ T cells and NK cells (Additional file 2: Figure S8).

In addition, we also compared xCell’s performance on test mixtures with that of CIBERSORT, a prominent deconvolution-based method [7]. Unlike signature-based methods, which output independent enrichment scores per cell type, the output from deconvolution-based methods is the inferred proportions of the cell types in the mixture. Similar to the performance comparisons using signatures, xCell also outperformed CIBERSORT in all comparable cell types, across all data sources (Fig. 2b, c; Additional file 2: Figures S5 and S6).

### Validation of enrichment scores with cytometry immunoprofiling

**Fig. 3:**
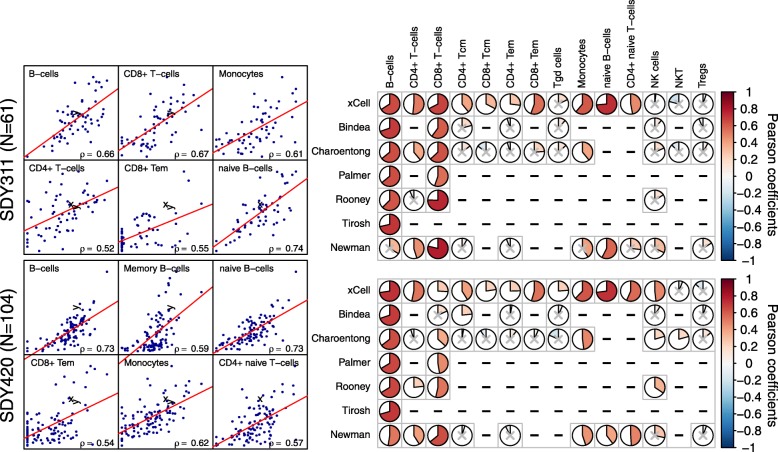
Comparison of digital dissection methods with flow cytometry counts. *Left*: Scatter plots of CyTOF fractions in PBMCs vs. cell type scores from whole blood of 61 samples from SDY311 (*top*) and 104 samples from SDY420 (*bottom*). Only the top correlating cell types in each study are shown. *Right*: Correlation coefficients produced by our method compared to other methods. Only cell types with abundance of at least 1% on average, as measured by CyTOF, are shown. Non-significant correlations (*p* value < 0.05) are marked with a gray “*x*”

In addition to the simulated mixture analysis, we compared our estimates for cell type enrichments from gene expression profiles with mass spectrometry (CyTOF) immunophenotyping. We utilized independent publicly available studies in which a total of 165 individuals were studied for both gene expression from whole blood and FACS across 18 cell subsets from peripheral blood mononuclear cells (PBMCs; available from ImmPort, studies SDY311 and SDY420) [29]. We calculated xCell scores for each of the signatures using the studies’ expression profiles and correlated the scores with the FACS fractions of the cell subsets. Of the 14 cell types with at least 1% abundance, xCell was able to significantly recover 10 and 12 cell subsets in SDY311 and SDY420, respectively (Pearson correlation between calculated and actual cell counts *p* value < 0.05; Fig. 3). Comparing the performance of xCell to previously published signatures and CIBERSORT revealed that no other method was able to recover cell types that our method was not able to recover in both data sets (Fig. 3). In general, previous methods were able to recover signal only from major cell types, including B cells, CD4+ and CD8+ T cells, and monocytes, suggesting that their performance was not reliable in more specialized cell subsets. While our method also struggled in these cell subsets, it still showed significant correlations with most of the cell subsets, including effector memory CD8+ T cells, naïve CD4+ T cells, and naïve B cells. In addition, xCell was more reliable in CD4+ T cells and monocytes and equally reliable in B cells (Fig. 3). In CD8+ T cells xCell was outperformed by methods depending solely on CD8A expression, which may not serve as a reliable biomarker in cancer settings (Additional file 2: Figure S9).

Despite the generally improved ability of xCell to estimate cell populations, we do note that in some cases the correlations we observed were relatively low, emphasizing the difficulty of estimating cell subsets in mixed samples, and the need for cautious examination and further validation of findings.

### Cell type enrichment in tumor samples

**Fig. 4:**
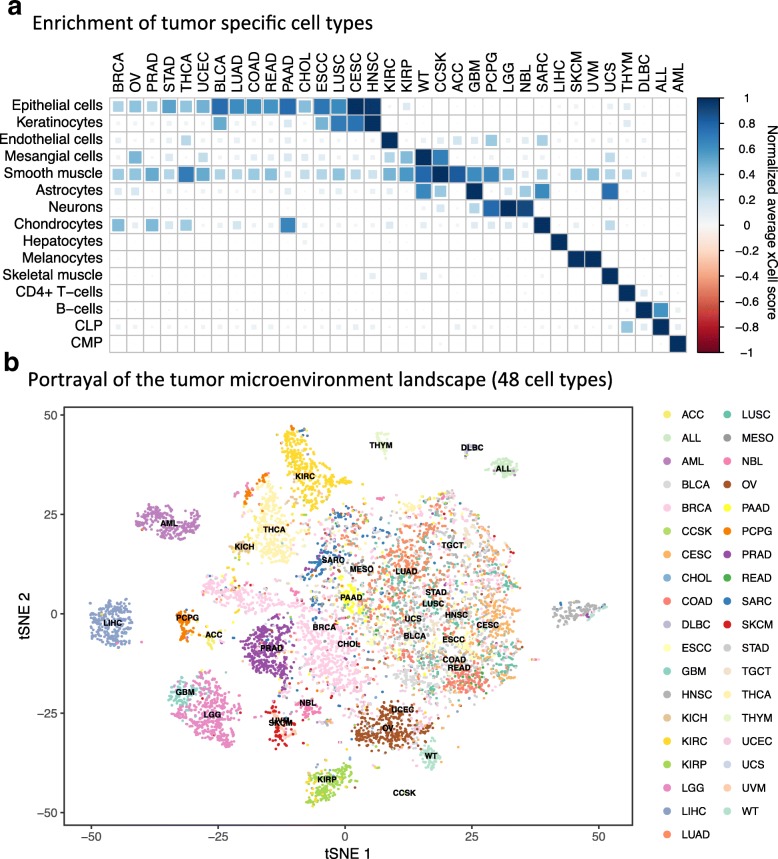
Cell type enrichment analysis in tumors. **a** Average scores for nine cell types across 24 cancer types from TCGA (The Cancger Genome Atlas). Scores were normalized across rows. Signatures were chosen such that they are the cell of origin of a cancer type or the most significant signature of the cancer type compared to all others. **b** t-SNE (t-Distributed Stochastic Neighbor Embedding) plot of 8875 primary cancer samples from TCGA (The Cancger Genome Atlas) and TARGET colored by cancer type. The t-SNE plot was generated using the enrichment scores of 48 non-epithelial, non-stem cell, and non-cell type-specific scores. Many of the cancer types create distinct clusters, emphasizing the important role of the tumor microenvironment in characterizing tumors

We next applied our methodology to 9947 primary tumor samples across 37 cancer types from the TCGA and TARGET projects [30] (Additional file 2: Figure S10). Average scores of cell types in each cancer type affirmed prior knowledge of expected enriched cell types, validating the power of our method for identifying the cell type of origin of different cancer types. For example, epithelial cells were enriched in carcinomas, keratinocytes in squamous cell carcinomas, mesangial cells in kidney cancers, chondrocytes in sarcoma, neurons in brain tumors, hepatocytes in hepatocellular carcinoma, melanocytes in melanomas, B cells in B-cell lymphoma, T cells in thymoma, myeloid cells in acute myeloid leukemia, and lymphocytes in acute lymphocytic leukemia (Fig. 4a). While these results are expected, it is reassuring that xCell can be applied to human cancers.

Most of the cell types we infer are part of the complex cellular heterogeneity of the tumor microenvironment. We hypothesized that an additive combination of all cell types’ scores would be negatively correlated with tumor purity. Thus, we generated a microenvironment score as the sum of all immune and stromal cell types. We then correlated this microenvironment score with our previously generated purity estimations, which are based on copy number variations, gene expression, DNA methylation, and H&E slides [31]. Our analysis showed highly significant negative correlations in all cancer types, suggesting this score as a novel measurement for tumor microenvironment abundance (Additional file 2: Figure S11).

Finally, to provide insight into the potential of xCell to portray the tumor microenvironment, we plotted all tumor samples based on their cell type scores. Using different sets of cell type inferences, we applied the t-Distributed Stochastic Neighbor Embedding (t-SNE) dimensionality reduction technique [32] (Additional file 2: Figure S12). Interestingly, the analysis revealed that unique microenvironment compositions characterize different cancer indications. For example, prostate cancers form a unique cluster based on their immune cell type composition, while head and neck tumors are distinguished by their stromal composition. Remarkably, only when performing the analysis with all immune and stromal cell types did clear clusters form distinguishing between most of the cancer types (Fig. 4b), demonstrating the unique composition of the tumor microenvironment, which differs between cancer types. This notion emphasizes the importance of portraying the full cellular heterogeneity of the tumor microenvironment for the study of cancer. To this end, we calculated the enrichment scores for 64 cell types across the TCGA spectrum, and provide these data with the hope that they will serve the research community as a resource to further explore novel associations of cell type enrichment in human tumors (Additional file 6).

## Discussion

Recently, many studies have shown different methodologies for the digital dissection of cancer samples [3, 6, 9–13]. These studies have provided novel insights into cancer research and related to therapy efficacy. However, it is important to remember that the methods that have been applied for portraying the tumor microenvironment have only attained limited validation, and it is unclear how reliable their estimations are. In this study, we took a step back and focused on generating cell type gene scores that could reliably estimate enrichment of cell types. Our method, which is gene signature-based, is more reliable due to its reliance on a group of signatures for each cell type, learned from multiple data sources, which increases the ability to distinguish the signal from the noise. Our method also integrates a novel approach to remove dependencies between cell types, which allows better reliability when studying closely related cell types.

To develop xCell, we collected the most comprehensive resource to date of primary cell types, spanning the largest set of human cell types. We then performed an extensive validation of the predicted cell type inferences in mixed samples. Our method for choosing a set of signatures that are reliable across several data sources has proven to be beneficial, as our scores robustly outperformed all available methods in predicting the abundance of cell types in in silico mixtures and blood samples. Based on our evaluation, xCell provides the most accurate and sensitive way to identify enrichment of many cell types in an admixture, allowing the detection of subtle differences in the enrichment of a particular cell type in the tumor microenvironment with high confidence.

It is important to note that xCell, as all other methods, performed significantly better in simulated mixtures than in real mixtures. Several technical reasons account for this discrepancy. First, the cytometry analyses were performed on PBMCs, while the gene expression profiles were generated from whole blood. Second, not all genes required by xCell were present; in fact, in SDY420 only 54.5% of the genes required by xCell were available. However, other explanations for the lower success when inferring abundances in real samples are possible—it may well be possible that the expression patterns of marker genes in mixtures are different to those in purified cells. Recent technologies such as single-cell RNA-sequencing may be able to clarify how much this may perturb the analyses.

We chose to apply a gene signature enrichment approach over deconvolution methods because of several advantages that the former provides. First, gene signatures are rank-based and are therefore suitable for cross-platform transcriptome measurements. We showed here that our scores reliably predict enrichment when using different RNA-seq techniques and different microarray platforms. They are agnostic to normalization methods or concerns related to batch effects, making them robust to both technical and biological noise. Second, there is no decline in performance with increasing numbers of cell types. The tumor microenvironment is a rich milieu of cell types, and our analyses show enrichment of many cells derived from mesenchyme in tumors. A partial portrayal of the tumor microenvironment may result in misleading findings. Finally, gene signatures are simple and can easily be adjusted.

The main disadvantage of gene signatures is that they do not discriminate between closely related cell types well, though it is not clear how well other methods distinguish between such cell types [10]. Our method takes this into account and uses a novel technique, inspired by flow cytometry analyses, to remove such dependencies between closely related cell types. It is important to note that, until this step, the cell type scores are independent of each other, and a false inference of one cell type will not harm all other cell types. However, the spillover correction adjustment removes this strict independence between cell type inferences, as in deconvolution methods. Yet, the compensation is very limited, and between most cell types there is no compensation at all; thus, most of the inferences are still independent.

Despite the utility of our signatures for characterizing the tumor microenvironment, several issues require further investigation. While our signatures outperformed previous methods, it is important to note that our correlations with direct measurements were still far from perfect. More expression data from pure cell types, especially cell types with limited samples, and more expression data coupled with cytometry counts from various tissue types will allow more precise definition of signatures and, in turn, better reliability. Meanwhile, it is necessary to refer to inferences made by our method or other methods with caution. Discoveries made using digital dissection methods must be rigorously validated using other technologies to avoid hasty conclusions.

Another limitation of our method is that the inferences are strictly enrichment scores, and cannot be interpreted as proportions. This is due to the inability to translate the minimum and maximum scores produced by ssGSEA to clear proportions. Thus, while our method attempts to calibrate the scores to resemble proportions, these cannot be reliably used as such. This limitation also does not provide statistical significance for the inferences, by calculating an empirical *p* value as suggested by Newman et al. [7].

## Conclusions

Tissue dissection methods are an emerging tool for large-scale characterization of tumor cellular heterogeneity. These approaches do not rely on tissue dissociation, as opposed to single-cell techniques, and therefore provide an effective tool for dissecting solid tumors. The great availability of public gene expression profiles allows these methods to be efficiently performed on hundreds of historical cohorts spanning thousands of patients, and to associate them with clinical outcomes. Here we present the most comprehensive collection of gene expression enrichment scores for cell types. Our methodology for generating cell type enrichment scores and adjusting them to cell type proportions allowed us to create a powerful tool that is the most reliable and robust tool currently available for identifying cell types across data sources. We provide a simple web tool, xCell (http://xCell.ucsf.edu/), to the community and hope that further studies will utilize it for the discovery of novel predictive and prognostic biomarkers, and new therapeutic targets.

## Methods

### Data sources

#### Signature data sources

RNA-seq and cap analysis gene expression (CAGE) normalized FPKM values were downloaded from the FANTOM5 [33], ENCODE [34], and Blueprint data portals [19]. Raw Affymetrix microarray CEL files were downloaded from the Gene Expression Omnibus (GEO), accessions GSE22886 (IRIS) [35], GSE24759 (Novershtern) [36], and GSE49910 (HPCA) [37], and analyzed using the Robust Multi-array Average (RMA) procedure on probe-level data using Matlab functions. The analysis was performed using custom CDF files downloaded from Brainarray [38]. All samples were manually annotated to 64 cell types (Additional file 1).

#### Other expression data sources

RNA-seq normalized counts were downloaded from the GEO, accession GSE60424 [39]. Illumina HumanHT-12 V4.0 Beadchip data of PBMC samples and the accompanying CyTOF data were downloaded from ImmPort accession SDY311 [40] and quantile normalized using Matlab functions. Similarly, Agilent Whole Human Genome 4 × 44 K slide data of PBMC samples and the accompanying CyTOF data were downloaded from ImmPort accession SDY420 [41] and quantile normalized using Matlab functions. Multiple probes per gene were collapsed using averages. RNA-seq data from the Cancer Cell Line Encyclopedia (CCLE) was obtained using the PharmacoGx R package [42]. RSEM levels for 9947 primary tumor samples from TCGA and TARGET were downloaded from https://toil.xenahubs.net. Published signatures were collected from their corresponding papers [6, 12, 27, 28] (Additional file 5).

### In silico simulated mixtures

We generated several types of simulated mixtures, but all are based on the same pipeline:

1. Given a data source of pure cell types, choose *n* cell types available in the data and choose a random fraction for each cell type (the fractions sum to 1). We denote this vector of fraction *f*.
2. Generate an expression matrix of pure cell types, *M*, with *n* columns. The generation of the expression matrix varied between the experiments we performed: a) Synthetic mixtures for learning the power coefficient and spillover matrix were generated using the median expression profile of each cell type, creating a homogenous and noiseless mixture. b) Training mixtures were generated by randomly choosing one of the multiple available samples for each of the cell types chosen to be included in the mixture. This random selection introduces significant noise into the mixture, and between mixtures in the mixture set, which reflects the variation we observe in real datasets. c) Test mixtures, where only one sample per cell type was available, were generated by adding a random noise for each gene of up to 20% of the expression level. Cell types included in a mixture were chosen randomly, by avoiding cell types that cannot be distinguished (e.g., CD4+ T cells and CD4+ memory T cells).
3. To generate a simulated expression profile we use the formula *M* × *f*, which returns one simulated mixed gene expression profile based on additive expression of the expression profiles of the cell types. This process is then repeated 500 times with different *f* and different *M* (as explained in 2b and 2c, *M* is recreated for each simulation by adding random noise (in b) or choosing a random sample), generating distinct mixtures using the same set of cell types.

### The xCell development pipeline

A workflow of the xCell development pipeline can be found in Additional file 2: Figure S1, and is described in detail below.

#### Filtering cancer genes

In a previous study [16] we calculated using CCLE the number of cell lines that over-express each gene (twofold more than the peak of expression distribution). For generating the signatures we only use genes that have an overexpression rate of less than 5% (less than 32 cell lines of the 634 carcinoma cell lines). We use this stringent threshold to eliminate genes that tend to be overexpressed in tumors, regardless of the cellular composition. Of 18,988 genes analyzed, 9506 were identified as not being overexpressed in tumors. For signatures of cell types that may be the cell of origin of solid tumors, including epithelial cells, sebocytes, keratinocytes, hepatocytes, melanocytes, astrocytes, and neurons, we used all genes.

#### Generating gene signatures

Expression profiles were reduced to 10,808 genes that are shared across all six data sources. Gene expression was converted to log scale by adding 3 to restrict inclusion of small changes and followed by log_2_ conversion. In each group of samples corresponding to a cell type we calculated 10th, 25th, 33.3th, and 50th percentiles of low expression (*Q1*_*q*_), and 90th, 75th, 66.6th, and 50th quantiles of high expression (*Q2*_*1-q*_). For cell type *A* we calculated the difference for each gene between *Q1*_q_(A) and max(*Q2*_1-q_(all other cell types)). We repeated this also for second and third largest *Q2*_1-q_(all other cell types). The signature of cell type *A* consists of all genes that pass a threshold. We used different thresholds here: 0, 0.1, log2(1.5), log(2), 3, 4, and 5. We repeated this procedure for each of the six data sources independently. Only gene sets of at least eight genes and no more than 200 genes were reserved. This scheme yielded 6573 gene signatures corresponding to 64 cell types. We calculated single-sample gene set enrichment analysis (ssGSEA) for each of those signatures to score each sample in each of the data sources using the GSVA R package [43].

#### Choosing the “best” signature

For each signature we computed the t-statistic between the scores of the corresponding cell type compared to all other samples, omitting samples from parental or descendant cell types (i. e. CD4+ naïve T cells the general CD4+ T cells were not used in the calculations). The procedure was performed for each data source where the corresponding cell type was available, except the data source from which the signature was learned. Thus, a signature was only chosen if it is reliable in a data source it was not trained upon. If the cell type was available in only one data source, the signature was tested in that data source. From each data source the top three signatures were chosen. All together we chose 489 signatures corresponding to 64 cell types (across the six data sources we have 163 cell types; Additional file 3). The raw score for a cell type is the average of all corresponding signatures, after shifting scores of each signature to have a minimal score of 0 across all samples.

#### Learning parameters for raw score transformation

For each cell type we created a synthetic mixture using the median expression profile of the cell type (cell *X*) and an additional “control” cell type. For the control in sequencing-based data sources we used multipotent progenitor (MPP) cell samples or endothelial cell samples, because both are found in all the sequencing-based data sets. In microarray-based data sources we used erythrocytes and monocytes. We generated such mixtures using increasing levels of the corresponding cell type (0.8% of cell *X* and 99.2% control, 1.6% cell *X* and 98.4% control, etc.). We noticed two problems with the raw scores: ssGSEA scores have different distributions between different signatures and a score from signatures cannot thus be compared with a score from another signature. In addition, in sequencing-based data, the association between the underlying levels of the cell type was not linearly associated with the score. We thus designed a transformation pipeline for the scores (which is applied to both sequencing and microarray-based datasets separately)—for each cell type, using the synthetic mixtures, we first shifted the scores to 0 using the minimal score (which corresponded to mixtures containing 0.8% of the cell type) and divided by 5000. We then fit a power function to the scores corresponding to abundances of 0.8 to 25.6%. We used this range because we are mostly interested in identifying cell types with low abundance, and above that the function exponential increase may interfere with precise fitting. The power coefficient was then averaged across the data sources were the cell type is available (we denote this vector as *P*). After adjusting the score using the learned power coefficient, we fit a linear curve, and used the learned slope as a calibration parameter for the adjusted scores (denoted as *V*1).

#### Learning the spillover compensation reference matrix

Another limitation that was observed in the mixtures is the dependencies between closely related cell types: scores that predict enrichment of one cell type also predict enrichment of another cell type, which might not even be in the mixture. To overcome this problem we created a reference matrix of “spillovers” between cell types. Below we focus on the generation of the sequencing-based spillover matrix but an equivalent process was performed to generate the microarray-based spillover matrix. We first generated a synthetic mixture set, where each mixture contains 25% of each of the cell types (median expression) and 75% of a “control” cell type, as in the previous section. We then calculated raw cell type scores and transformed them using the learned coefficients as explained above. We combined all sequencing-based data sources together by using the average scores, and completed the matrix to be 64 × 64 by adding columns from cell types that are not present in any of the sequencing-based data using the microarray reference matrix. We then normalized each row of cell type scores by dividing it by the diagonal (denoted as *K*; in the spillover matrix rows are cell type scores and columns are cell type samples). The diagonal, before the normalization, is also used for calibration (denoted as *V*2). The “spillover” between a cell type score (*x*) and another cell type (*y*) is the ratio between *x* and *y*. Finally, we cleaned the spillover matrix to not compensate between parent and descendent cell types by compensating parent cell types only with other parent cell types (CD4+ T cells are compensated against CD8+ T cells, but not CD8+ Tem), and compensating child cell types only compared to other child cell types from the same parent and all other parents, but not child cell types from other parents. Some of the compensations were too strong, removing correlations between cell types and their corresponding signatures; thus, we limited the compensation levels, off the diagonal, to 0.5. The spillover matrix, power, and calibration coefficients are available in Additional file 7.

#### Calculating scores for a mixture

The input comprises a gene expression data set normalized to gene length (such as FPKM or TPM), where rows are genes and columns are samples (N is the number of samples). Duplicate gene names are combined together. xCell uses a set of 10,808 genes for the scoring. It is recommended to use data sets that contain at least the majority of these genes. Missing values in a sample are treated as missing genes (the xCell web tool requires intersection of at least 5000 genes). It is also recommended to use as many samples as possible, with highly expected variation in cell type fractions. (1) ssGSEA scores are calculated for each of the 489 gene signatures. (2) Scores of all signatures corresponding to a cell type are averaged. The result is a matrix (*A*) with 64 rows and N columns. (3) Each element in the scores matrix (*A*_*ij*_) is transformed using the following formula:

$$ {T}_{ij}=\raisebox{1ex}{$\left({A}_{ij}-\min \left({A}_i\right)\right)/5000\Big){}^{P_i}$}\!\left/ \!\raisebox{-1ex}{$\left(V{1}_i\cdot V{2}_i\right)$}\right. $$

The output is matrix *T* of transformed scores. Different P, *V*1, and *V*2 are used for sequencing-based and microarray-based datasets. (4) Spillover compensation is then performed for each row using linear least squares that minimizes the following (as performed in flow cytometry analyses and explained in Bagwell and Adams [25]):

$$ \left\Vert K\cdot x-{T}_i\right\Vert, \mathrm{such}\kern0.17em \mathrm{that}\;x\ge 0 $$

All *x* values are then combined to create the final xCell scores. The compensation may result in deteriorating real associations; thus, we provide a scaling parameter (alpha) to multiply all off-diagonal cells in matrix K. In all experiments in this study we used alpha = 0.5. Different K matrices are used for sequencing-based and array-based data.

#### Significance assessment

We provide a statistical significance assessment for the presence of a cell type in the mixture by learning scores distributions for cell types in random mixtures. For each cell type *X*, we generate a random matrix as follows: In each reference data set we find all cell types corresponding to samples, except *X* and its parent or descendants (if *X* is CD8+ Tem cells, then we also exclude CD8+ T cells; if *X* is CD8+ T cells, we exclude all CD8+ cell types). We then use the same procedure we used for generating training samples, but adding an additional 5% random noise. The main difference here is that we randomly mix in all cell types (except *X*) and not just a small subset. We then run the xCell pipeline for these random mixtures. In most cell types the produced scores show similarity to a beta distribution; thus, using the fitdistr function from the MASS package, we fit such a distribution for each of the mixtures we generated (e.g., for a mixture excluding cell type *X* we fit a beta distribution for cell type *X*). In five of the cell types the scores from the random mixtures consistently produced 0; thus, we define those distributions as constant 0.001 (Additional file 2: Figure S7). Given an input data set, we can now calculate a *p* value for each xCell score with the null hypothesis that the cell type is not present in the mixture. The actual distributions we use to calculate the *p* values are combinations of those learned from FANTOM5, Blueprint, and ENCODE for sequencing-based input, and IRIS, HPCA, and Novershtern for microarray-based input. The *p* value for a score of a cell type in a sample is the chance of the region in the distribution of the corresponding cell type to exceed the score. In the testing samples we used a threshold of 20% to define a non-significant score. We used this threshold to have a trade-off between detecting the non-negligible scores of cell types not in the mixture and not detecting scores of cell type in the mixture, thus affecting the power of estimating the underlying cell type fractions (Additional file 4).

### Cytometry analyses

Gene expression and cytometry data were downloaded from ImmPort (SDY311 [40] and SDY420 [41]). The gene expression data were quantile normalized using Matlab functions, and multiple probes per gene were collapsed using averages. The cytometry data counts were divided by the viable/singlet counts. In the SDY311 dataset, ten patients had two replicates of expression profiles, and those were averaged. Two outlier samples in the cytometry data set were removed from further analyses (SUB134240, SUB134283).

### Other tools

The CIBERSORT web tool was used for inferring proportions using the expression profile (https://cibersort.stanford.edu). CIBERSORT results for activated and resting cell types were combined; B cell and CD4+ T cell percentages are the combination of all their subtypes. t-SNE plots were produced using the Rtsne R package. Purity measurements were obtained from our previous publication [31]. Correlation plots were generated using the corrplot R package.

## Additional files

Additional file 1: Summary table of primary cell types used in this study. (XLSX 51 kb)

Additional file 2: Figures S1–S12. (PDF 7074 kb)

Additional file 3: The 489 cell type gene signatures. (XLSX 417 kb)

Additional file 4: Summary table of the statistical significance analysis in the testing mixtures. (XLSX 50 kb)

Additional file 5: The 53 previously published cell type gene signatures. (XLSX 58 kb)

Additional file 6: xCell scores of 9947 samples from TCGA and TARGET. (TSV 6996 kb)

Additional file 7: The spillover matrix and calibrating coefficients. (XLSX 110 kb)

## References

[1] Galon J, Costes A, Sanchez-Cabo F, Kirilovsky A, Mlecnik B, Lagorce-Pagès C (2006). Type, density, and location of immune cells within human colorectal tumors predict clinical outcome. Science.

[2] Hanahan D, Coussens LM (2012). Accessories to the crime: functions of cells recruited to the tumor microenvironment. Cancer Cell.

[3] Gentles AJ, Newman AM, Liu CL, Bratman SV, Feng W, Kim D (2015). The prognostic landscape of genes and infiltrating immune cells across human cancers. Nat Med.

[4] Abbas AR, Wolslegel K, Seshasayee D, Modrusan Z, Clark HF. Deconvolution of blood microarray data identifies cellular activation patterns in systemic lupus erythematosus. PLoS One. 2009;4:e6098. 10.1371/journal.pone.0006098 PMC2699551 19568420

[5] Shen-Orr SS, Gaujoux R (2013). Computational deconvolution: extracting cell type-specific information from heterogeneous samples. Curr Opin Immunol.

[6] Rooney MS, Shukla SA, Wu CJ, Getz G, Hacohen N (2015). Molecular and genetic properties of tumors associated with local immune cytolytic activity. Cell.

[7] Newman AM, Liu CL, Green MR, Gentles AJ, Feng W, Xu Y (2015). Robust enumeration of cell subsets from tissue expression profiles. Nat Methods.

[8] Newman AM, Alizadeh AA (2016). High-throughput genomic profiling of tumor-infiltrating leukocytes. Curr Opin Immunol.

[9] Angelova M, Charoentong P, Hackl H, Fischer ML, Snajder R, Krogsdam AM (2015). Characterization of the immunophenotypes and antigenomes of colorectal cancers reveals distinct tumor escape mechanisms and novel targets for immunotherapy. Genome Biol.

[10] Li B, Severson E, Pignon J-C, Zhao H, Li T, Novak J (2016). Comprehensive analyses of tumor immunity: implications for cancer immunotherapy. Genome Biol.

[11] Iglesia MD, Parker JS, Hoadley KA, Serody JS, Perou CM, Vincent BG. Genomic Analysis of immune cell infiltrates across 11 tumor types. J Natl Cancer Inst. 2016;108:djw144. 10.1093/jnci/djw144 PMC5241901 27335052

[12] Charoentong P, Finotello F, Angelova M, Mayer C, Efremova M, Rieder D (2017). Pan-cancer immunogenomic analyses reveal genotype-immunophenotype relationships and predictors of response to checkpoint blockade. Cell Rep.

[13] Şenbabaoğlu Y, Gejman RS, Winer AG, Liu M, Van Allen EM, de Velasco G (2016). Tumor immune microenvironment characterization in clear cell renal cell carcinoma identifies prognostic and immunotherapeutically relevant messenger RNA signatures. Genome Biol.

[14] Pattabiraman DR, Weinberg RA (2014). Tackling the cancer stem cells--what challenges do they pose?. Nat Rev Drug Discov.

[15] Turley SJ, Cremasco V, Astarita JL (2015). Immunological hallmarks of stromal cells in the tumour microenvironment. Nat Rev Immunol.

[16] Aran D, Lasry A, Zinger A, Biton M, Pikarsky E, Hellman A (2016). Widespread parainflammation in human cancer. Genome Biol BioMed Central.

[17] Lizio M, Harshbarger J, Shimoji H, Severin J, Kasukawa T, Sahin S (2015). Gateways to the FANTOM5 promoter level mammalian expression atlas. Genome Biol.

[18] Consortium EP, Bernstein BE, Birney E, Dunham I, Green ED, Gunter C (2012). An integrated encyclopedia of DNA elements in the human genome. Nature.

[19] Blueprint Epigenome Project. 2015. http://www.blueprint-epigenome.eu/. Accessed 3 May 2016.

[20] Abbas AR, Baldwin D, Ma Y, Ouyang W, Gurney A, Martin F (2005). Immune response in silico (IRIS): immune-specific genes identified from a compendium of microarray expression data. Genes Immune.

[21] Novershtern N, Subramanian A, Lawton LN, Mak RH, Haining WN, McConkey ME (2011). Densely interconnected transcriptional circuits control cell states in human hematopoiesis. Cell.

[22] Mabbott NA, Baillie JK, Brown H, Freeman TC, Hume DA (2013). An expression atlas of human primary cells: inference of gene function from coexpression networks. BMC Genomics.

[23] Barretina J, Caponigro G, Stransky N, Venkatesan K, Margolin AA, Kim S (2012). The Cancer Cell Line Encyclopedia enables predictive modelling of anticancer drug sensitivity. Nature.

[24] Barbie DA, Tamayo P, Boehm JS, Kim SY, Moody SE, Dunn IF (2009). Systematic RNA interference reveals that oncogenic KRAS-driven cancers require TBK1. Nature.

[25] Bagwell CB, Adams EG (1993). Fluorescence spectral overlap compensation for any number of flow cytometry parameters. Ann N Y Acad Sci.

[26] Linsley PS, Speake C, Whalen E, Chaussabel D. Copy number loss of the interferon gene cluster in melanomas is linked to reduced T cell infiltrate and poor patient prognosis. PLoS One. 2014;9:e109760. 10.1371/journal.pone.0109760 PMC4196925 25314013

[27] Bindea G, Mlecnik B, Tosolini M, Kirilovsky A, Waldner M, Obenauf AC (2013). Spatiotemporal dynamics of intratumoral immune cells reveal the immune landscape in human cancer. Immunity.

[28] Tirosh I, Izar B, Prakadan SM, Wadsworth MH, Treacy D, Trombetta JJ (2016). Dissecting the multicellular ecosystem of metastatic melanoma by single-cell RNA-seq. Science.

[29] Bhattacharya S, Andorf S, Gomes L, Dunn P, Schaefer H, Pontius J (2014). ImmPort: Disseminating data to the public for the future of immunology. Immunol Res.

[30] Vivian J, Rao AA, Nothaft FA, Ketchum C, Armstrong J, Novak A (2017). Toil enables reproducible, open source, big biomedical data analyses. Nat Biotechnol.

[31] Aran D, Sirota M, Butte AJ (2015). Systematic pan-cancer analysis of tumour purity. Nat Commun.

[32] van der Maaten L, Hinton GE (2008). Visualizing high-dimensional data using t-SNE. J Mach Learn Res.

[33] FANTOM5 project. http://fantom.gsc.riken.jp/5/. Accessed 2 May 2016.

[34] ENCODE: Encyclopedia of DNA Elements. https://www.encodeproject.org/. Accessed 5 May 2016.

[35] Abbas AR et al. Expression profiles from a variety of resting and activated human immune cells. 2010. https://www.ncbi.nlm.nih.gov/geo/query/acc.cgi?acc=GSE22886. Accessed 7 Nov 2014.

[36] Novershtern N et al. Densely interconnected transcriptional circuits control cell states in human hematopoiesis. 2011. https://www.ncbi.nlm.nih.gov/geo/query/acc.cgi?acc=GSE24759. Accessed 11 Nov 2014. 10.1016/j.cell.2011.01.004 PMC3049864 21241896

[37] Mabbott NA et al. An Expression Atlas of Human Primary Cells: Inference of Gene Function from Coexpression Networks. 2013. https://www.ncbi.nlm.nih.gov/geo/query/acc.cgi?acc=GSE49910. Accessed 8 July 2016. 10.1186/1471-2164-14-632 PMC3849585 24053356

[38] Dai M, Wang P, Boyd AD, Kostov G, Athey B, Jones EG, et al. Evolving gene/transcript definitions significantly alter the interpretation of GeneChip data. Nucleic Acids Res. 2005;33:e175. 10.1093/nar/gni179 PMC1283542 16284200

[39] Speake C et al. Next generation sequencing of human immune cell subsets across diseases. 2015. https://www.ncbi.nlm.nih.gov/geo/query/acc.cgi?acc=GSE60424. Accessed 5 Jan 2017.

[40] Immport. 2010. http://www.immport.org/immport-open/public/study/study/displayStudyDetail/SDY311. Accessed 17 July 2016.

[41] Immport. 2010. http://www.immport.org/immport-open/public/study/study/displayStudyDetail/SDY420.

[42] Smirnov P, Safikhani Z, El-Hachem N, Wang D, She A, Olsen C (2016). PharmacoGx: an R package for analysis of large pharmacogenomic datasets. Bioinformatics.

[43] Hänzelmann S, Castelo R, Guinney J (2013). GSVA: gene set variation analysis for microarray and RNA-seq data. BMC Bioinformatics.

[44] Aran D (2017). xCell R package and development scripts.

