# Supplementary material for: xCell: digitally portraying the tissue cellular heterogeneity landscape
Source: Genome Biol. 2017 Nov 15;18:220. doi: 10.1186/s13059-017-1349-1 (PMC5688663; doi:10.1186/s13059-017-1349-1)
Supplement: Supplementary file 2 — (PDF 7074 kb) [file 13059_2017_1349_MOESM2_ESM.pdf]

Supplementary Figures 1-12
Table of Contents

Supplementary Figure 1. The xCell development pipeline 2

Supplementary Figure 2. Raw scores of the multiple signatures in the test samples 3

Supplementary Figure 3. Simulated mixtures of pure cell types inferred by raw xCell scores 13

Supplementary Figure 4. Transformation procedure of raw scores to linear scales 16

Supplementary Figure 5. Cell types inferences in gene expression simulations using training samples 19

Supplementary Figure 6. Cell types inferences in gene expression simulations using testing samples 30

Supplementary Figure 7. Distributions of cell types' scores from random mixtures 49

Supplementary Figure 8. Dependencies between CD8+ T-cells and NK cells 55

Supplementary Figure 9. CD8+ T-cells scores vs. CD8A expression in cancer cell lines 56

Supplementary Figure 10. xCell scores in 24 TCGA cancer types 57

Supplementary Figure 11. Purity estimations using xCell scores 64

Supplementary Figure 12. t-SNE plots based on cell types scores 65

Supplementary Figure 1:
The xCell development pipeline

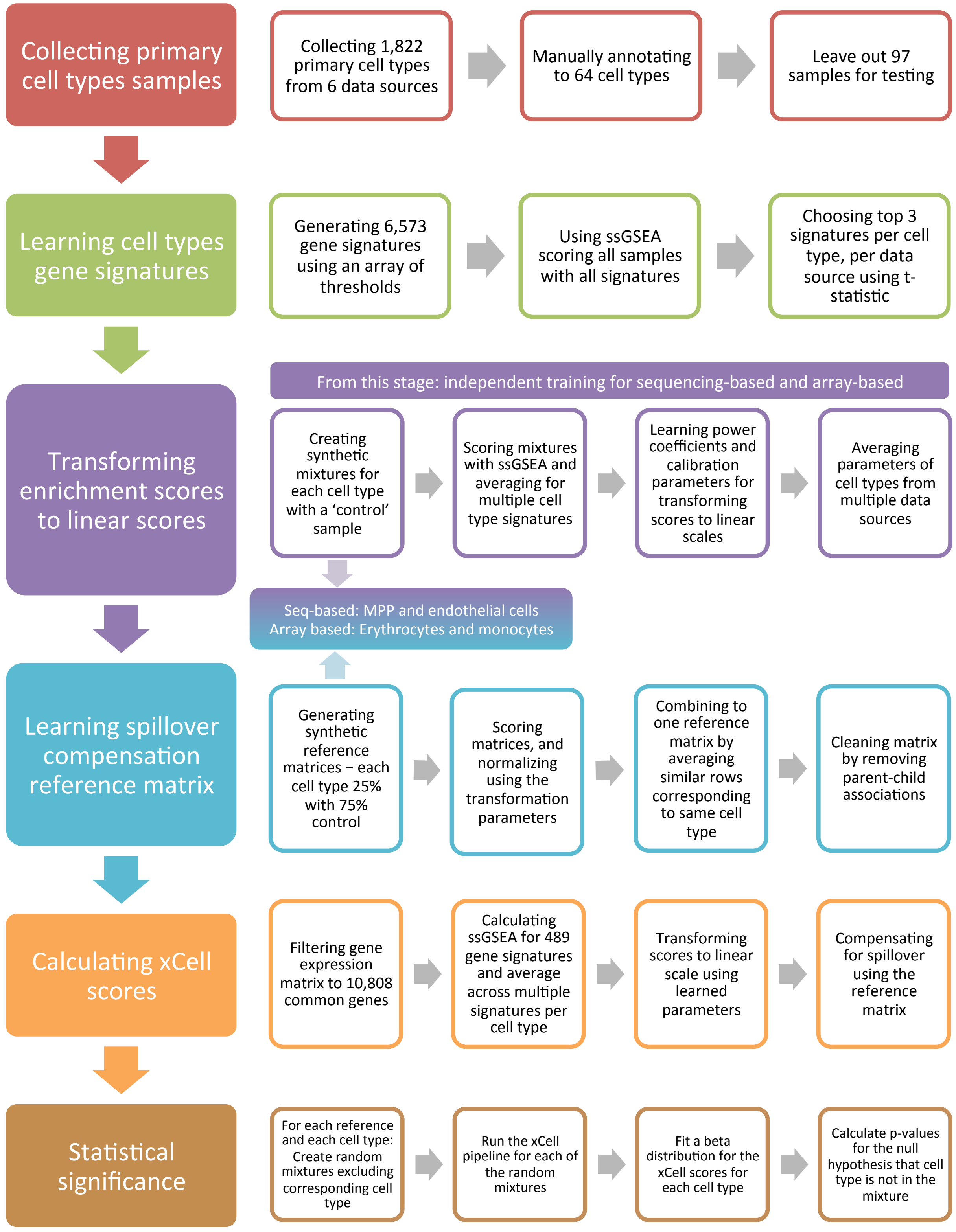

Supplementary Figure 2:
Raw scores of the multiple signatures in the test samples

Sequencing-based testing samples

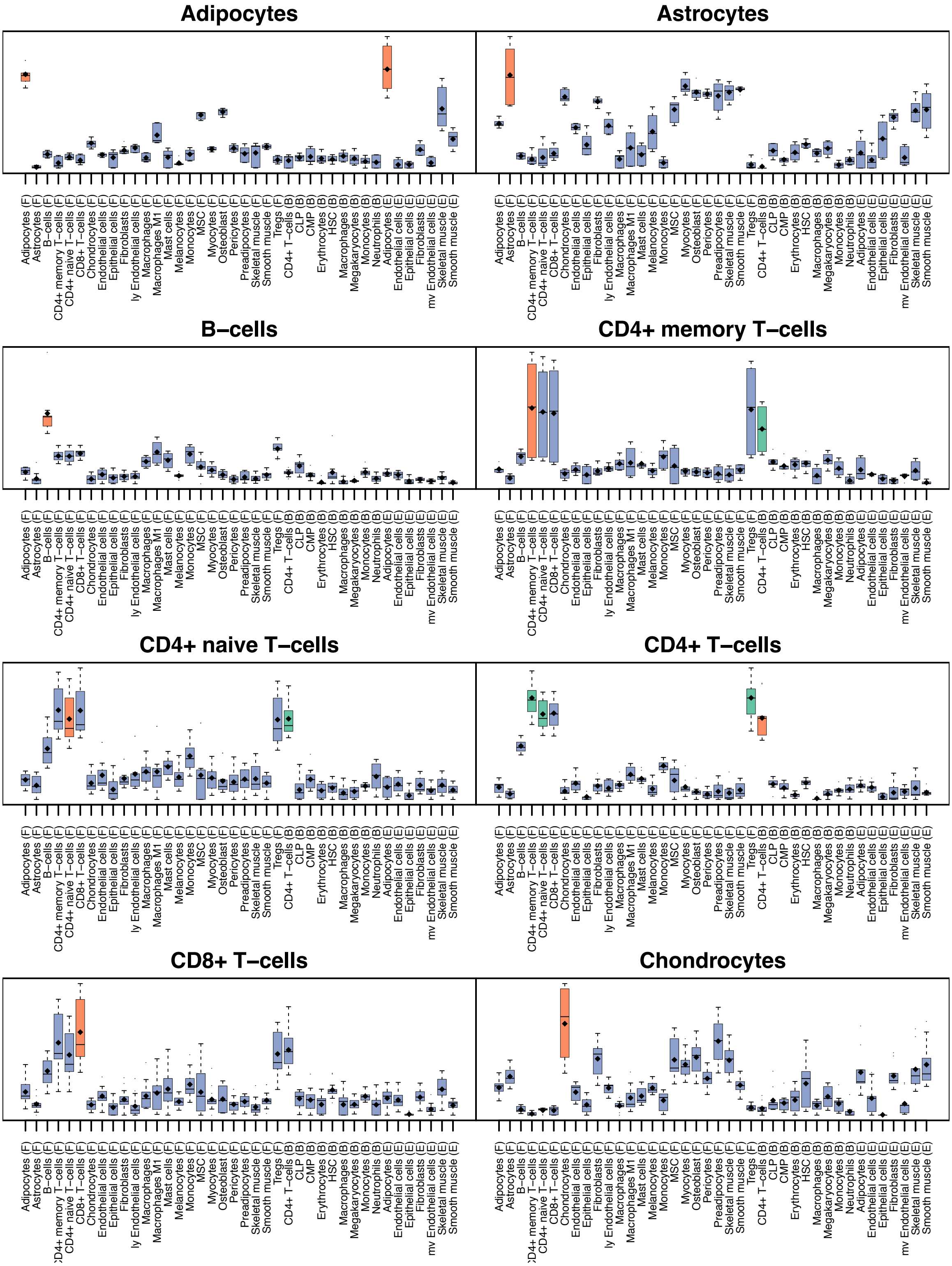

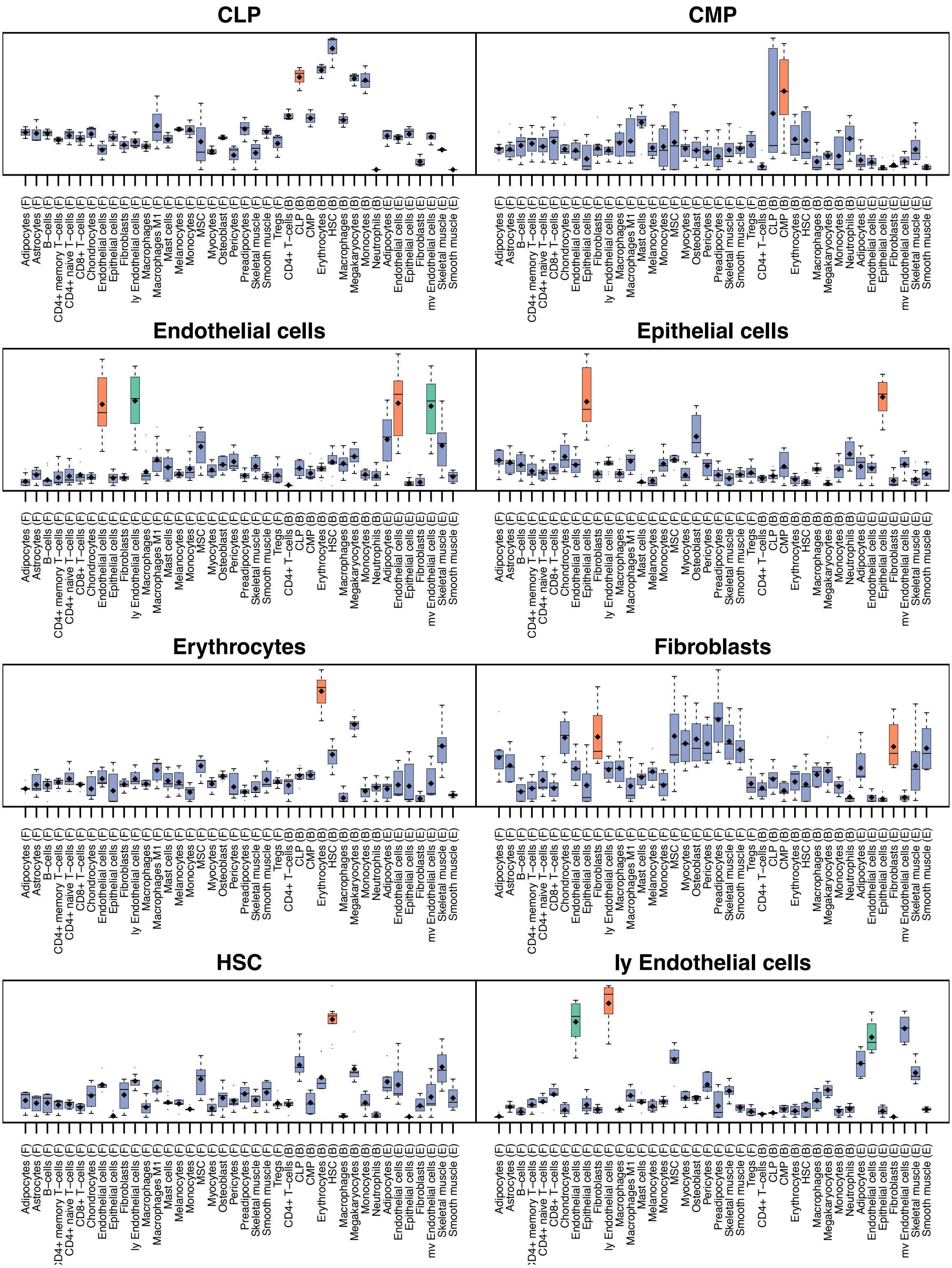
CLP
CMP
Endothelial cells
Epithelial cells
Erythrocytes
Fibroblasts
HSC
ly Endothelial cells
Adipocytes (F)
Astrocytes (F)
B−cells (F)
CD4+ memory T−cells (F)
CD4+ naive T−cells (F)
CD8+ T−cells (F)
Chondrocytes (F)
Endothelial cells (F)
Epithelial cells (F)
Fibroblasts (F)
ly Endothelial cells (F)
Macrophages (F)
Macrophages M1 (F)
Mast cells (F)
Melanocytes (F)
Monocytes (F)
MSC (F)
Myocytes (F)
Osteoblast (F)
Pericytes (F)
Preadipocytes (F)
Skeletal muscle (F)
Smooth muscle (F)
Tregs (F)
CD4+ T−cells (B)
CLP (B)
CMP (B)
Erythrocytes (B)
HSC (B)
Macrophages (B)
Megakaryocytes (B)
Monocytes (B)
Neutrophils (B)
Adipocytes (E)
Endothelial cells (E)
Epithelial cells (E)
Fibroblasts (E)
mv Endothelial cells (E)
Skeletal muscle (E)
Smooth muscle (E)

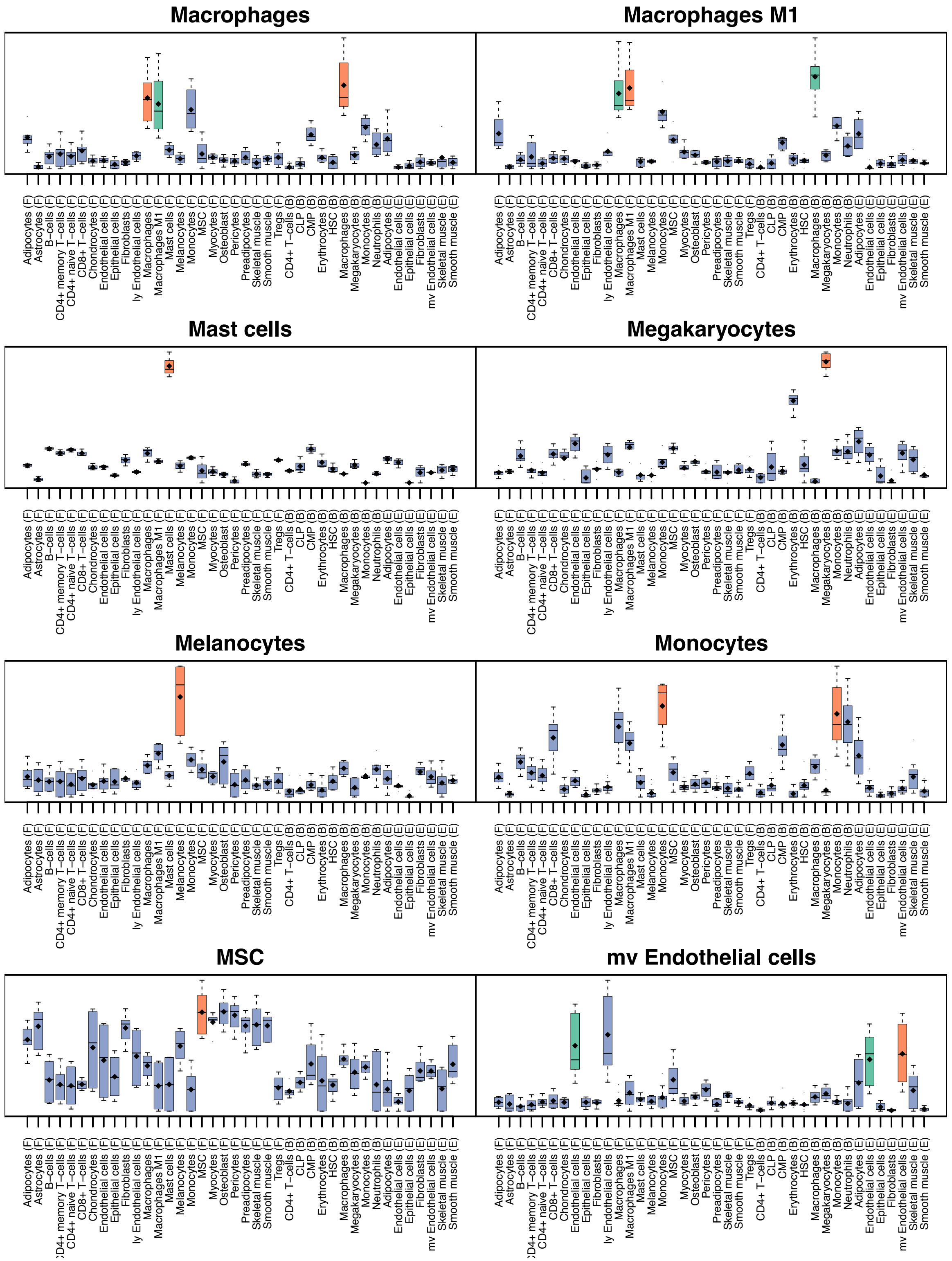
Macrophages
Macrophages M1
Mast cells
Megakaryocytes
Melanocytes
Monocytes
MSC
mv Endothelial cells
Adipocytes (F)
Astrocytes (F)
B−cells (F)
CD4+ memory T−cells (F)
CD4+ naive T−cells (F)
CD8+ T−cells (F)
Chondrocytes (F)
Endothelial cells (F)
Epithelial cells (F)
Fibroblasts (F)
ly Endothelial cells (F)
Macrophages (F)
Macrophages M1 (F)
Mast cells (F)
Melanocytes (F)
Monocytes (F)
MSC (F)
Myocytes (F)
Osteoblast (F)
Pericytes (F)
Preadipocytes (F)
Skeletal muscle (F)
Smooth muscle (F)
Tregs (F)
CD4+ T−cells (B)
CLP (B)
CMP (B)
Erythrocytes (B)
HSC (B)
Macrophages (B)
Megakaryocytes (B)
Monocytes (B)
Neutrophils (B)
Adipocytes (E)
Endothelial cells (E)
Epithelial cells (E)
Fibroblasts (E)
mv Endothelial cells (E)
Skeletal muscle (E)
Smooth muscle (E)

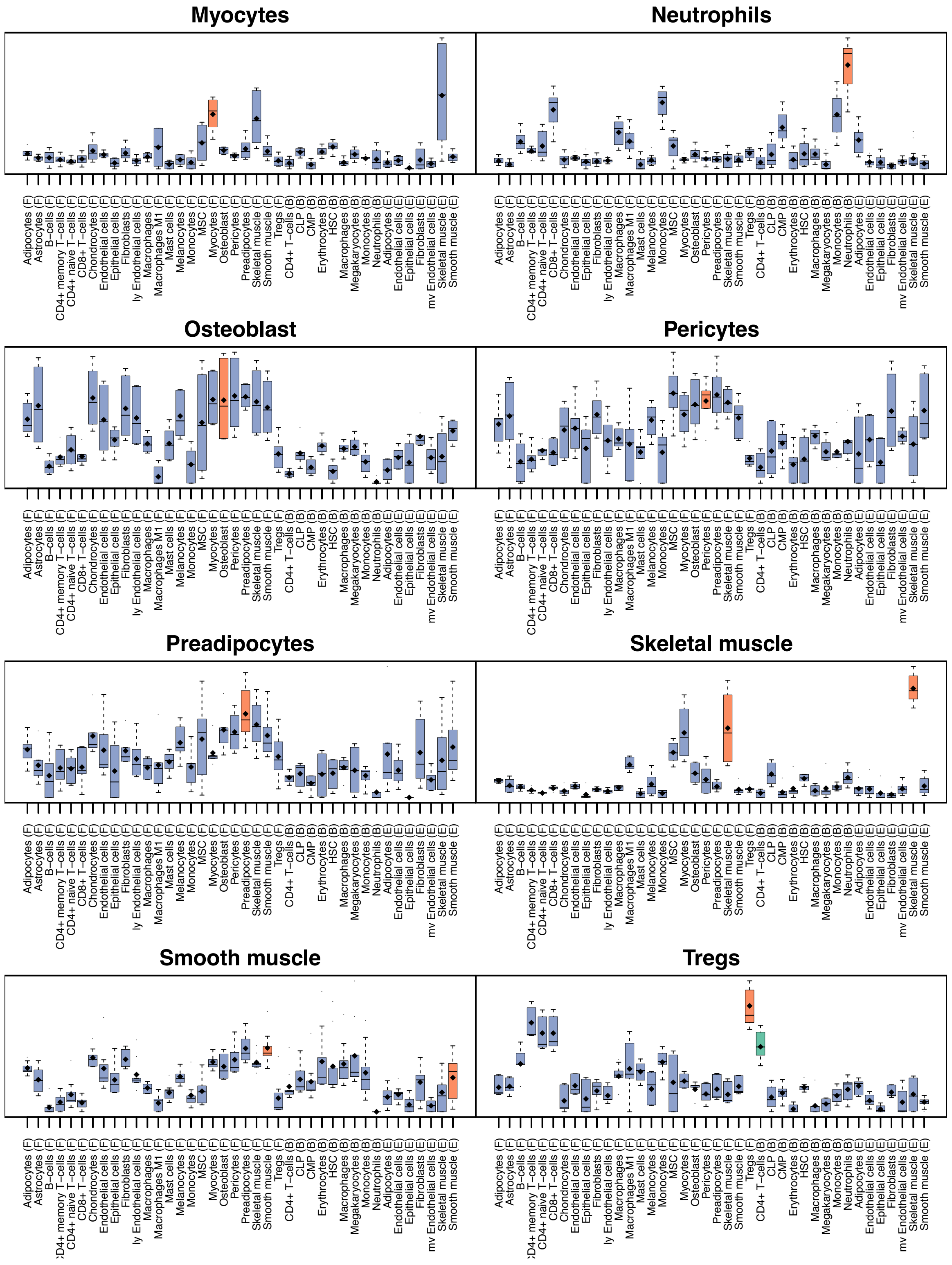
Myocytes
Neutrophils
Osteoblast
Pericytes
Preadipocytes
Skeletal muscle
Smooth muscle
Tregs
Adipocytes (F)
Astrocytes (F)
B−cells (F)
CD4+ memory T−cells (F)
CD4+ naive T−cells (F)
CD8+ T−cells (F)
Chondrocytes (F)
Endothelial cells (F)
Epithelial cells (F)
Fibroblasts (F)
ly Endothelial cells (F)
Macrophages (F)
Macrophages M1 (F)
Mast cells (F)
Melanocytes (F)
Monocytes (F)
MSC (F)
Myocytes (F)
Osteoblast (F)
Pericytes (F)
Preadipocytes (F)
Skeletal muscle (F)
Smooth muscle (F)
Tregs (F)
CD4+ T−cells (B)
CLP (B)
CMP (B)
Erythrocytes (B)
HSC (B)
Macrophages (B)
Megakaryocytes (B)
Monocytes (B)
Neutrophils (B)
Adipocytes (E)
Endothelial cells (E)
Epithelial cells (E)
Fibroblasts (E)
mv Endothelial cells (E)
Skeletal muscle (E)
Smooth muscle (E)

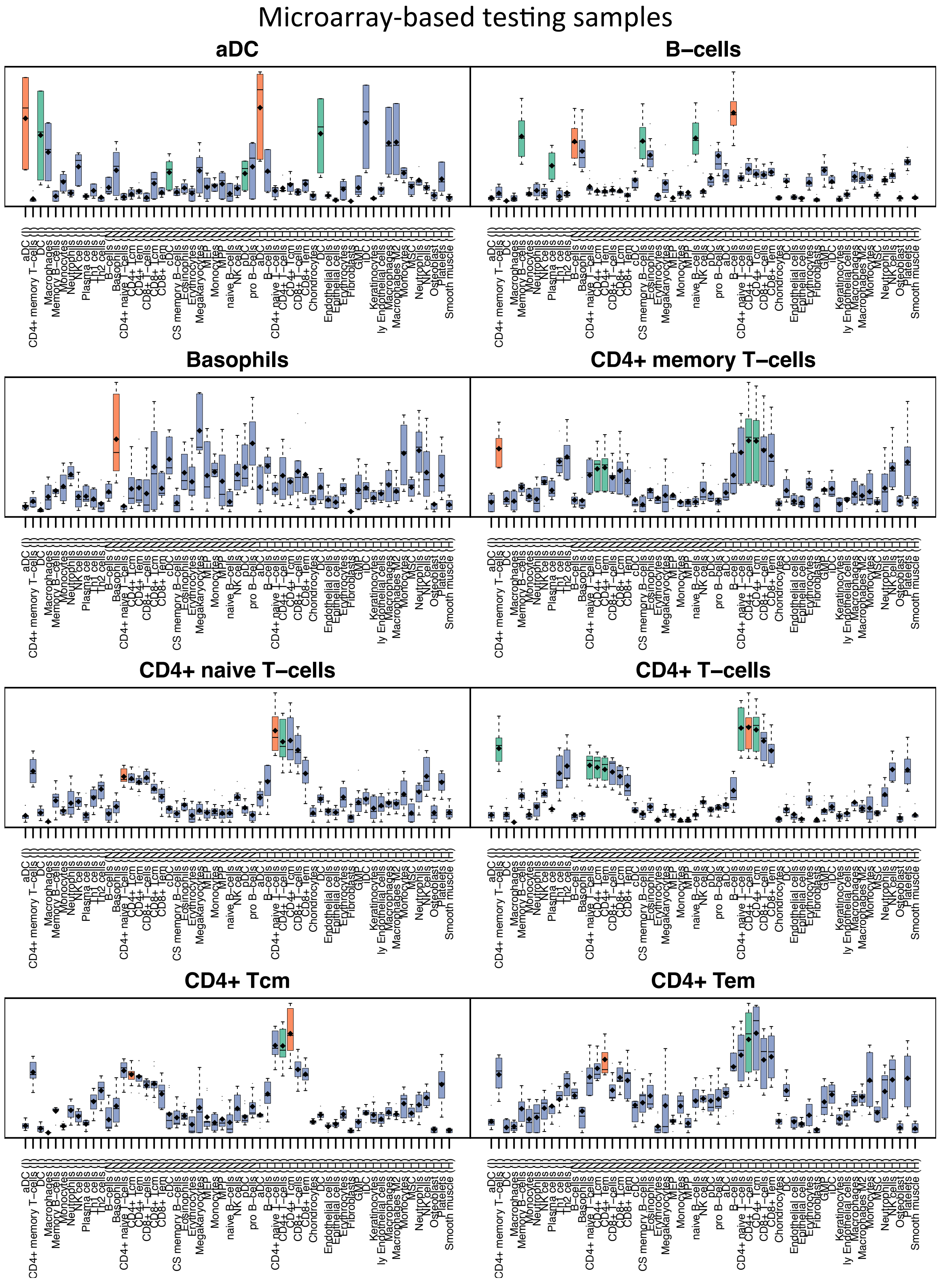
Microarray-based testing samples
aDC
B−cells
Basophils
CD4+ memory T−cells
CD4+ naive T−cells
CD4+ T−cells
CD4+ Tcm
CD4+ Tem

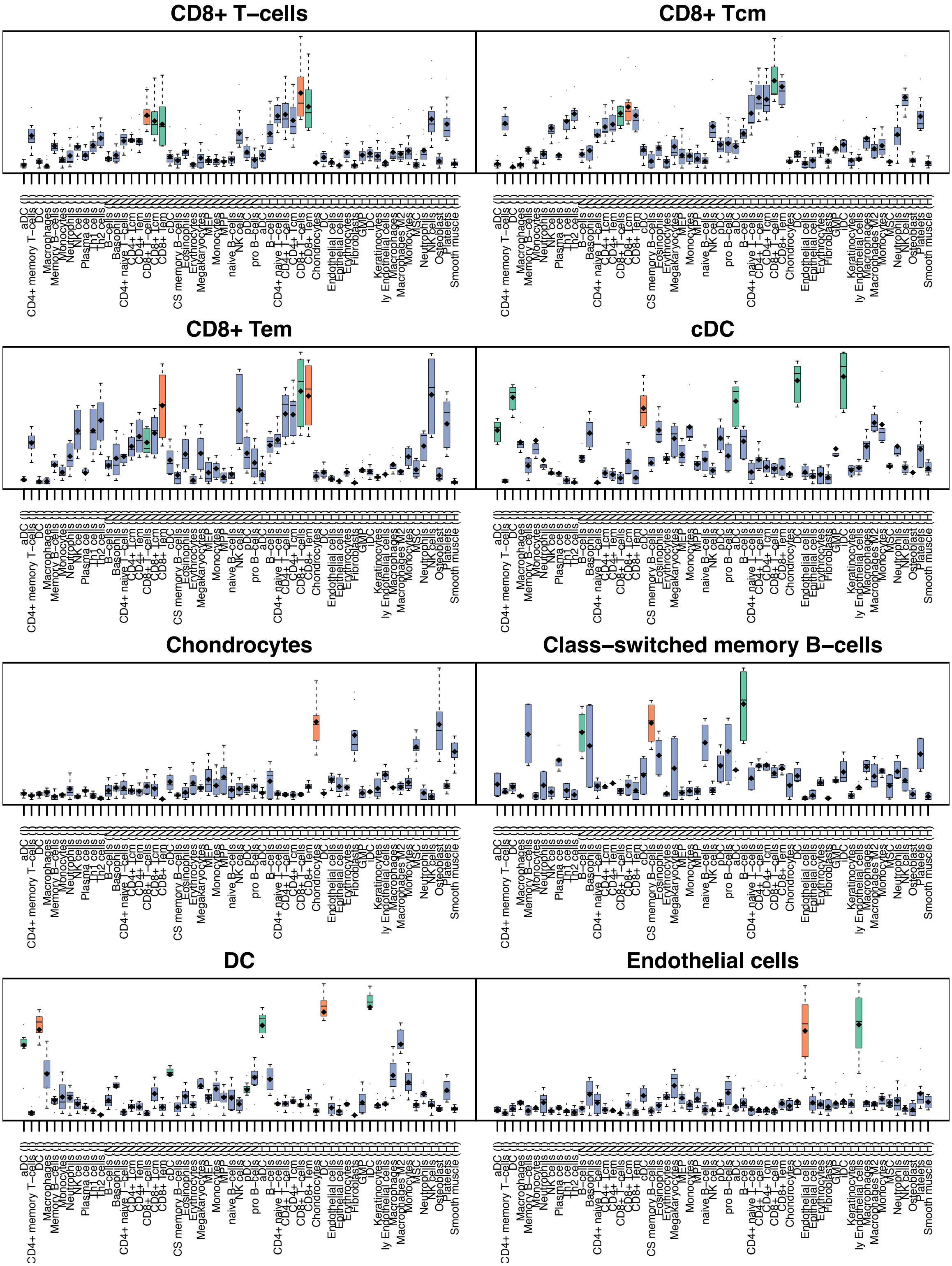
CD8+ T−cells
CD8+ Tcm
CD8+ Tem
cDC
Chondrocytes
Class−switched memory B−cells
DC
Endothelial cells

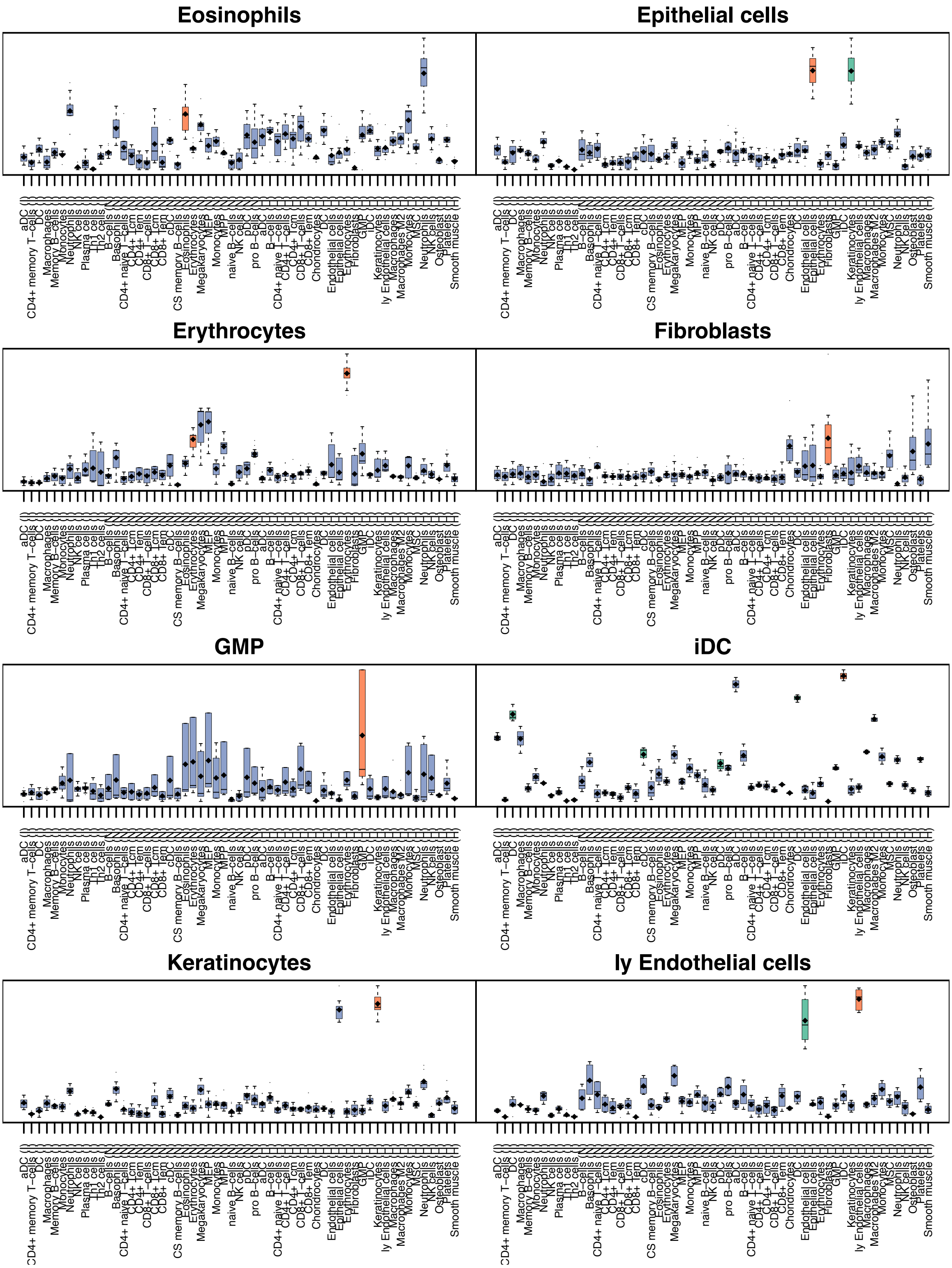

Eosinophils
Epithelial cells
Erythrocytes
Fibroblasts
GMP
iDC
Keratinocytes
ly Endothelial cells

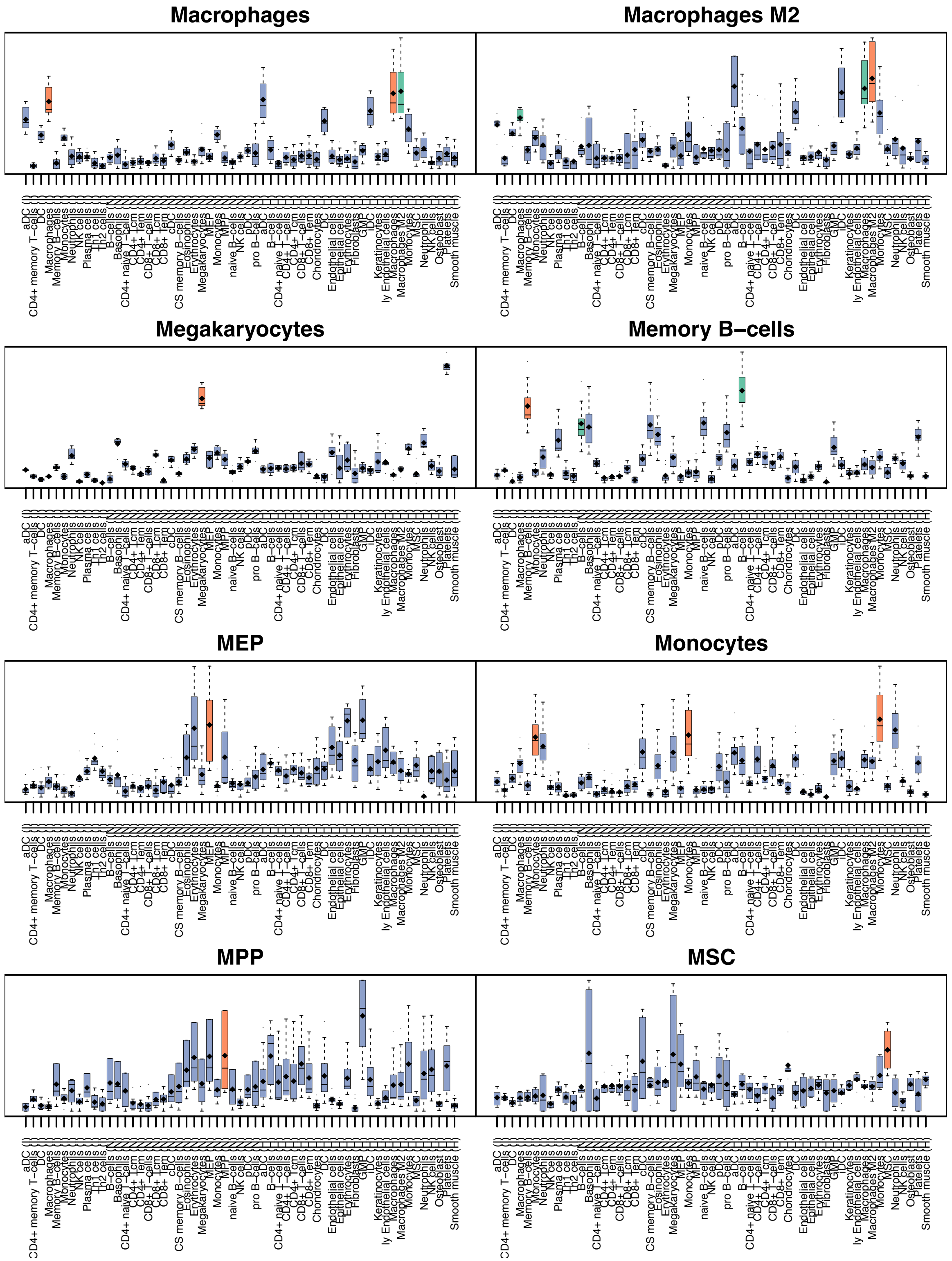
Macrophages
Macrophages M2
Megakaryocytes
Memory B−cells
MEP
Monocytes
MPP
MSC

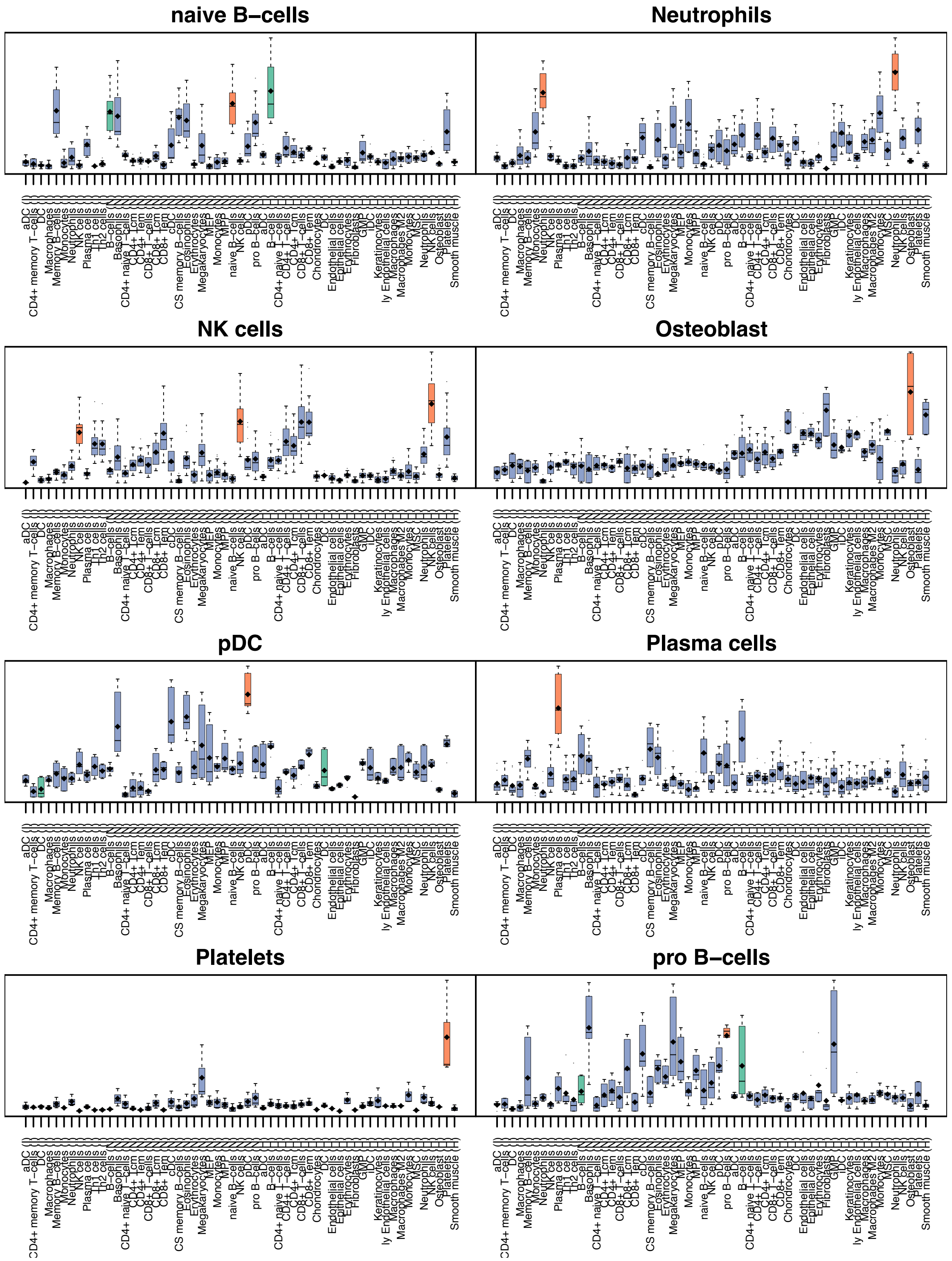
naive B−cells
Neutrophils
NK cells
Osteoblast
pDC
Plasma cells
Platelets
pro B−cells

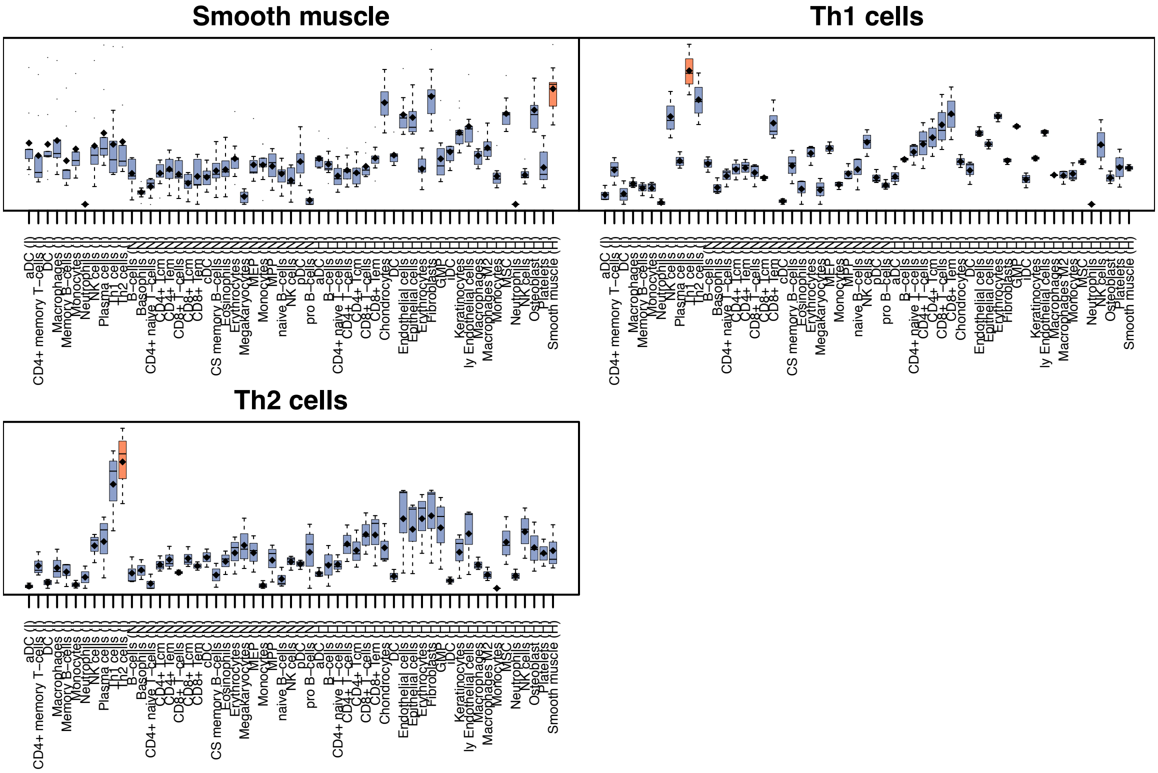

Figure S2. Raw scores of the multiple signatures in the test samples. Each box plot shows the ssGSEA scores for a given set of a cell type's signatures (the signatures that correspond to the cell type presented in the title of the plot). The scores are shown on the 40 sequencing-based and 57 microarray-based left-out samples, which were not used for generating the signatures. In source of the sample is in parenthesis(F − FANTOM5, E − Encode, B − Blueprint, I − IRIS, N − Novershtern, H − HPCA). Box plots were colored to emphasize the cell type of interest (red), and its parental/descendants (green). Average of the scores is presented with a black circle. xCell uses the average of the multiple signatures in downstream analyses.

Supplementary Figure 3:
Simulated mixtures of pure cell types inferred by raw xCell scores

Blueprint – simulated mixture of 12 cell types

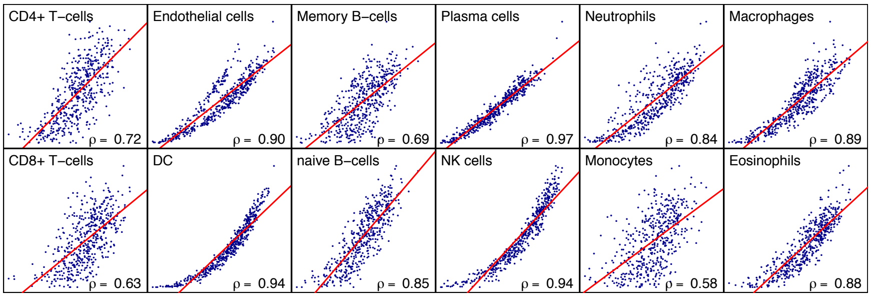

Blueprint – simulated mixture of 14 cell types

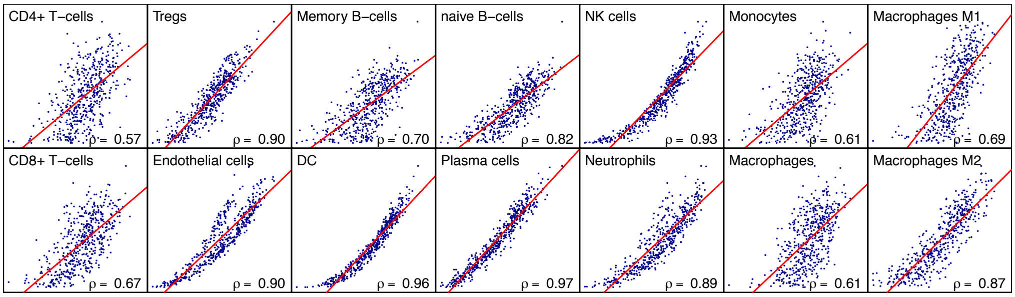

Blueprint – simulated mixture of 16 cell types

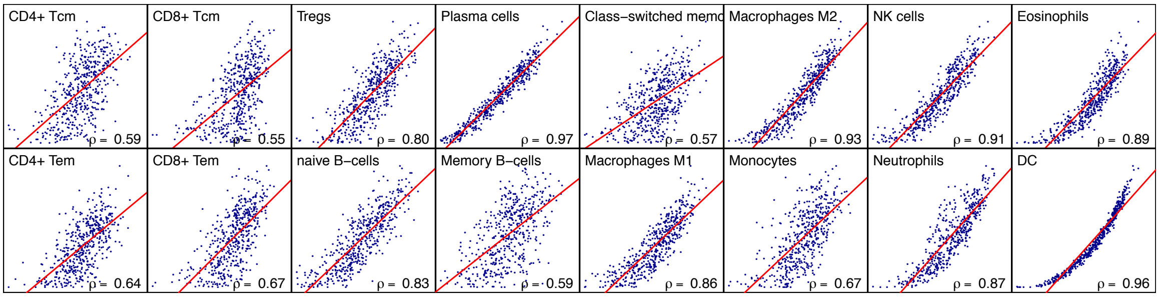

ENCODE – simulated mixture of 10 cell types

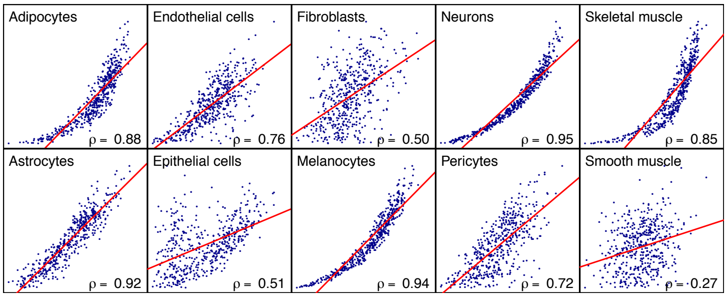

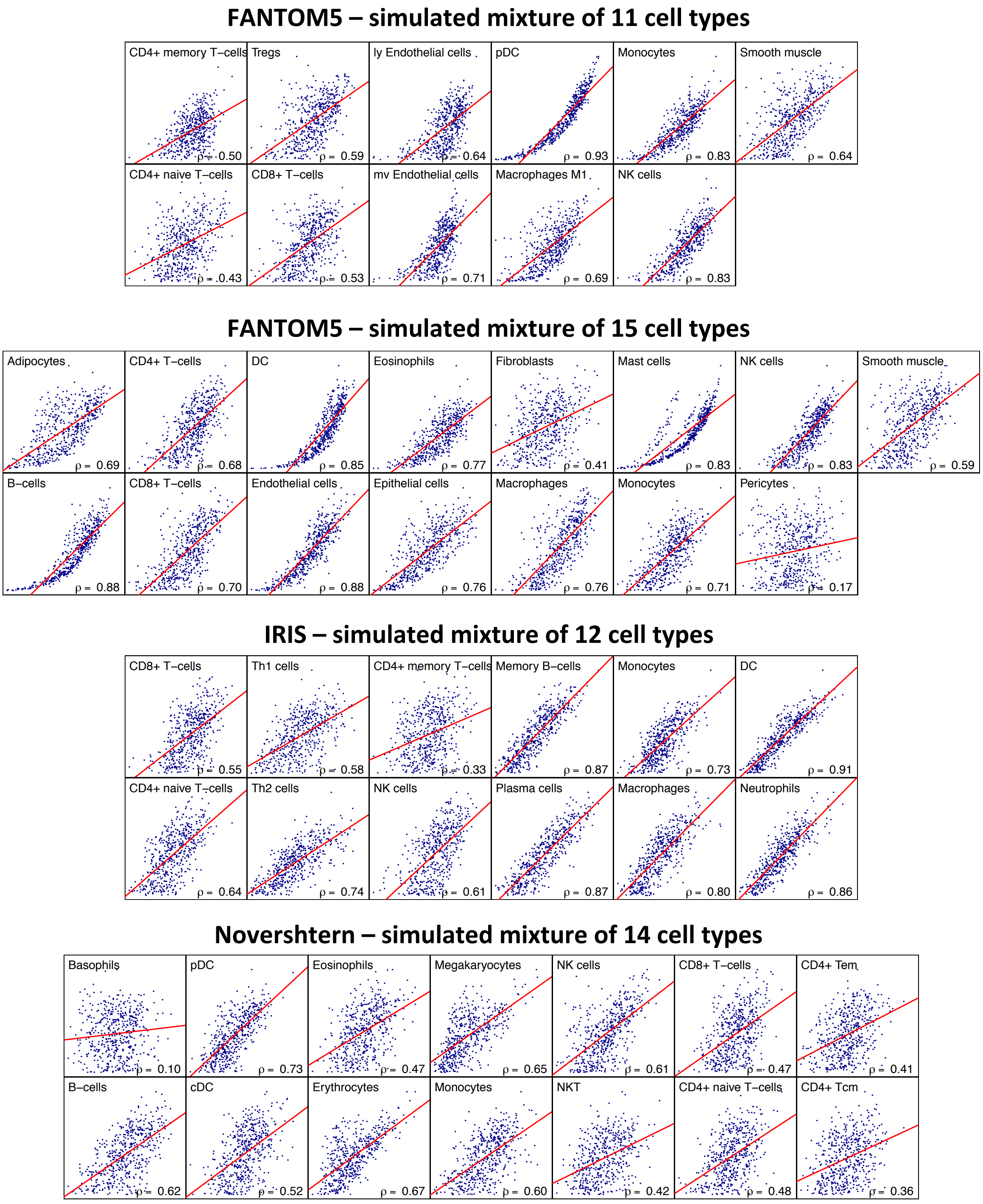
FANTOM5 – simulated mixture of 11 cell types
CD4+ memory T−cells ρ = 0.50
Tregs ρ = 0.59
ly Endothelial cells ρ = 0.64
pDC ρ = 0.93
Monocytes ρ = 0.83
Smooth muscle ρ = 0.64
CD4+ naive T−cells ρ = 0.43
CD8+ T−cells ρ = 0.53
mv Endothelial cells ρ = 0.71
Macrophages M1 ρ = 0.69
NK cells ρ = 0.83
FANTOM5 – simulated mixture of 15 cell types
Adipocytes ρ = 0.69
CD4+ T−cells ρ = 0.68
DC ρ = 0.85
Eosinophils ρ = 0.77
Fibroblasts ρ = 0.41
Mast cells ρ = 0.83
NK cells ρ = 0.83
Smooth muscle ρ = 0.59
B−cells ρ = 0.88
CD8+ T−cells ρ = 0.70
Endothelial cells ρ = 0.88
Epithelial cells ρ = 0.76
Macrophages ρ = 0.76
Monocytes ρ = 0.71
Pericytes ρ = 0.17
IRIS – simulated mixture of 12 cell types
CD8+ T−cells ρ = 0.55
Th1 cells ρ = 0.58
CD4+ memory T−cells ρ = 0.33
Memory B−cells ρ = 0.87
Monocytes ρ = 0.73
DC ρ = 0.91
CD4+ naive T−cells ρ = 0.64
Th2 cells ρ = 0.74
NK cells ρ = 0.61
Plasma cells ρ = 0.87
Macrophages ρ = 0.80
Neutrophils ρ = 0.86
Novershtern – simulated mixture of 14 cell types
Basophils ρ = 0.10
pDC ρ = 0.73
Eosinophils ρ = 0.47
Megakaryocytes ρ = 0.65
NK cells ρ = 0.61
CD8+ T−cells ρ = 0.47
CD4+ Tem ρ = 0.41
B−cells ρ = 0.62
cDC ρ = 0.52
Erythrocytes ρ = 0.67
Monocytes ρ = 0.60
NKT ρ = 0.42
CD4+ naive T−cells ρ = 0.48
CD4+ Tcm ρ = 0.36

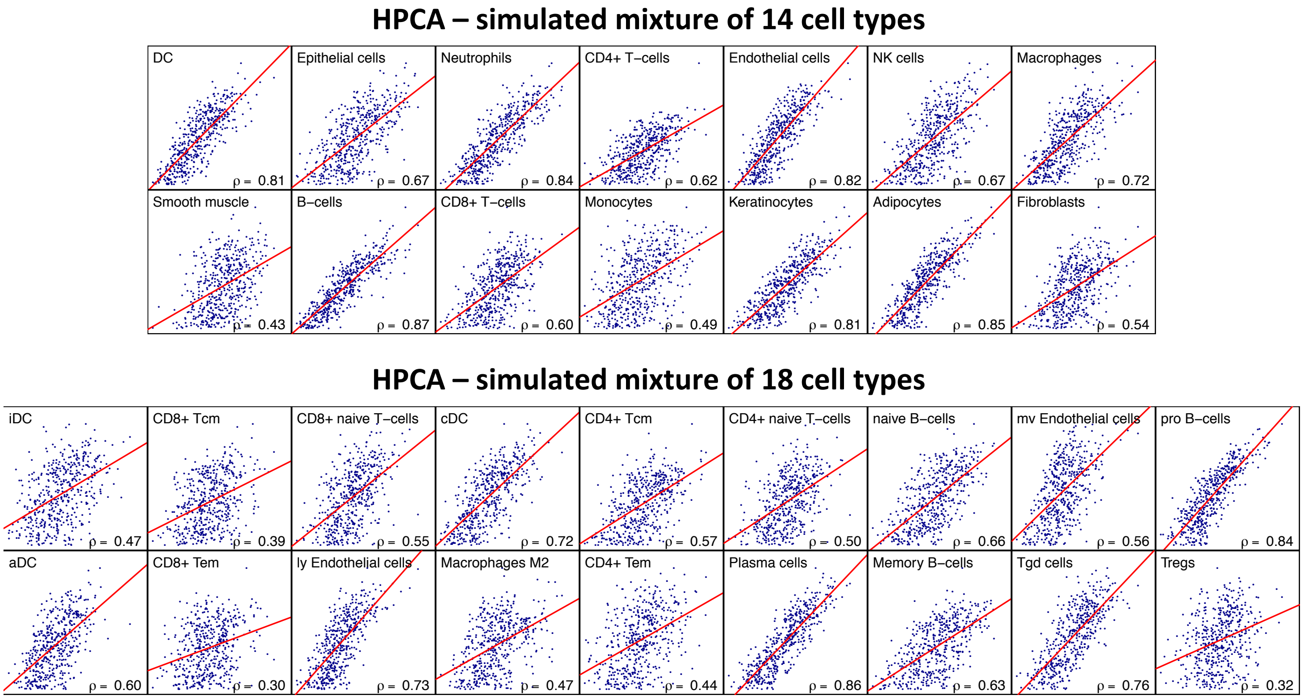

Figure S3. Simulated mixtures of pure cell types inferred by raw xCell scores. Each set of scatter plots represents 500 simulated mixtures of gene expression profiles of pure cell types chosen randomly from the training set. The simulation is performed as following: we chose a data source and a set of cell types that are found in this data source. For each of the 500 mixtures one of the several samples of the cell type is chosen by random, and its expression profile is then multiplied by a random fraction. The expression profile of the mixture is the sum of expression all cell types presented in the mixture. Each scatter plot shows the inferred raw score by xCell (average of all the cell type's corresponding signatures) in the x-axis compared to the underlying fraction of the cell type. Pearson correlation is presented. The x-axis is an enrichment score, and presents the full range of the scores, but in each cell type this range may be completely different. The plots show that in most cell types the raw enrichment scores are reliable in predict even small changes in the proportions of cell types. We notice however that in the sequencing-based data sources the association between the scores and the abundances are not linear.

Supplementary Figure 4:
Transformation procedure of raw scores to linear scales

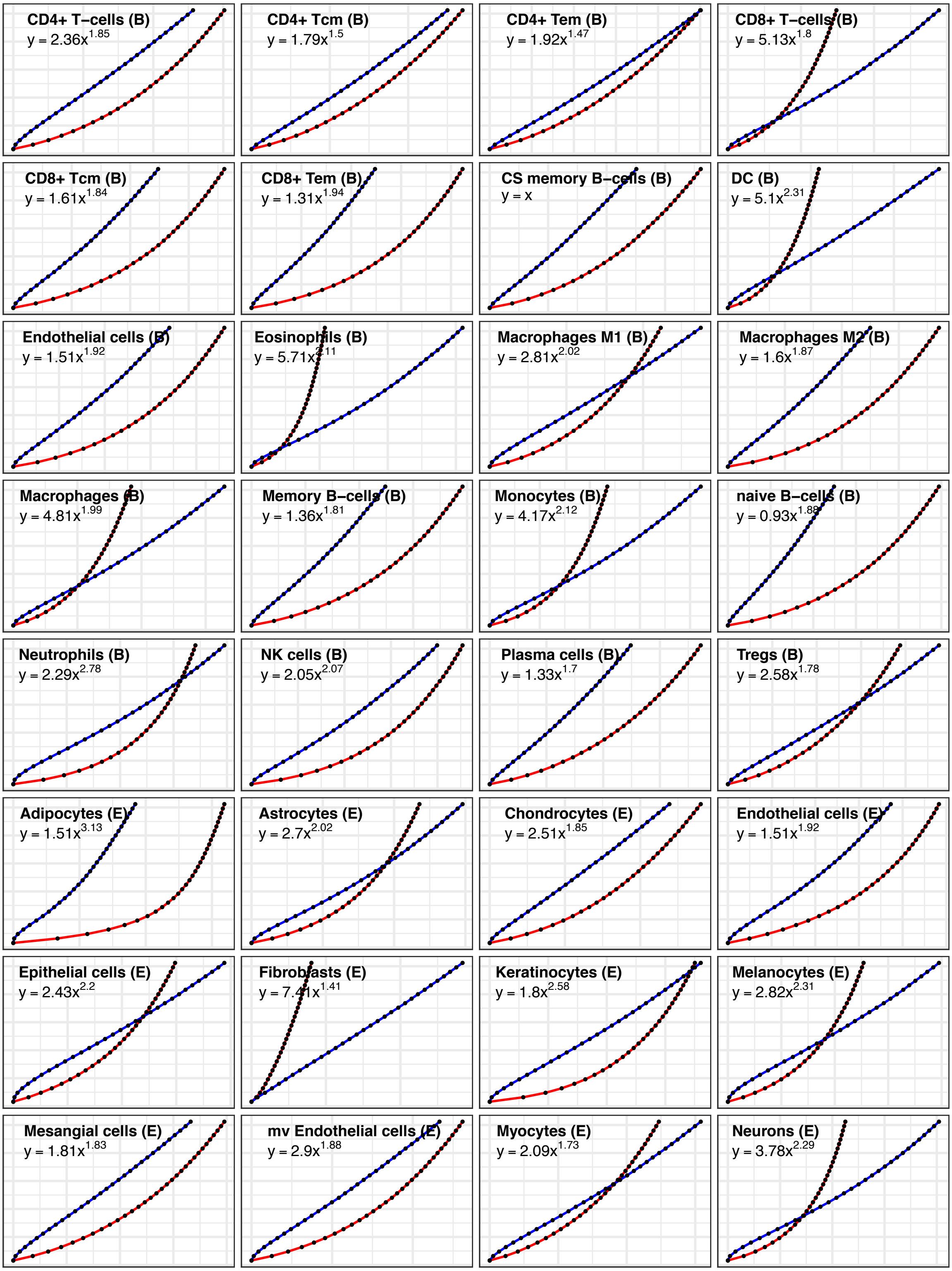

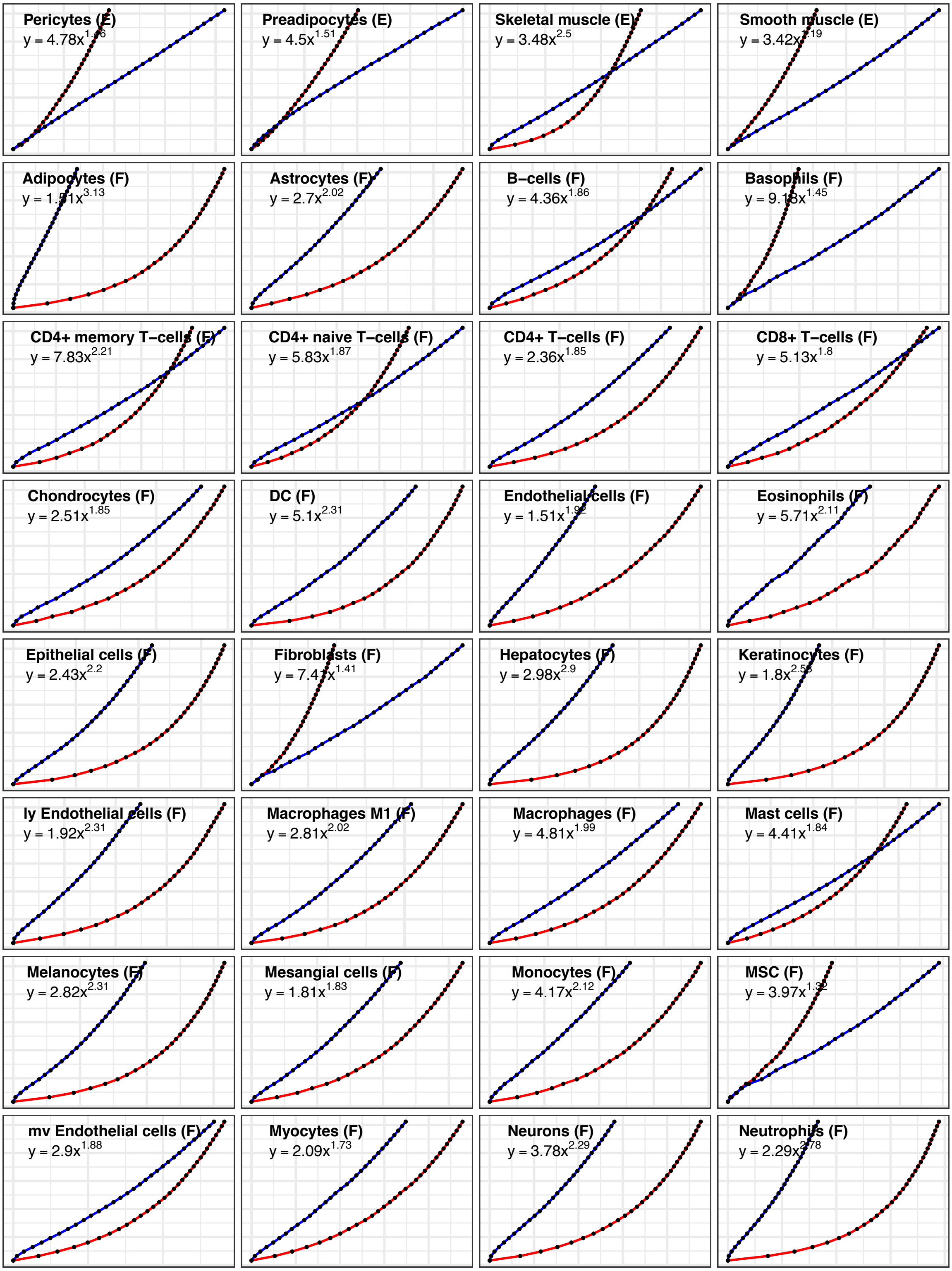

Pericytes (E)
y = 4.78x^1.46
Preadipocytes (E)
y = 4.5x^1.51
Skeletal muscle (E)
y = 3.48x^2.5
Smooth muscle (E)
y = 3.42x^1.19
Adipocytes (F)
y = 1.51x^3.13
Astrocytes (F)
y = 2.7x^2.02
B−cells (F)
y = 4.36x^1.86
Basophils (F)
y = 9.18x^1.45
CD4+ memory T−cells (F)
y = 7.83x^2.21
CD4+ naive T−cells (F)
y = 5.83x^1.87
CD4+ T−cells (F)
y = 2.36x^1.85
CD8+ T−cells (F)
y = 5.13x^1.8
Chondrocytes (F)
y = 2.51x^1.85
DC (F)
y = 5.1x^2.31
Endothelial cells (F)
y = 1.51x^1.92
Eosinophils (F)
y = 5.71x^2.11
Epithelial cells (F)
y = 2.43x^2.2
Fibroblasts (F)
y = 7.41x^1.41
Hepatocytes (F)
y = 2.98x^2.9
Keratinocytes (F)
y = 1.8x^2.58
ly Endothelial cells (F)
y = 1.92x^2.31
Macrophages M1 (F)
y = 2.81x^2.02
Macrophages (F)
y = 4.81x^1.99
Mast cells (F)
y = 4.41x^1.84
Melanocytes (F)
y = 2.82x^2.31
Mesangial cells (F)
y = 1.81x^1.83
Monocytes (F)
y = 4.17x^2.12
MSC (F)
y = 3.97x^1.32
mv Endothelial cells (F)
y = 2.9x^1.88
Myocytes (F)
y = 2.09x^1.73
Neurons (F)
y = 3.78x^2.29
Neutrophils (F)
y = 2.29x^2.78

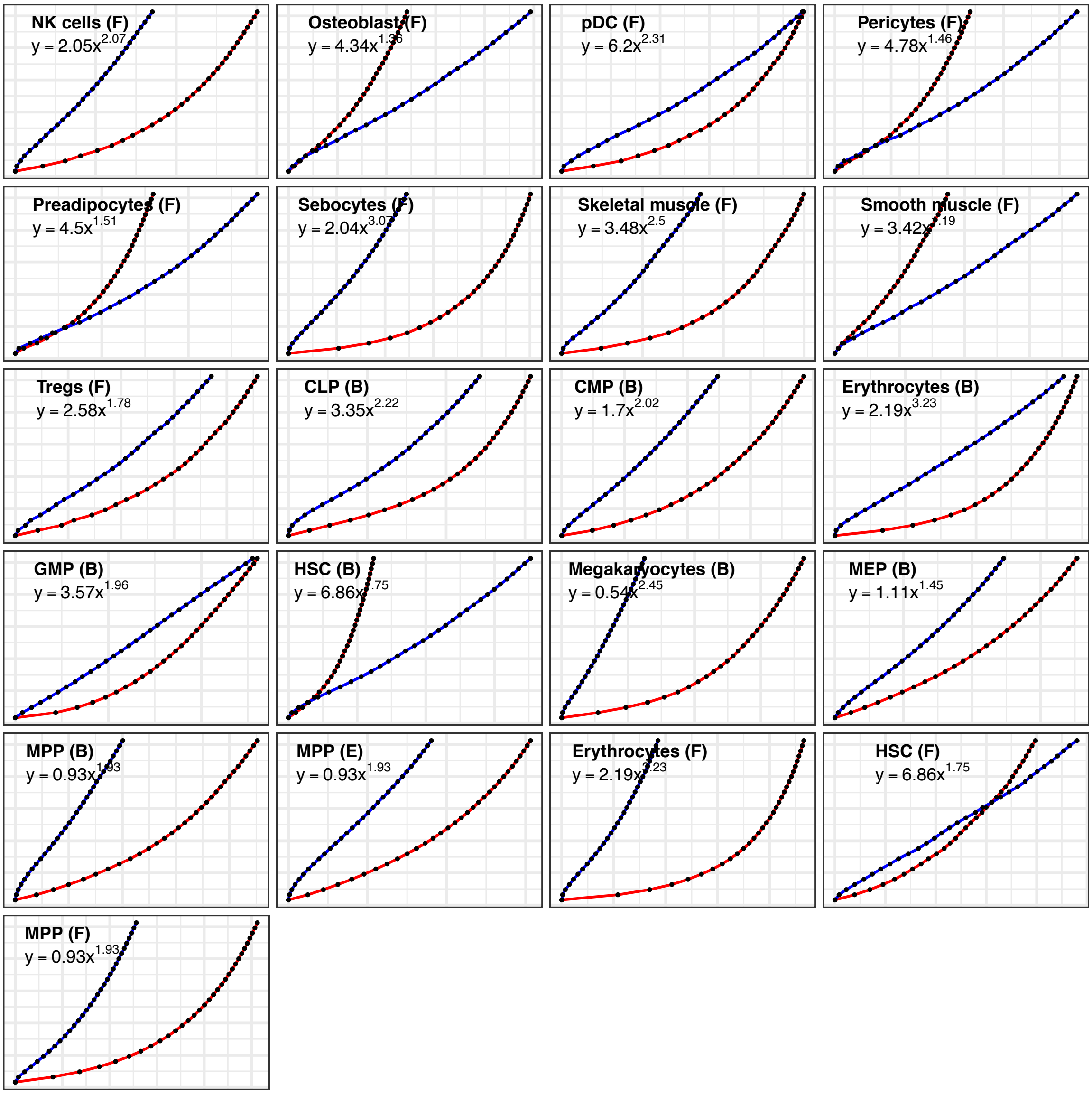

Figure S4. Transformation procedure of raw scores to linear scales. For each cell type we generated 126 simulated mixtures using 2 cell types – the corresponding cell type and one of two options – MPP (multipotent progenitor) cells or Endothelial cells. We used those cell types because they are found in all three sequencing-based methods (this procedure was performed for both seq-based and array-based samples, but show here only transformations of seq-based). Endothelial cells was used for the hematopoietic stem cells, and MPP for all other cell types. In these synthetic simulations the expression profile of the cell types that is used is the median gene expression across samples of the corresponding cell type. To fit a power function, we used only the simulations were the corresponding cell types abundances are between 0.8% to 25.6%. We used this range because we are mostly interested in identifying cell types with low abundance, and above that the function exponential increase may interfere in a precise fitting. The raw scores were shifted to zero (by deducting the score of 0.08%) and divided by 5000. Each plot shows the fitted curve (in red, the black dots are the data points) and the curve after transforming it using the learned formula (if the cell type is available in multiple sources, the parameters are averaged). The learned formula is presented at the top.

Supplementary Figure 5:

Cell types inferences in gene expression simulations using training samples

Blueprint simulation 1 (using training samples)

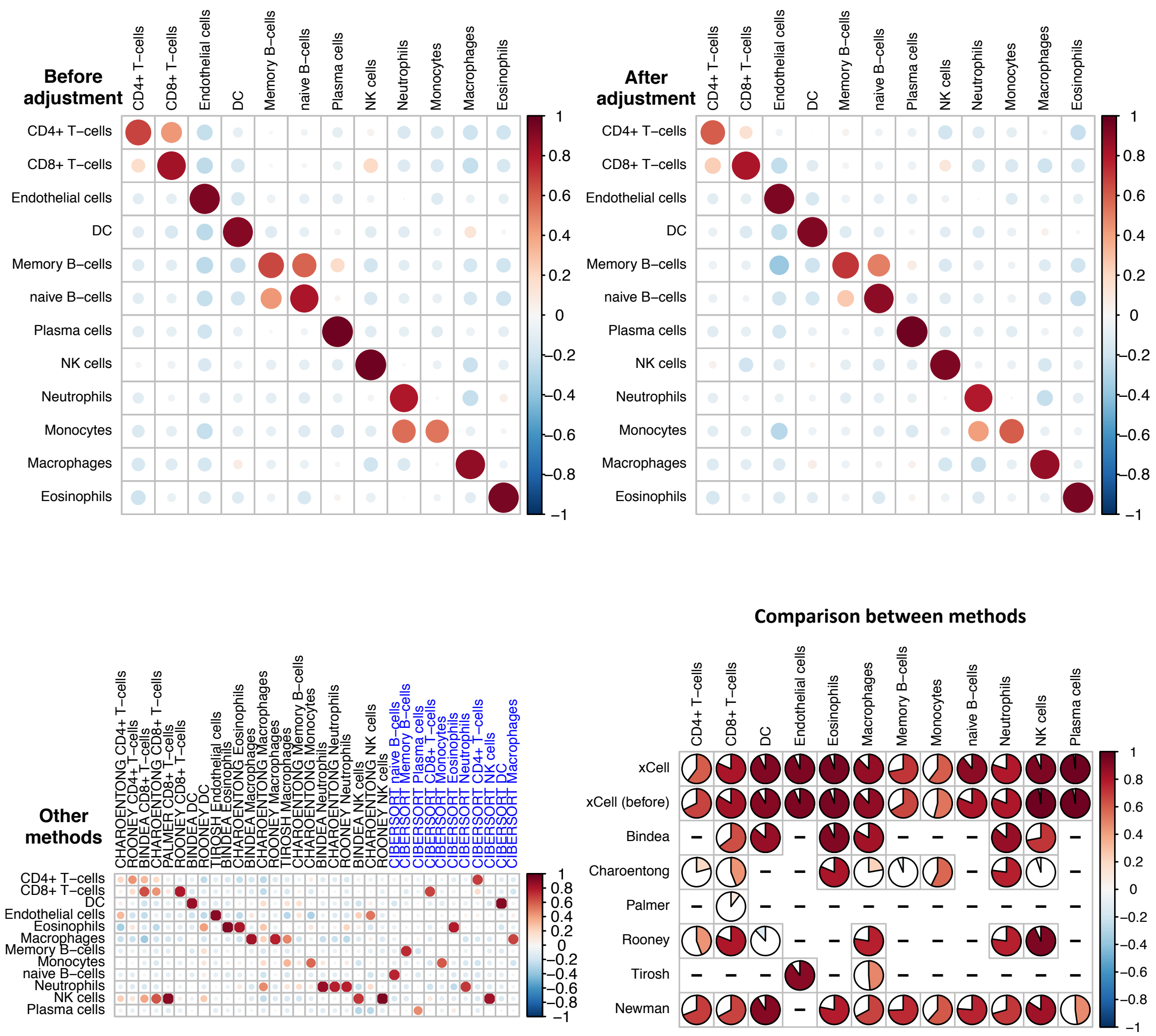

Blueprint simulation 2 (using training samples)

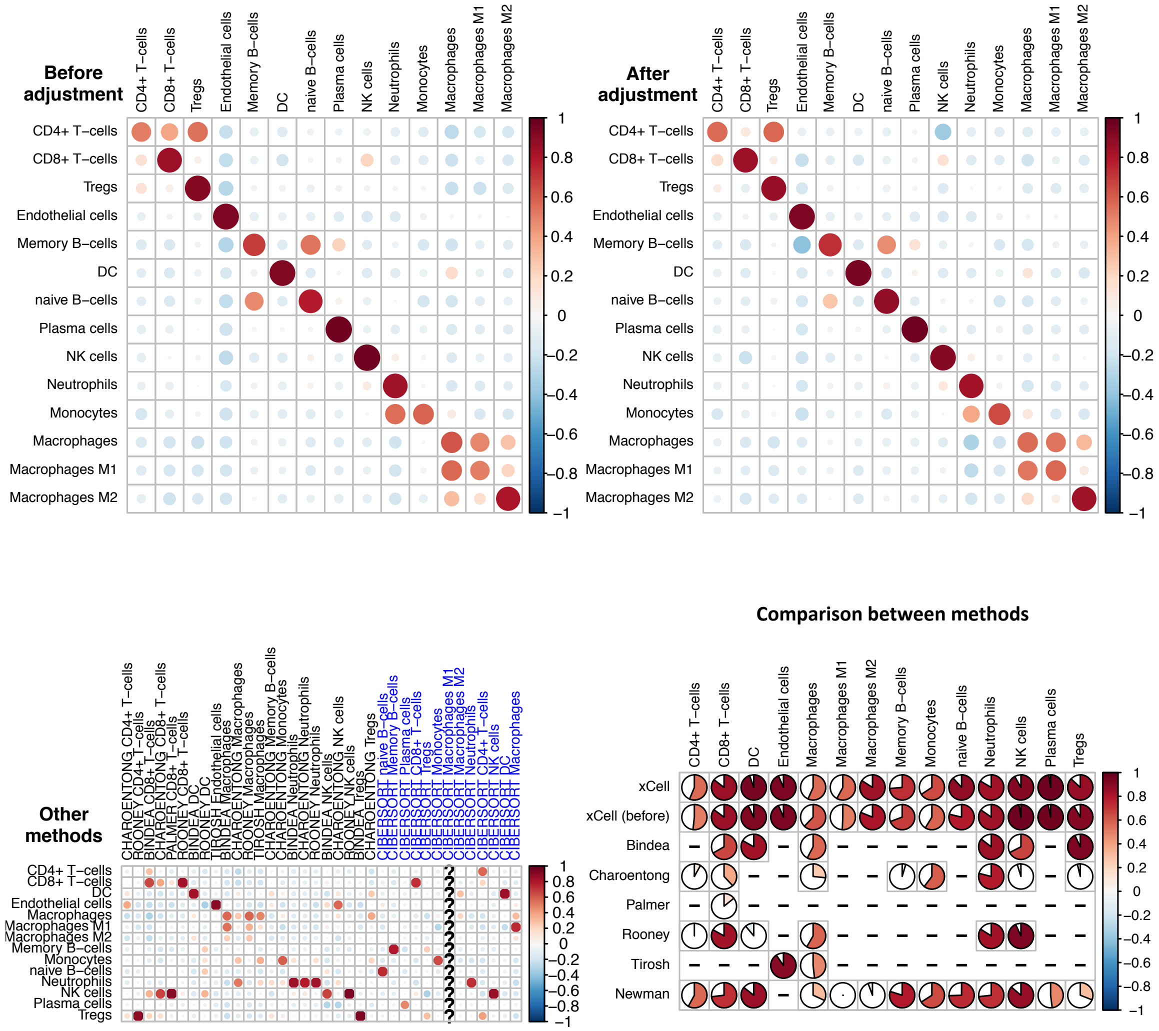

Blueprint simulation 3 (using training samples)

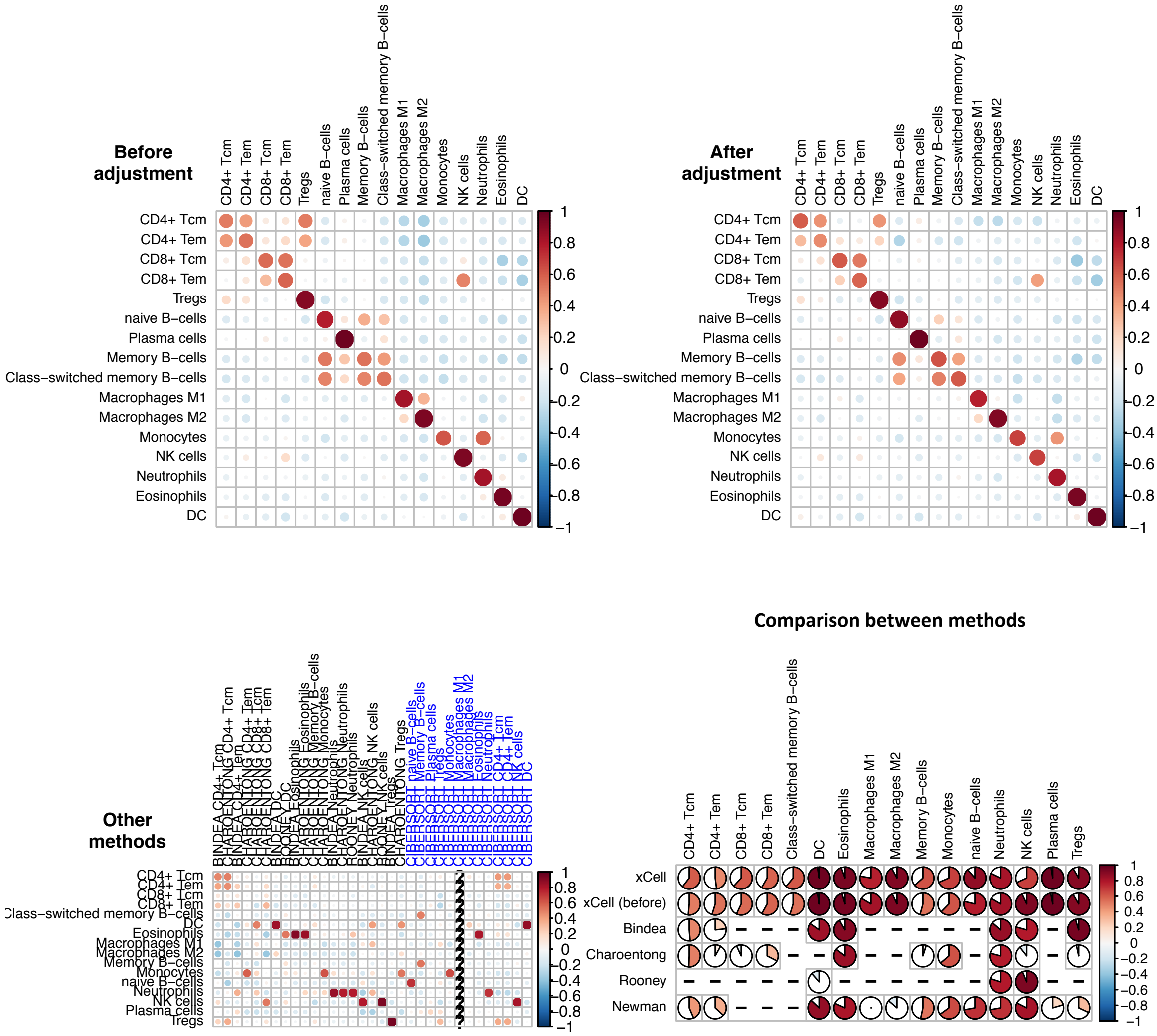

ENCODE simulation (using training samples)

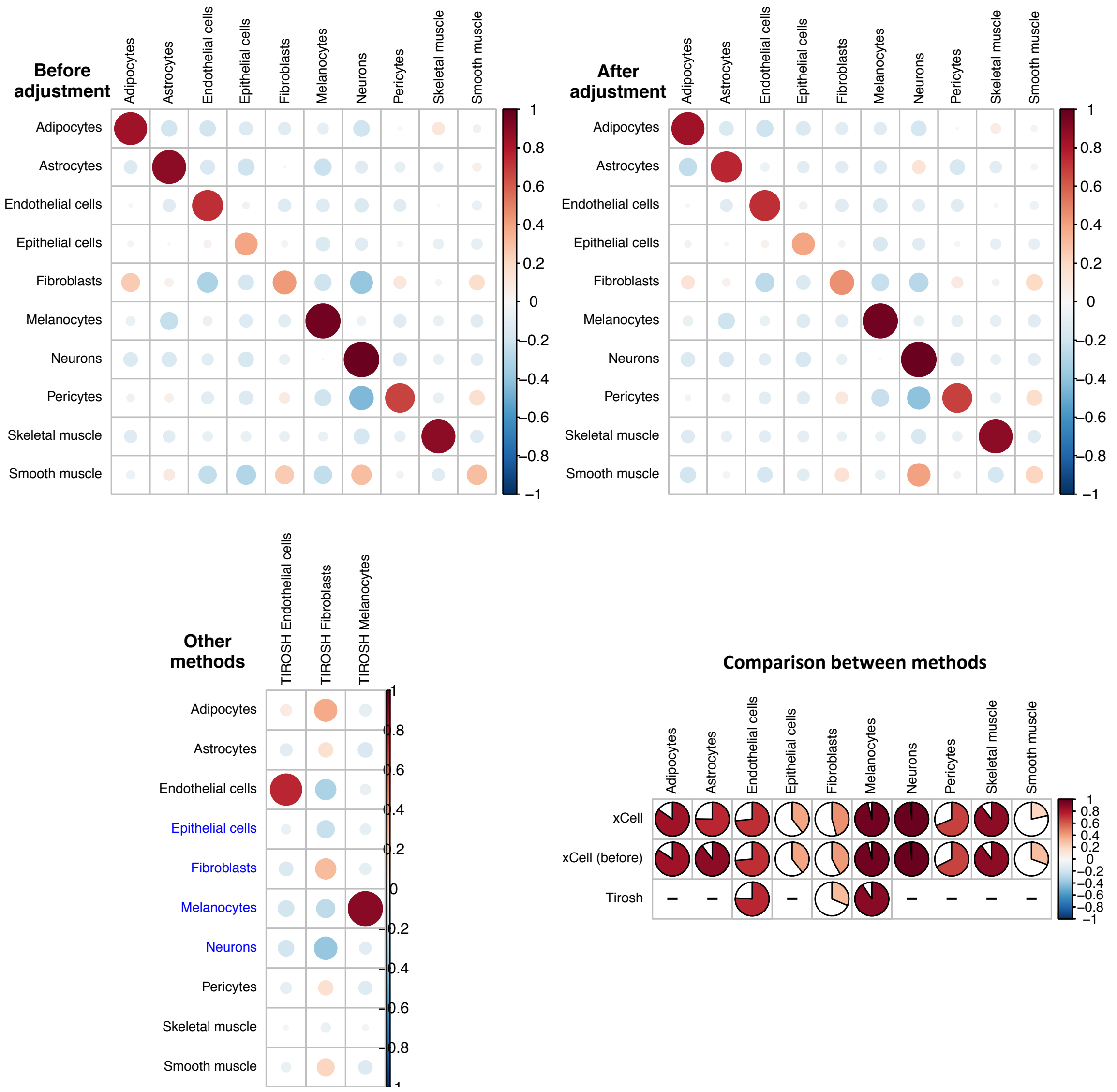

FANTOM5 simulation 1 (using training samples)

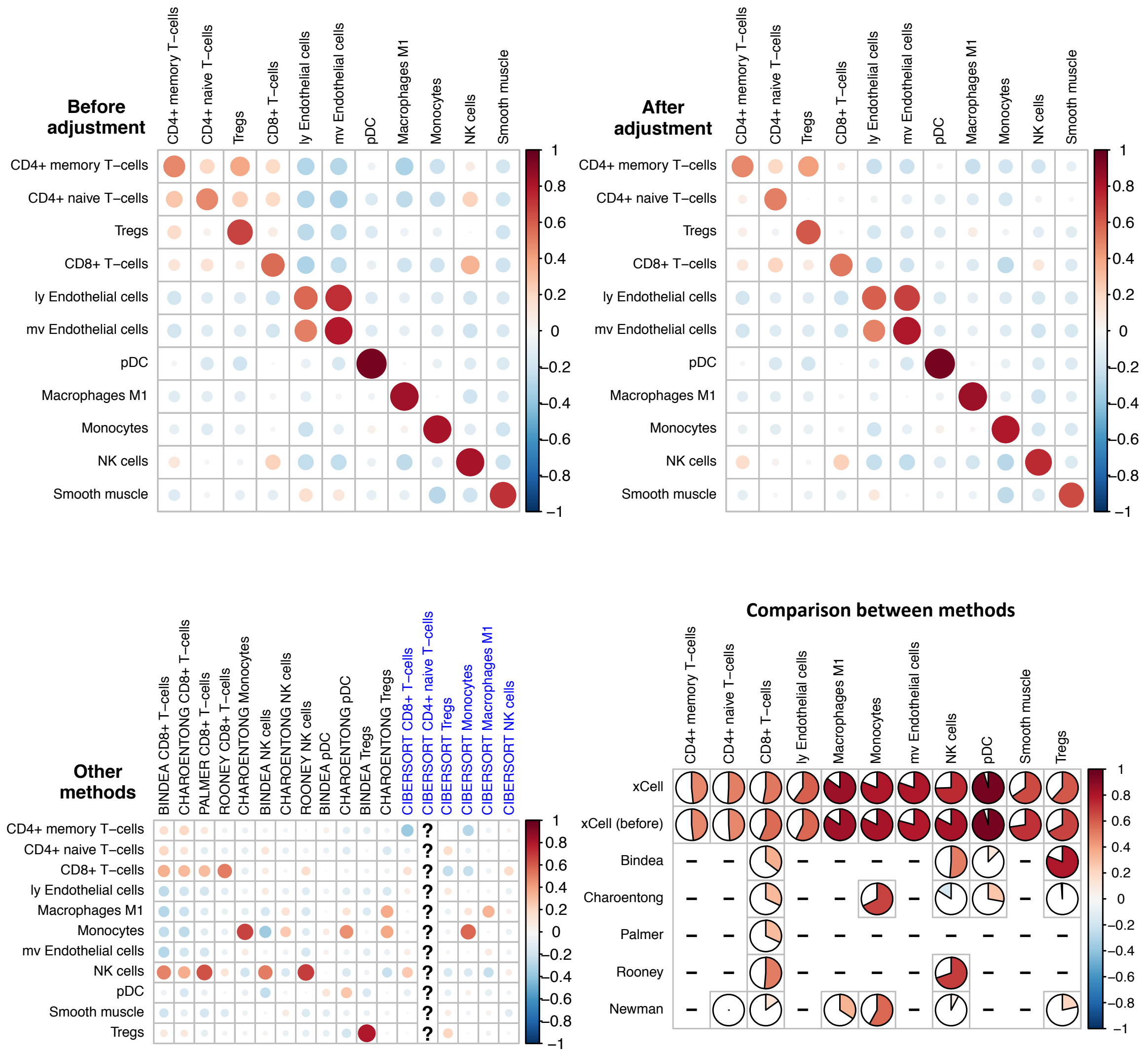

FANTOM5 simulation 2 (using training samples)

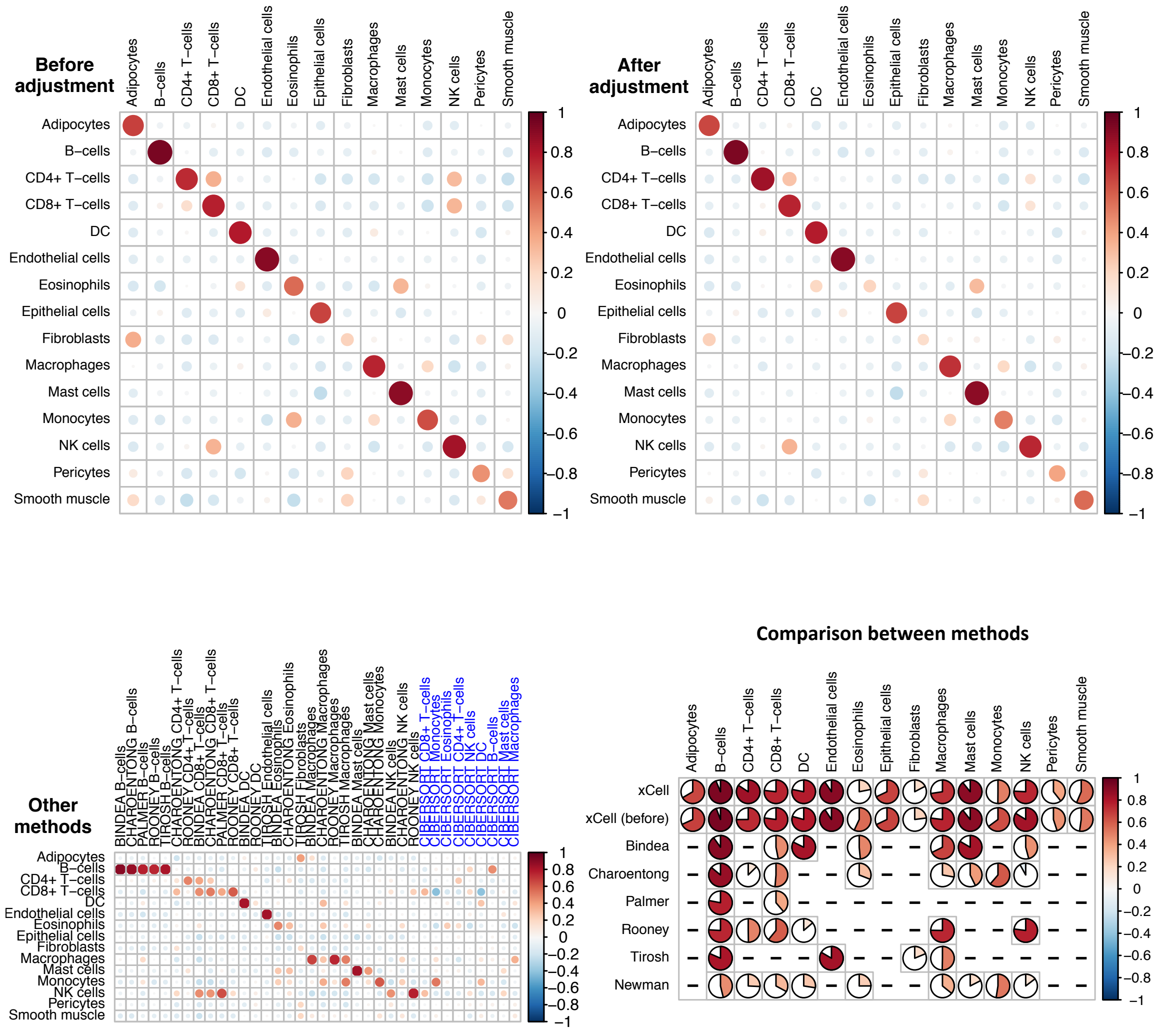

IRIS simulation (using training samples)

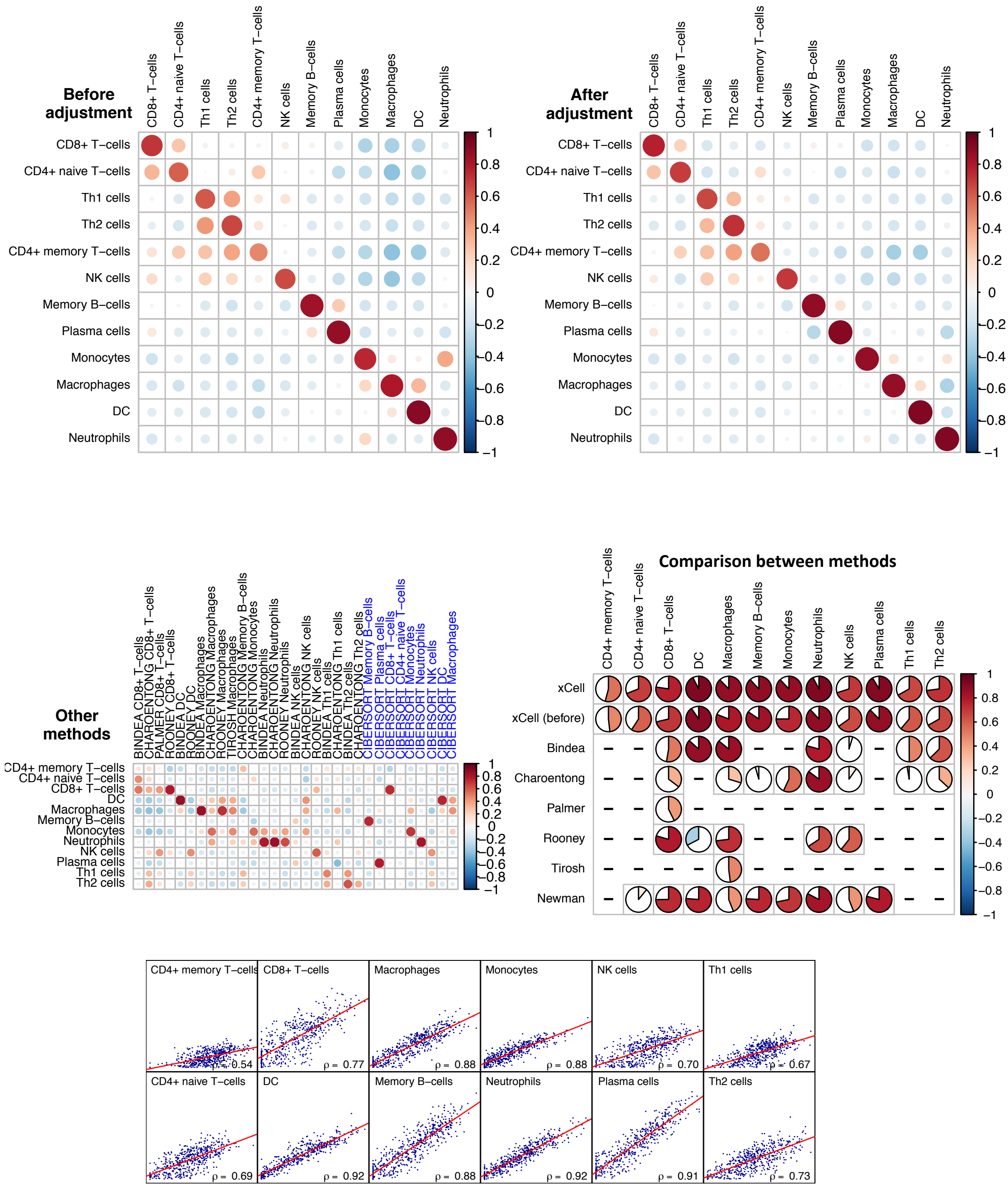

Novershtern simulation (using training samples)

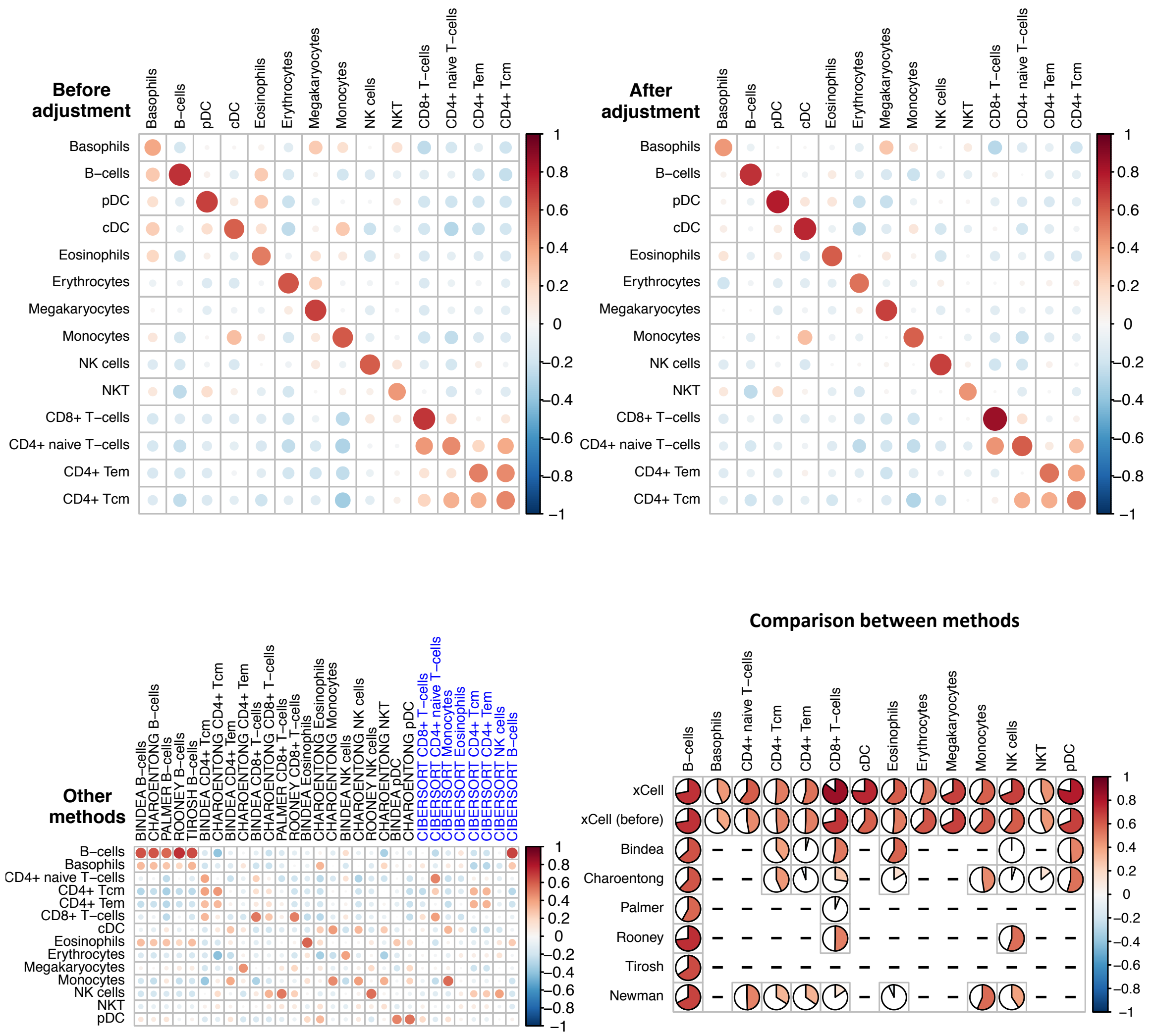

HPCA simulation 1 (using training samples)

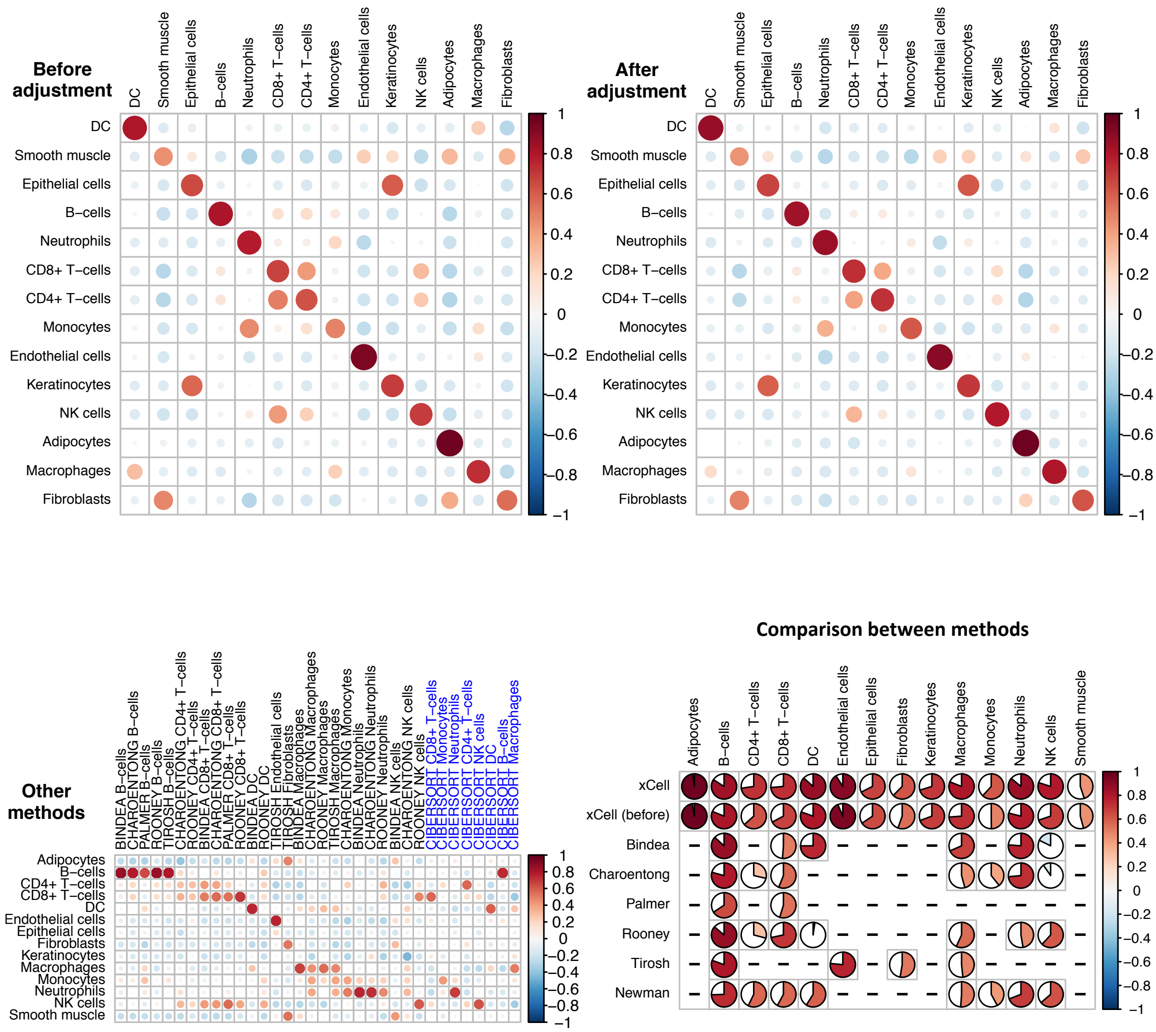

HPCA simulation 2 (using training samples)

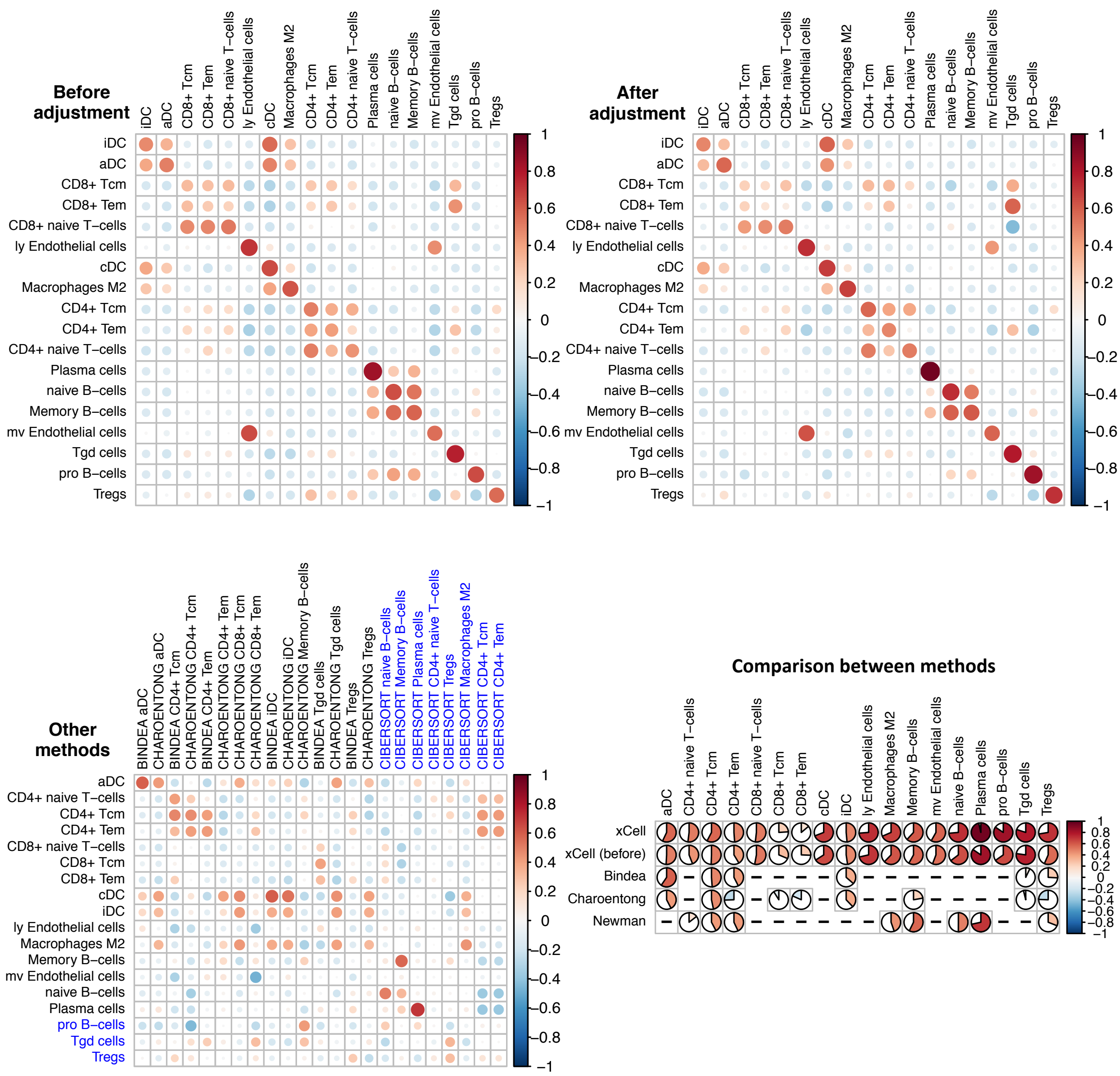

Summary table of simulation based on training samples

	Average R (diagonal)	Diagonal<br>Adjusted diagonal/ Non-adjusted	Adjusted diagonal/ Non-adjusted	Off-diagonal<br>Numbero of associations > 0.25	Adjusted diagonal/ Non-adjusted in >0.25 associations
**Blueprint 1**	0.836	1.006	0.892	4	0.709
**Blueprint 2**	0.797	1.019	0.899	9	0.837
**Blueprint 3**	0.757	1.005	0.840	16	0.803
**ENCODE**	0.693	0.973	0.940	3	0.883
**FANTOM5 1**	0.686	0.974	0.820	5	0.835
**FANTOM5 2**	0.654	0.939	0.788	7	0.693
**IRIS**	0.792	1.082	0.841	12	0.821
**Novershtern**	0.627	1.089	0.760	11	0.823
**HPCA 1**	0.757	1.069	0.853	13	0.820
**HPCA 2**	0.607	1.085	0.842	38	0.865
**Average**	**0.721**	**1.024**	**0.847**		**0.809**

Figure S5. Cell types inferences in gene expression simulations using training samples. Each slide presents the results of xCell, published signatures and CIBERSORT, in predicting the underlying abundances of 500 simulated mixtures generated using the training samples. Each sample in a mixture set is generated by randomly choosing one of the multiple samples corresponding to each of the cell types including in the mixture. **Top left**: Pearson coefficients of xCell before applying the spillover compensation. **Top right**: Pearson coefficients of xCell after applying the spillover compensation. **Bottom left:** Pearson coefficients of published signatures, CIBERSORT. **Bottom right**: Comparison between all methods.

Supplementary Figure 6:

Cell types inferences in gene expression simulations using testing samples

HPCA test simulation 1

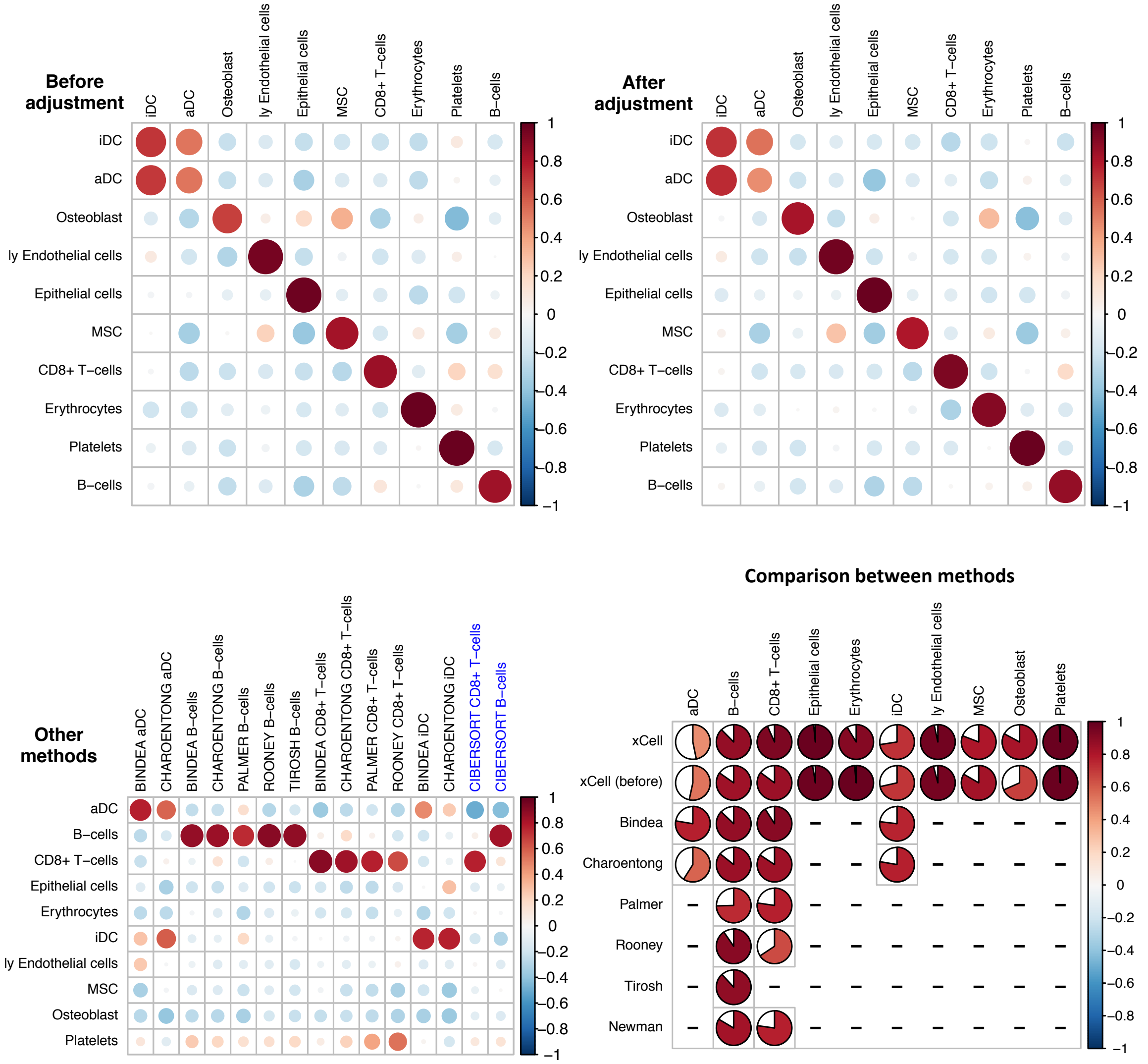

HPCA test simulation 2

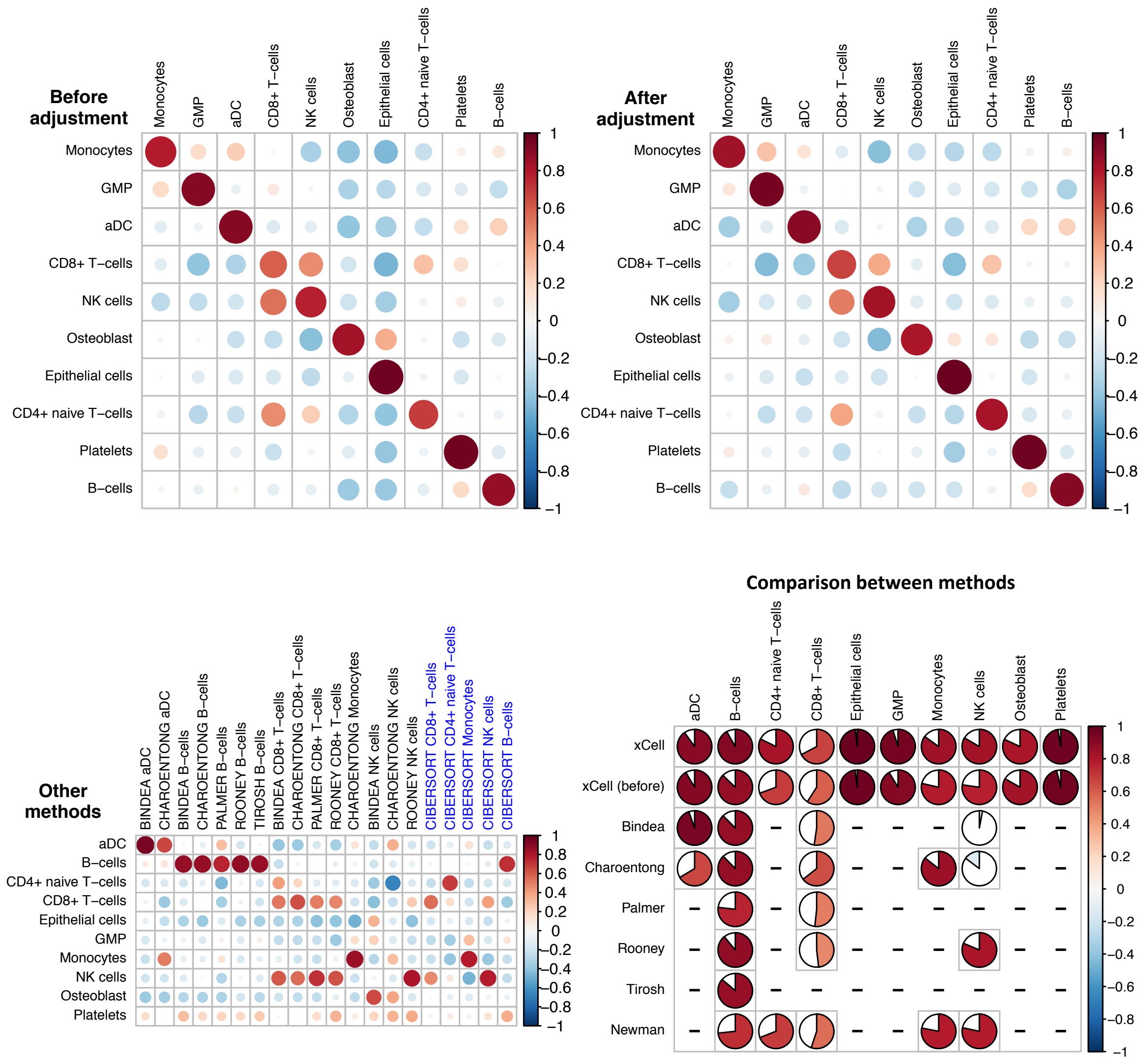

HPCA test simulation 3

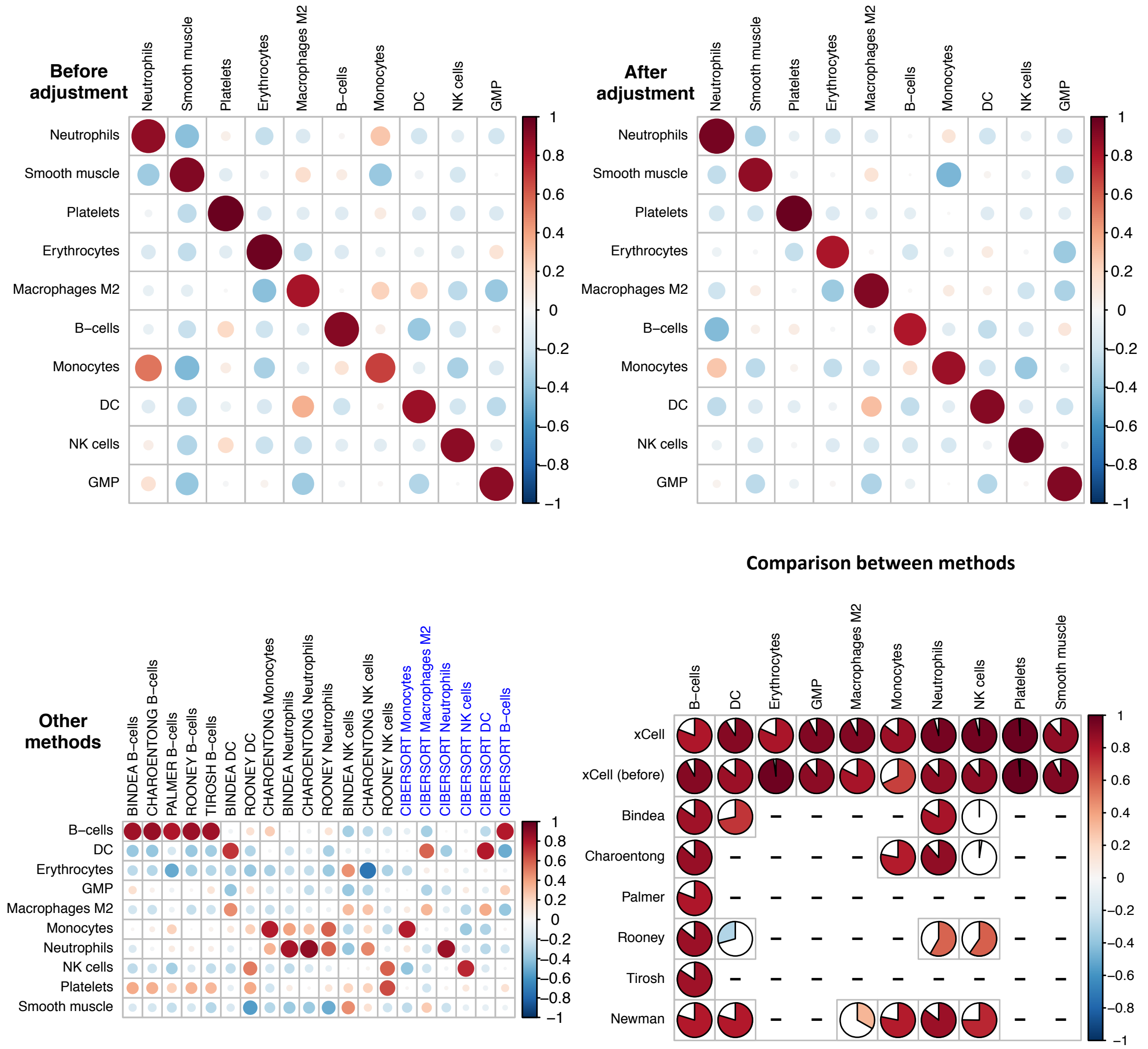

Novershtern test simulation 1

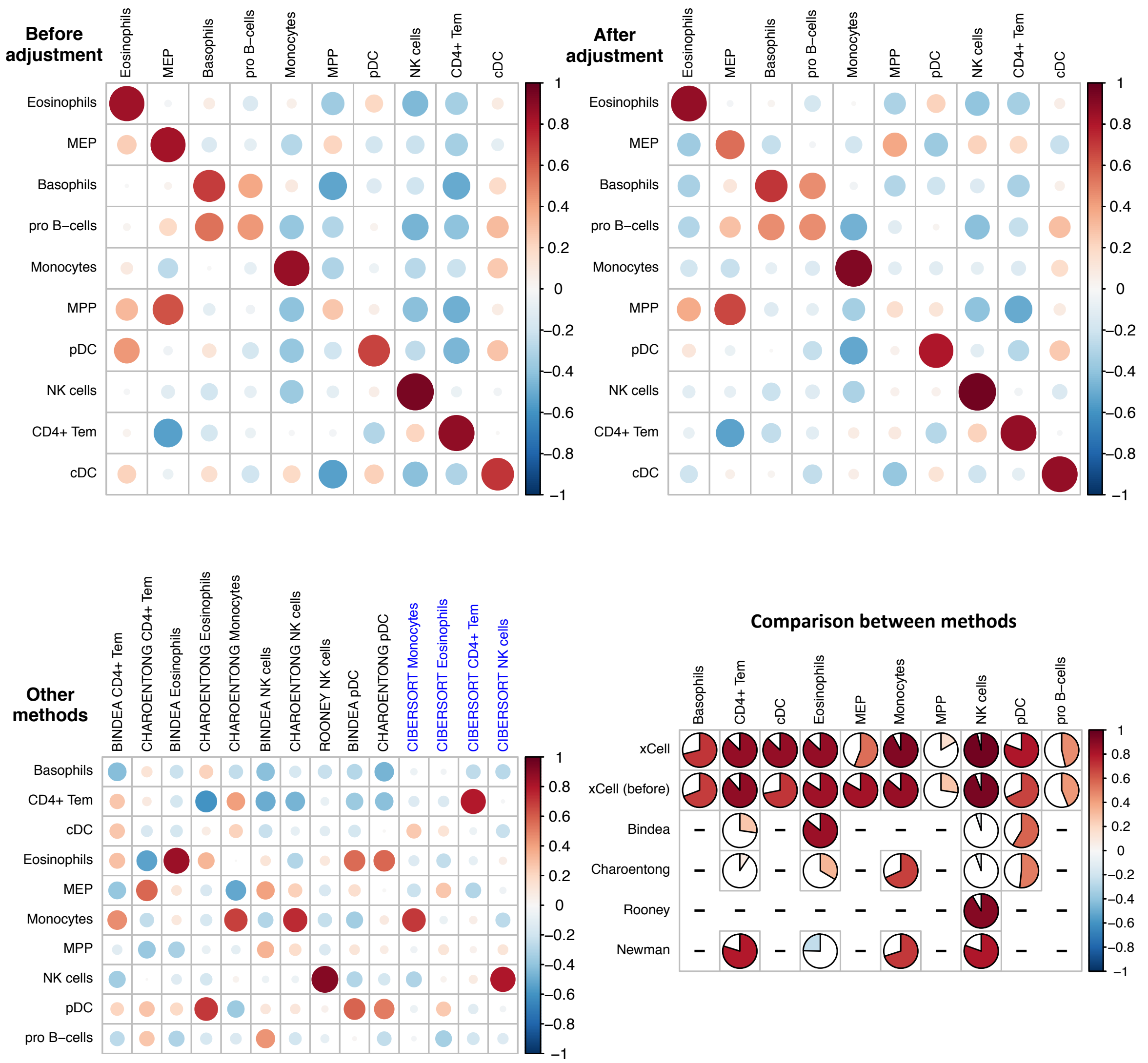

Novershtern test simulation 2

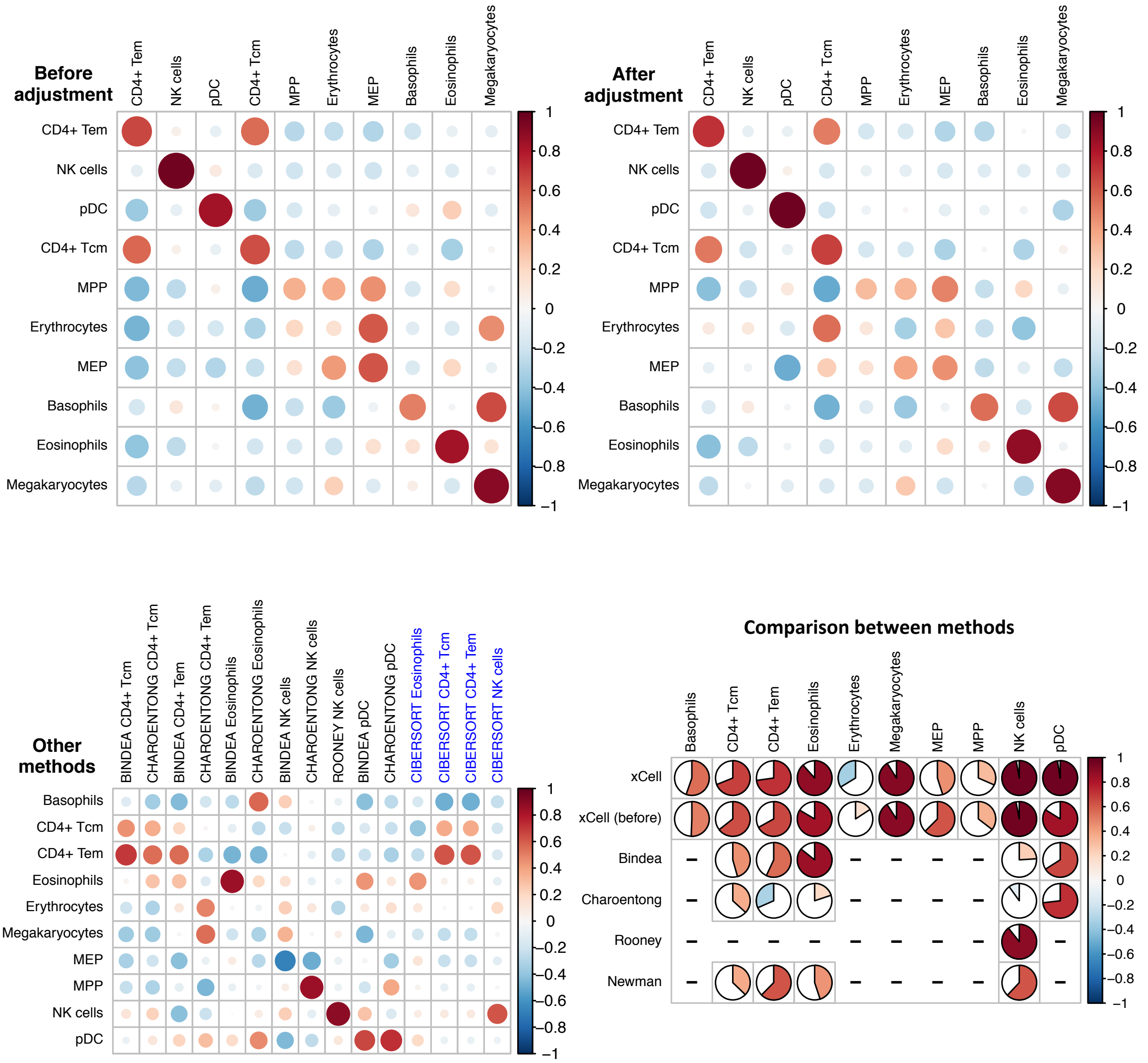

Novershtern test simulation 3

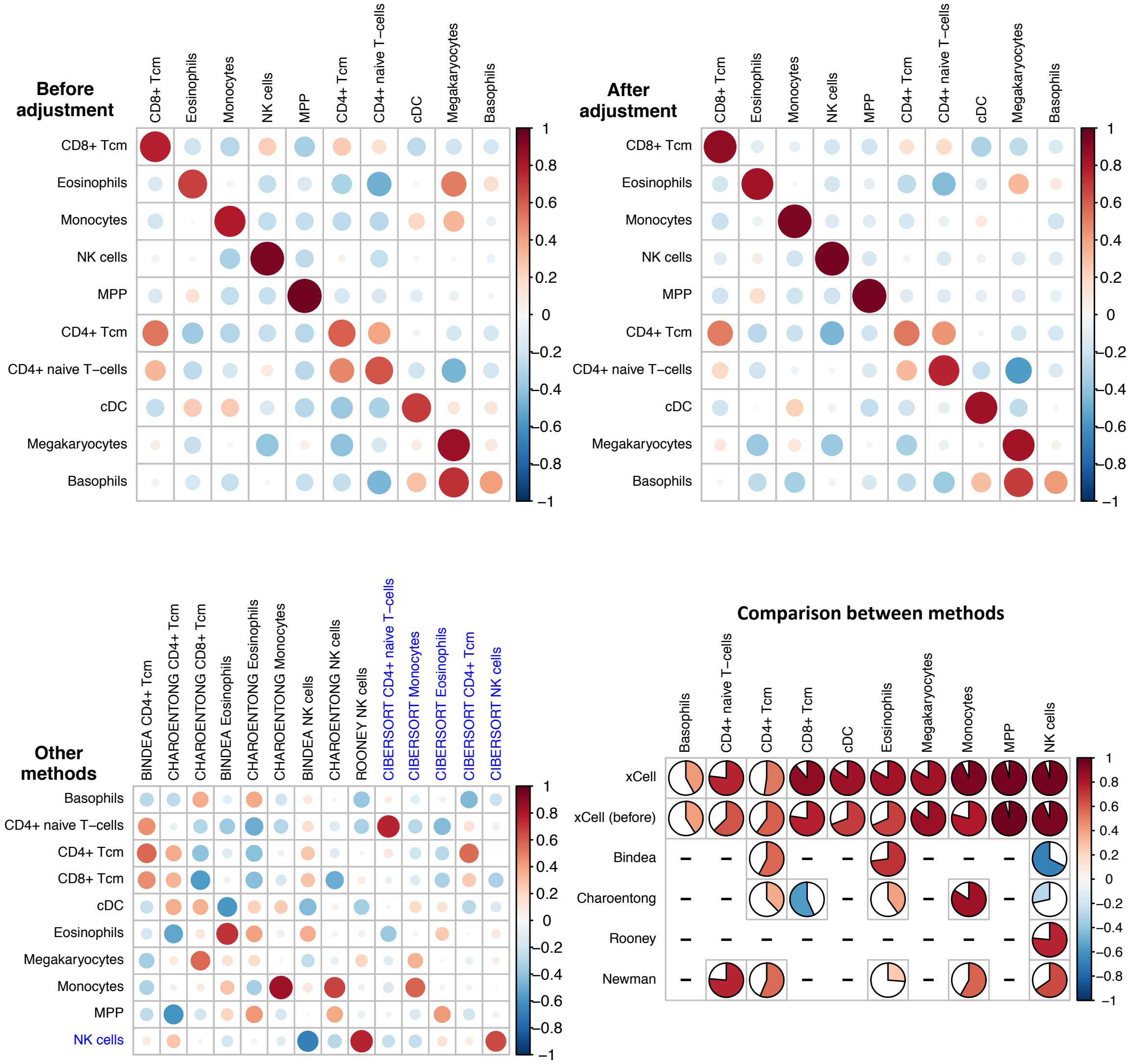

IRIS test simulation 1

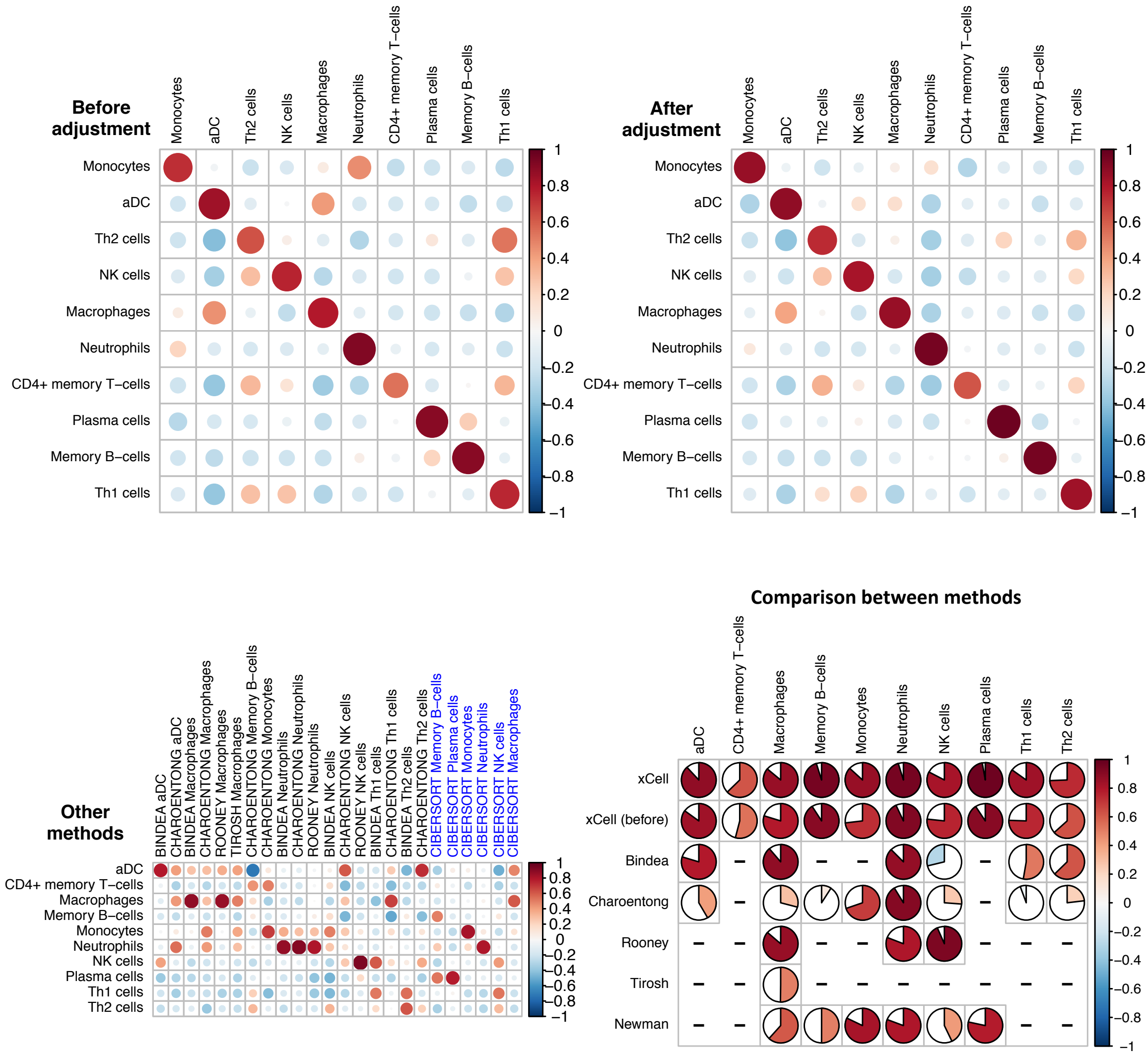

IRIS test simulation 2

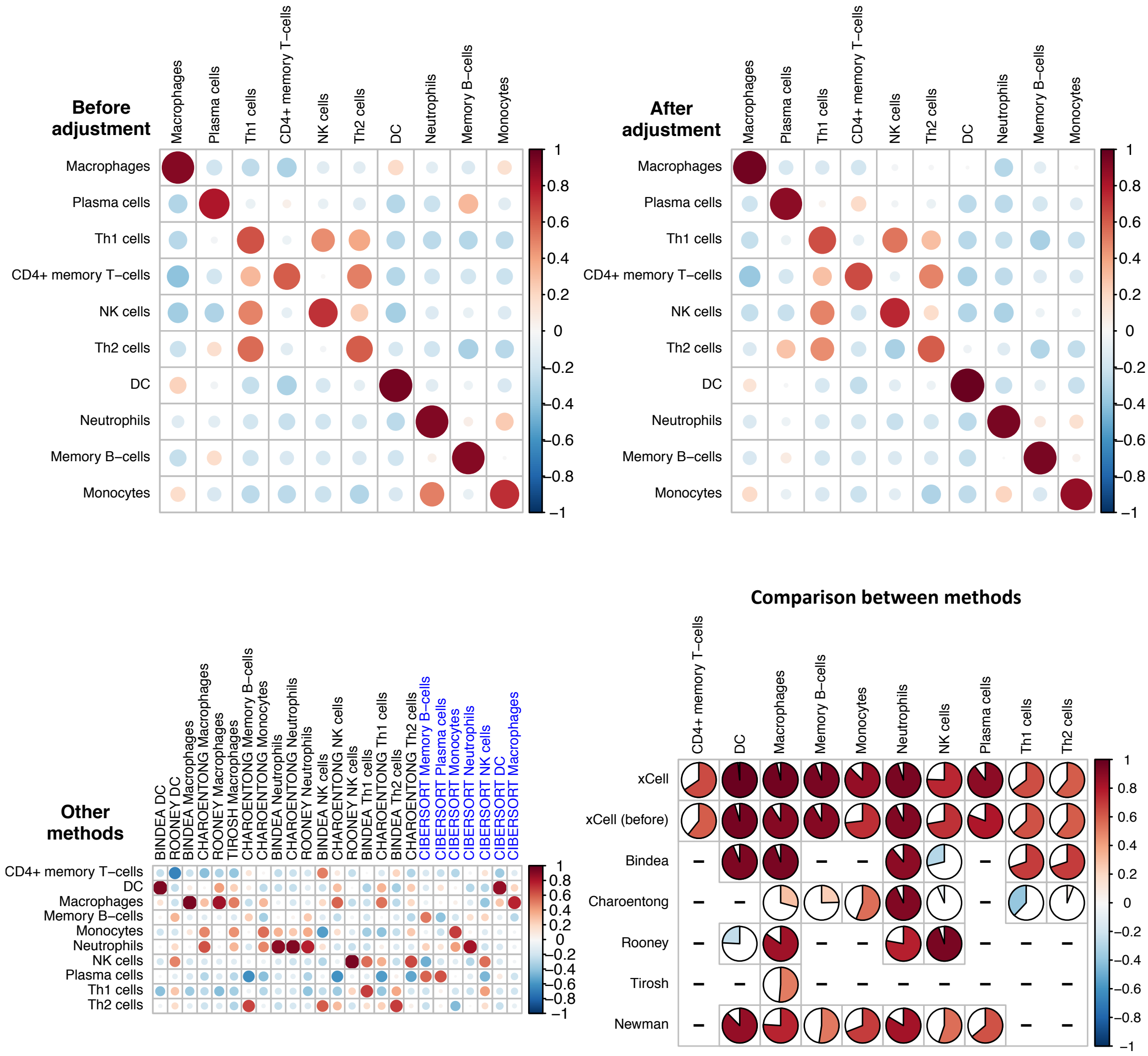

IRIS test simulation 3

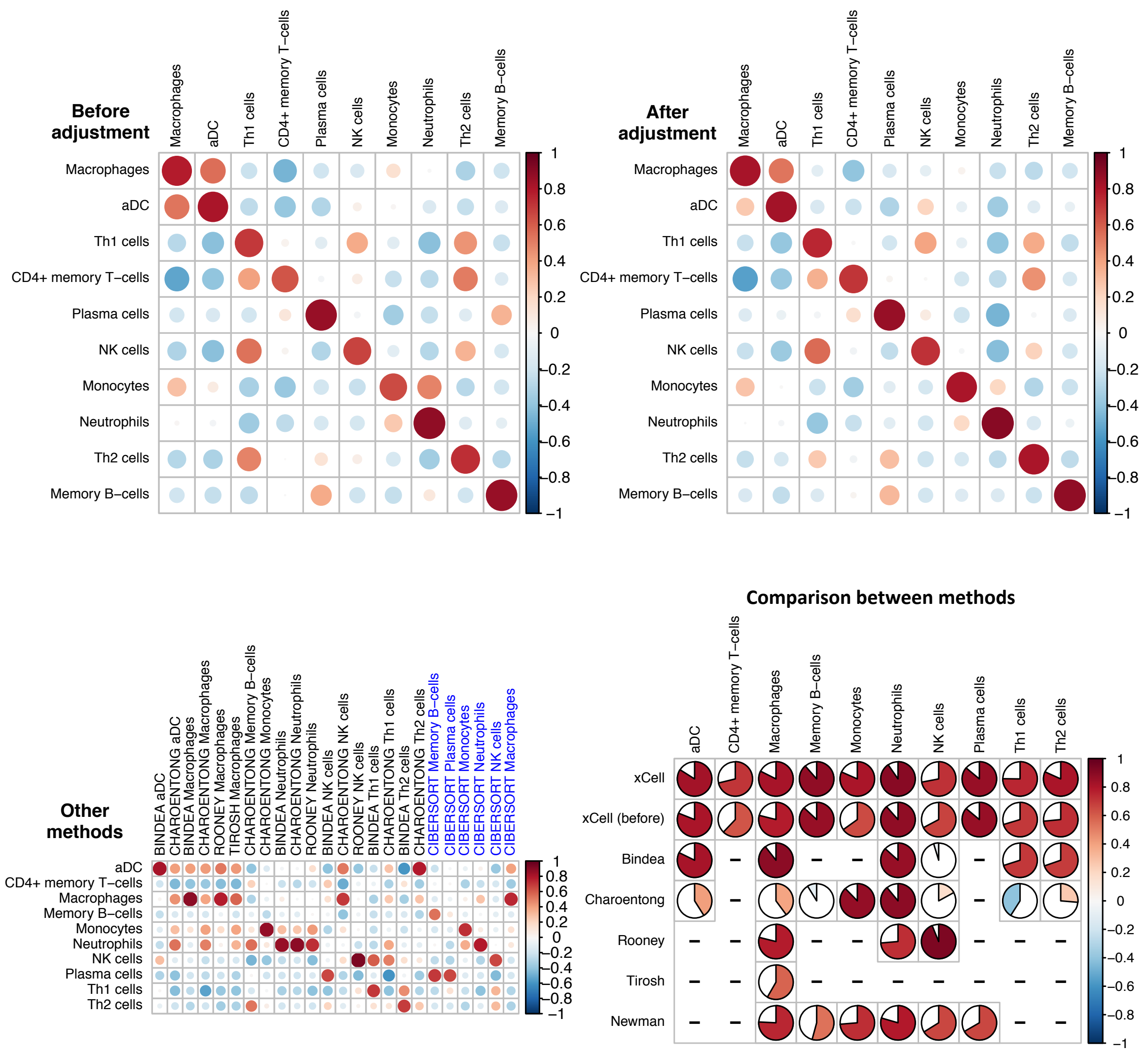

Blueprint test simulation 1

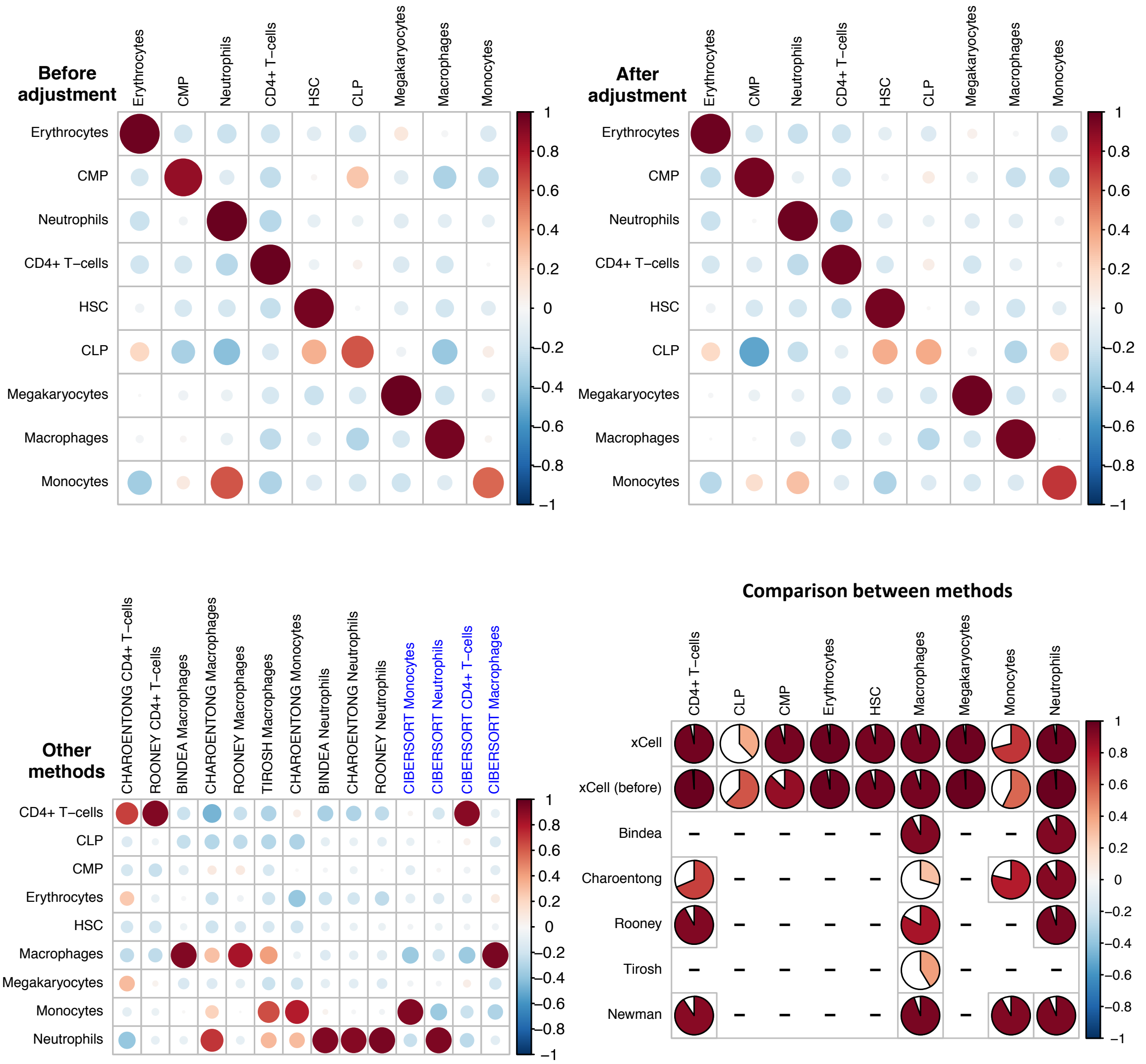

Blueprint test simulation 2

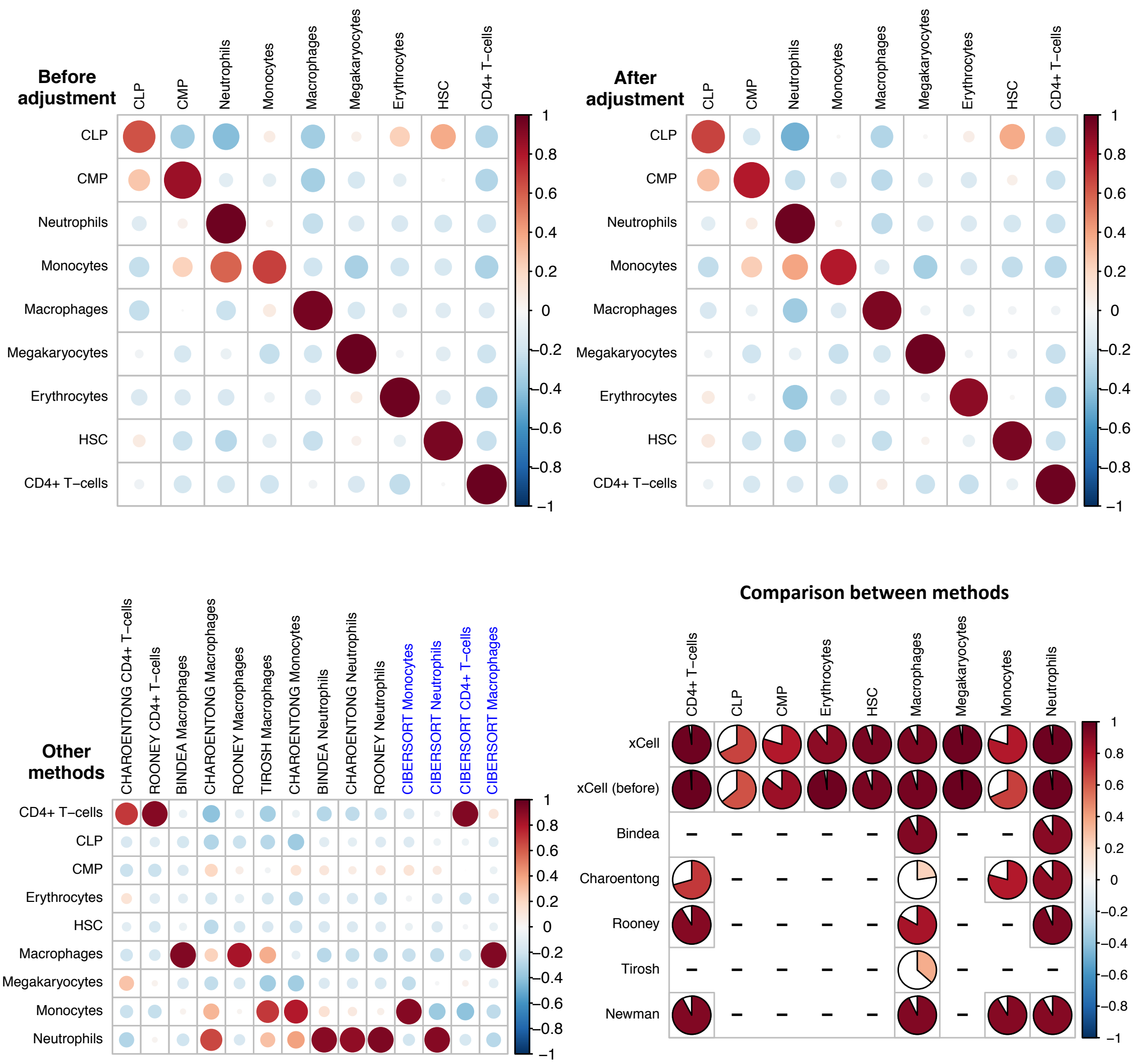

Blueprint test simulation 3

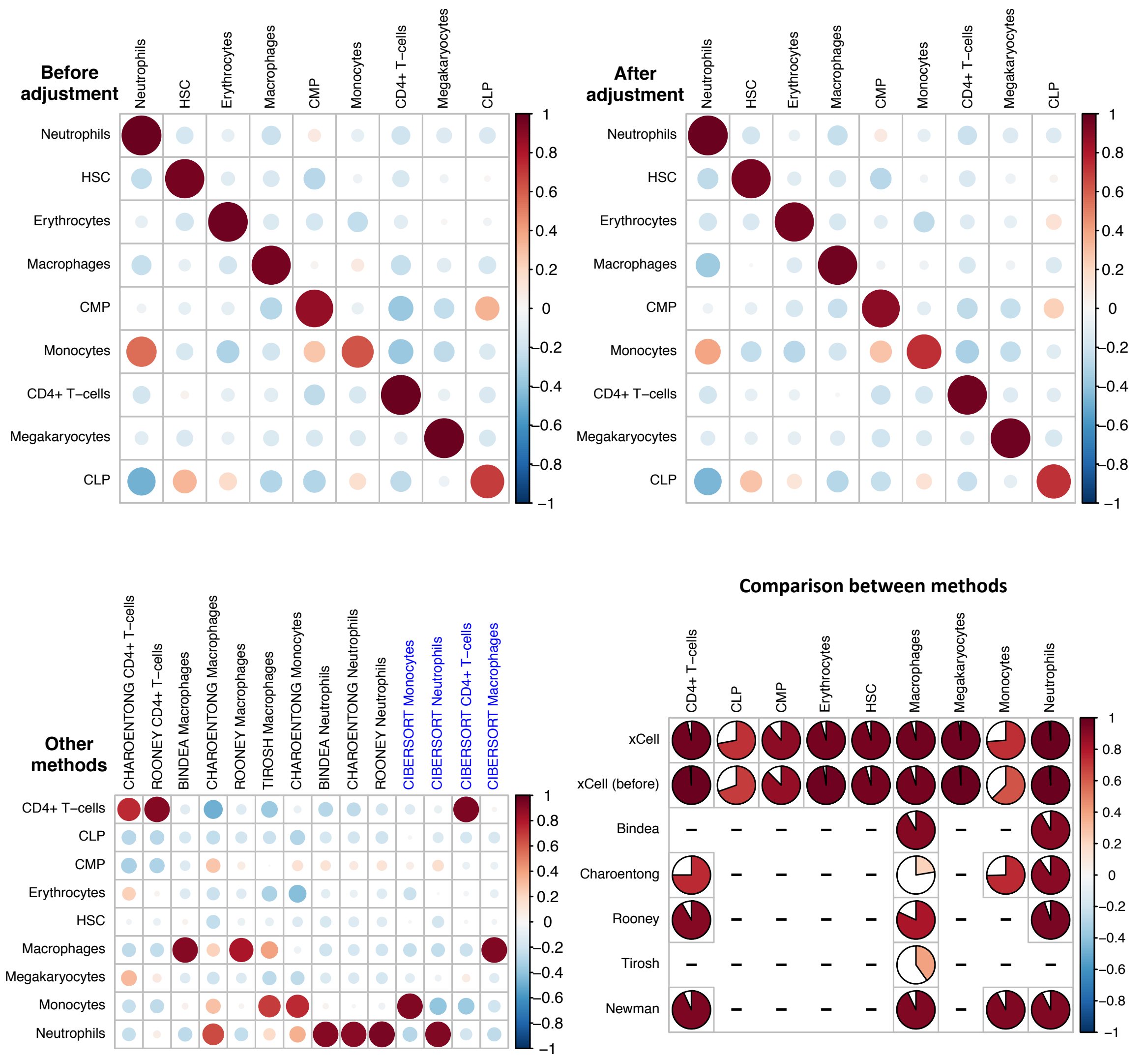

ENCODE test simulation 1

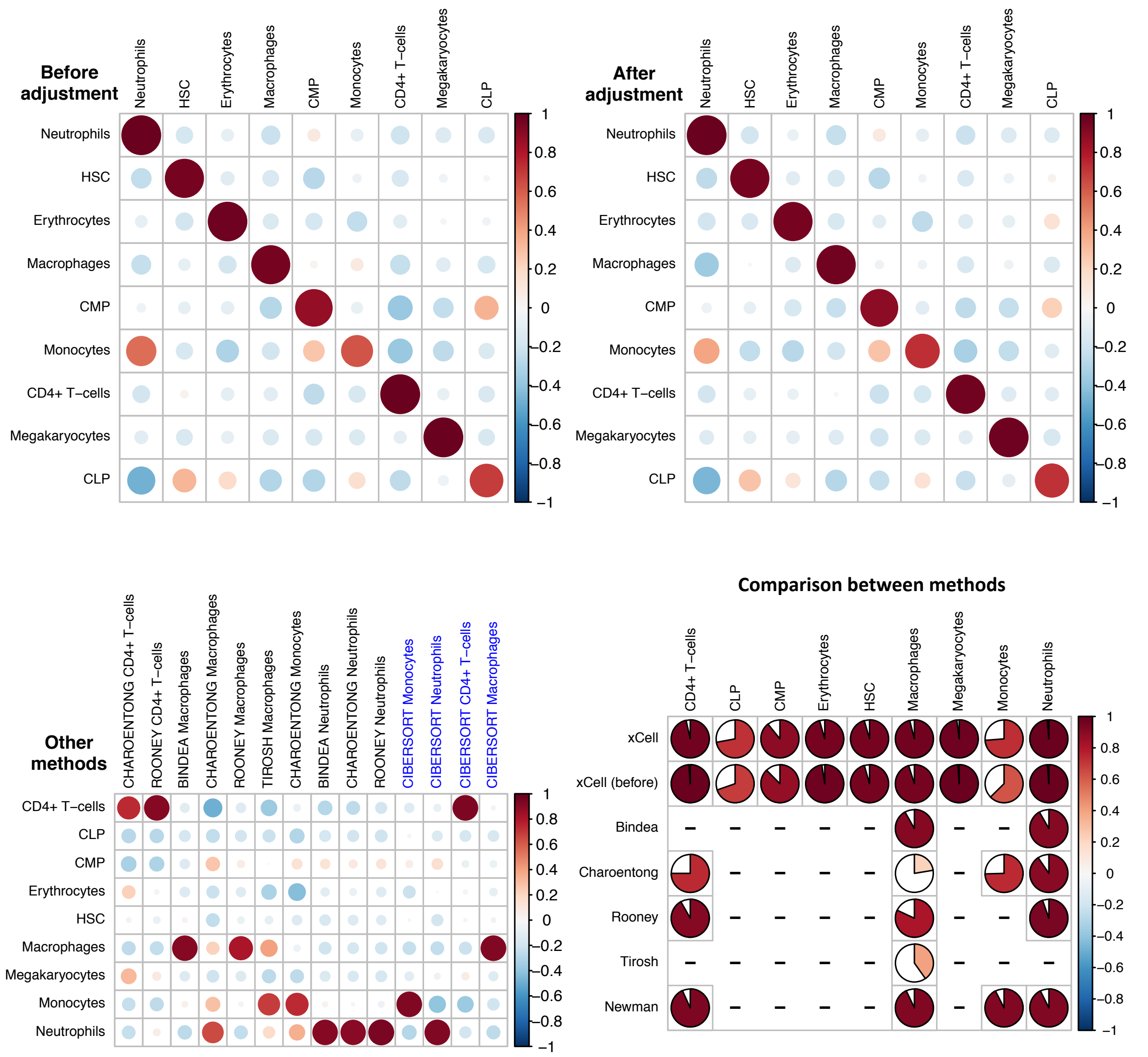

ENCODE test simulation 2

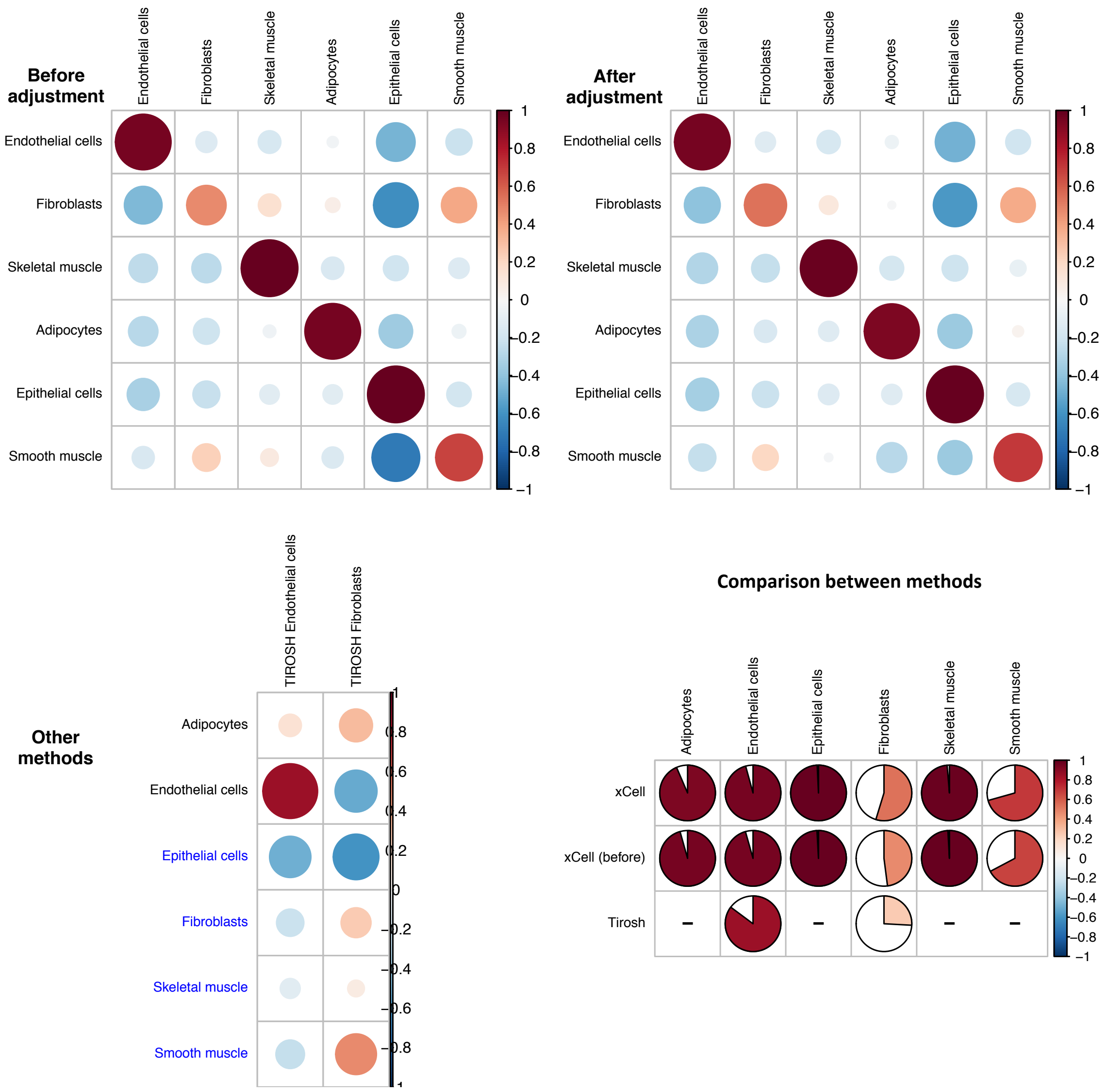

ENCODE test simulation 3

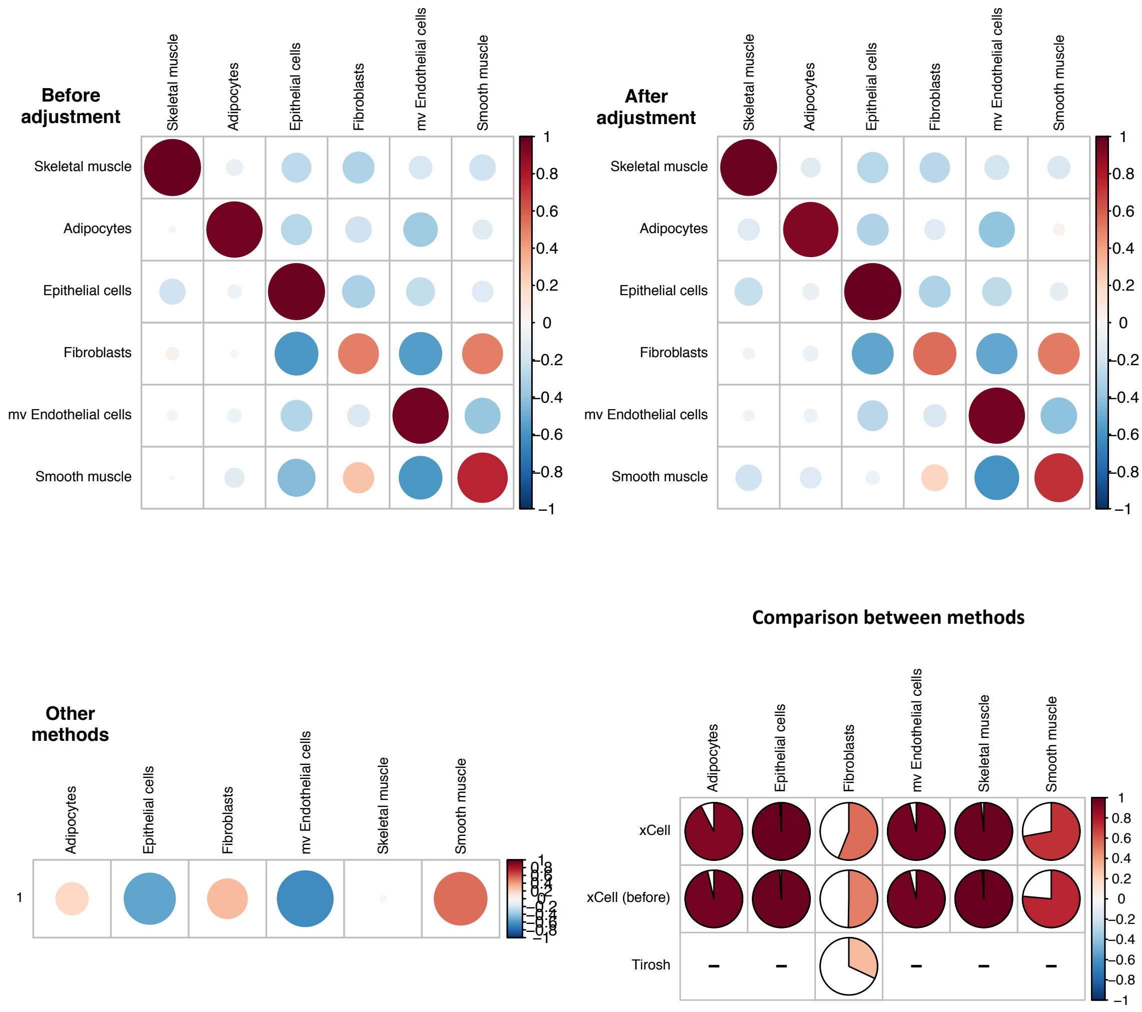

FANTOM5 test simulation 1

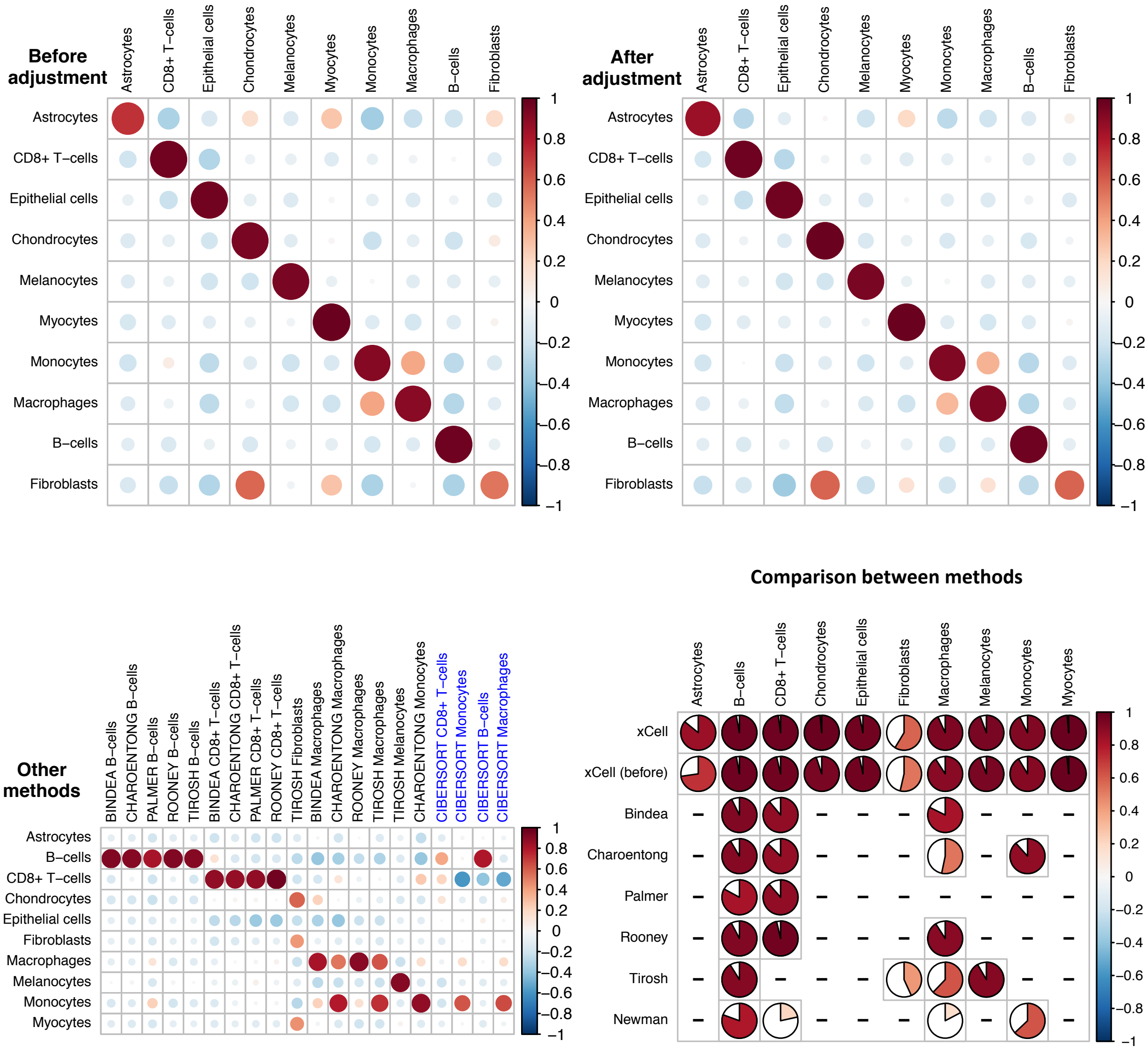

FANTOM5 test simulation 2

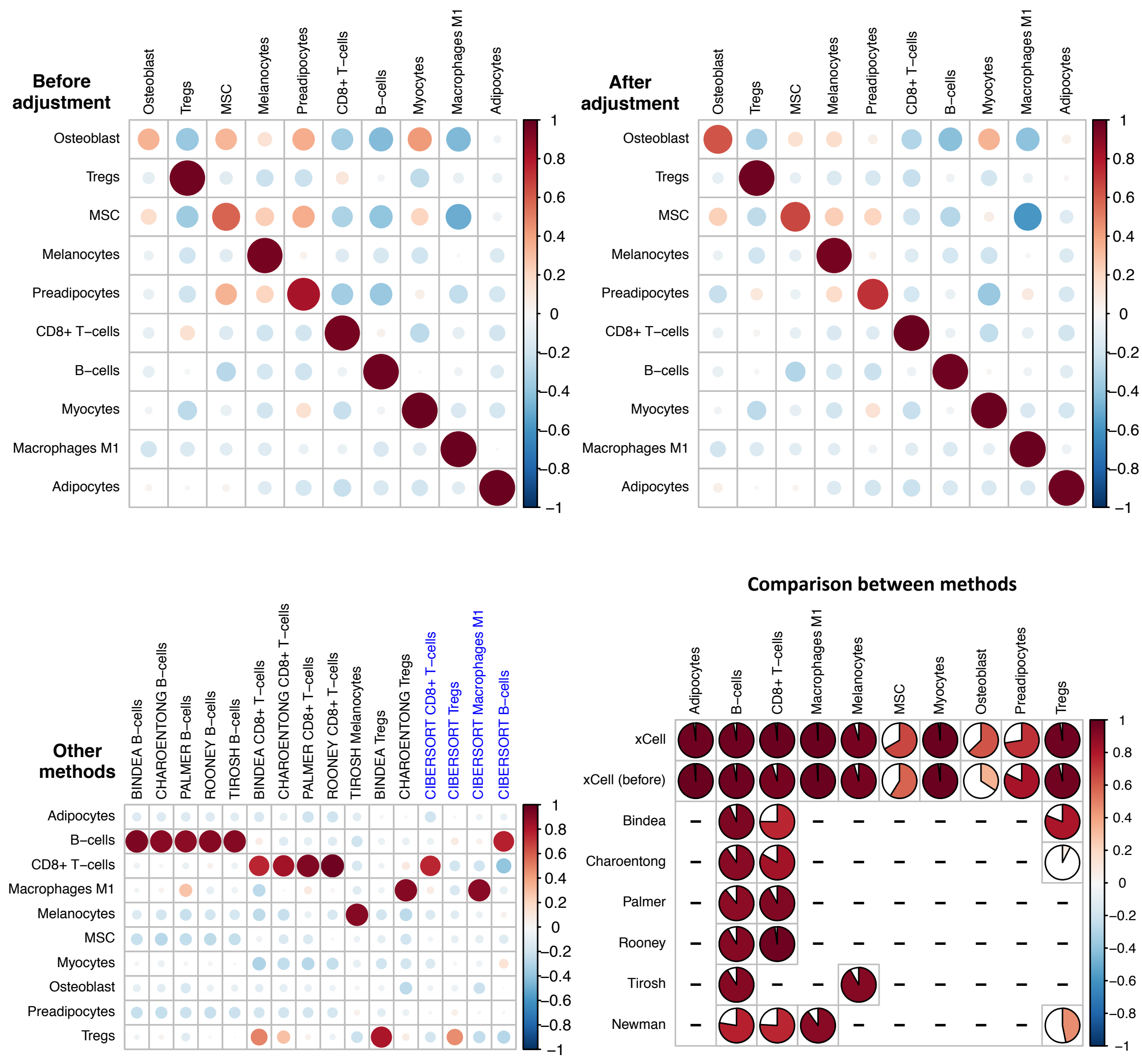

FANTOM5 test simulation 3

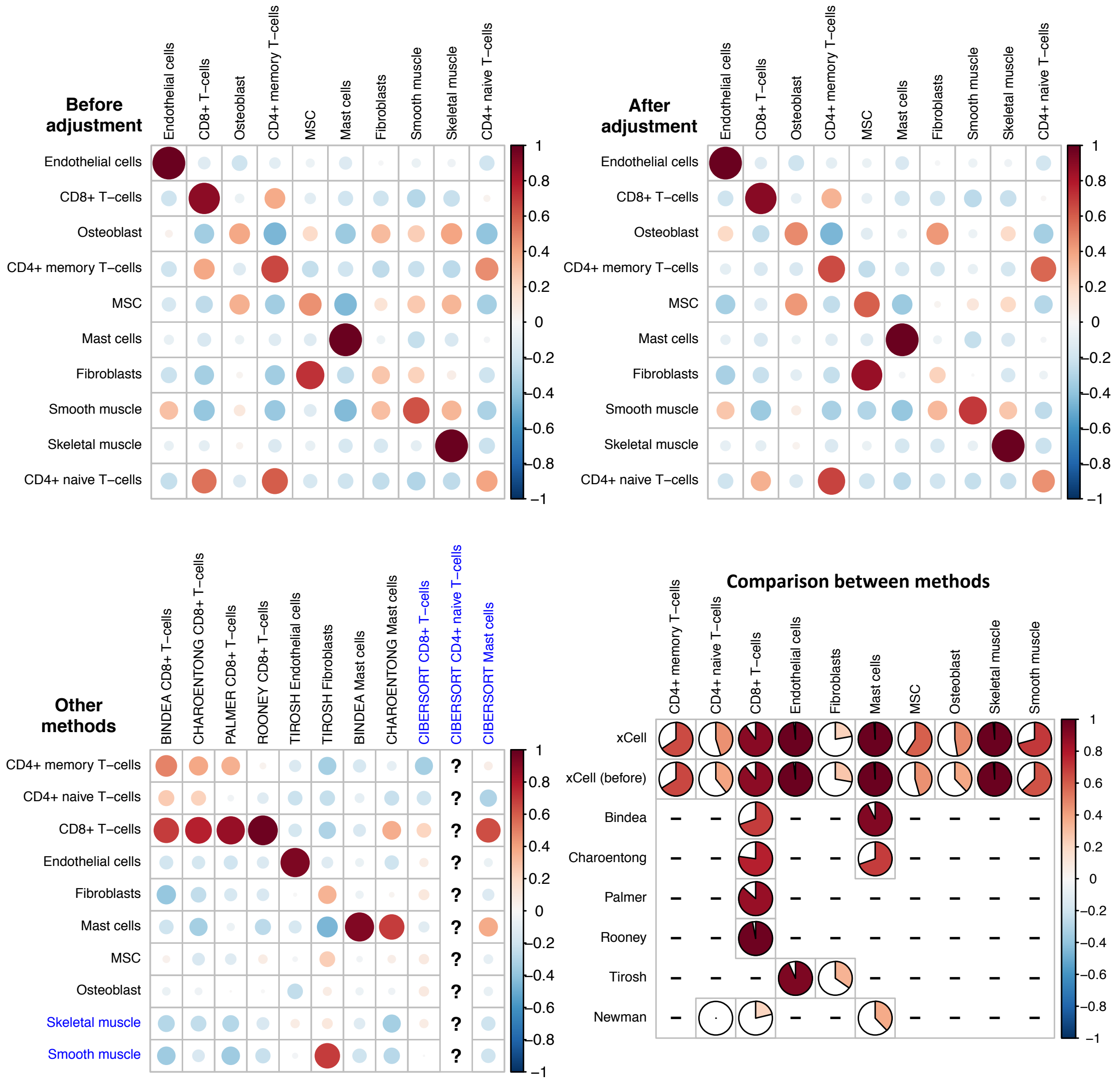

Summary table of simulation based on training samples

	Diagonal		Off-diagonal		
	Average R (diagonal)	Adjusted diagonal/ Non-adjusted	Adjusted diagonal/ Non-adjusted	Number of associations > 0.25	Adjusted diagonal/ Non-adjusted in >0.25 associations
HPCA 1	0.849	1.015	0.921	5	0.997
HPCA 2	0.872	1.049	0.926	6	0.879
HPCA 3	0.904	1.023	0.822	3	0.686
Novershtern 1	0.721	1.006	0.929	10	0.980
Novershtern 2	0.614	0.944	0.926	10	0.900
Novershtern 3	0.795	1.085	0.851	11	0.782
IRIS 1	0.853	1.091	0.857	10	0.758
IRIS 2	0.827	1.059	0.902	10	0.818
IRIS 3	0.813	1.070	0.876	15	0.808
Blueprint 1	0.874	0.994	0.921	3	0.704
Blueprint 2	0.885	0.995	0.941	3	0.894
Blueprint 3	0.906	1.013	0.944	4	0.846
ENCODE 1	0.872	1.005	0.985	2	0.965
ENCODE 2	0.854	1.015	0.945	1	0.978
ENCODE 3	0.857	0.994	0.967	2	0.947
FANTOM5 1	0.913	1.031	0.928	5	0.854
FANTOM5 2	0.886	1.035	0.862	5	0.568
FANTOM5 3	0.696	1.047	0.881	14	0.932
Average	0.860	1.014	0.930		0.854

Figure S6. Cell types inferences in gene expression simulations using testing samples. Each slide presents the results of xCell, published signatures and CIBERSORT, in predicting the underlying abundances of 250 simulated mixtures generated using the left-out testing samples, with 20% noise. **Top left**: Pearson coefficients of xCell before applying the spillover compensation. **Top right**: Pearson coefficients of xCell after applying the spillover compensation. **Bottom left:** Pearson coefficients of published signatures, CIBERSORT. **Bottom right**: Comparison between all methods.

Supplementary Figure 7:
Distributions of cell types' scores from random mixtures

Sequencing-based distributions

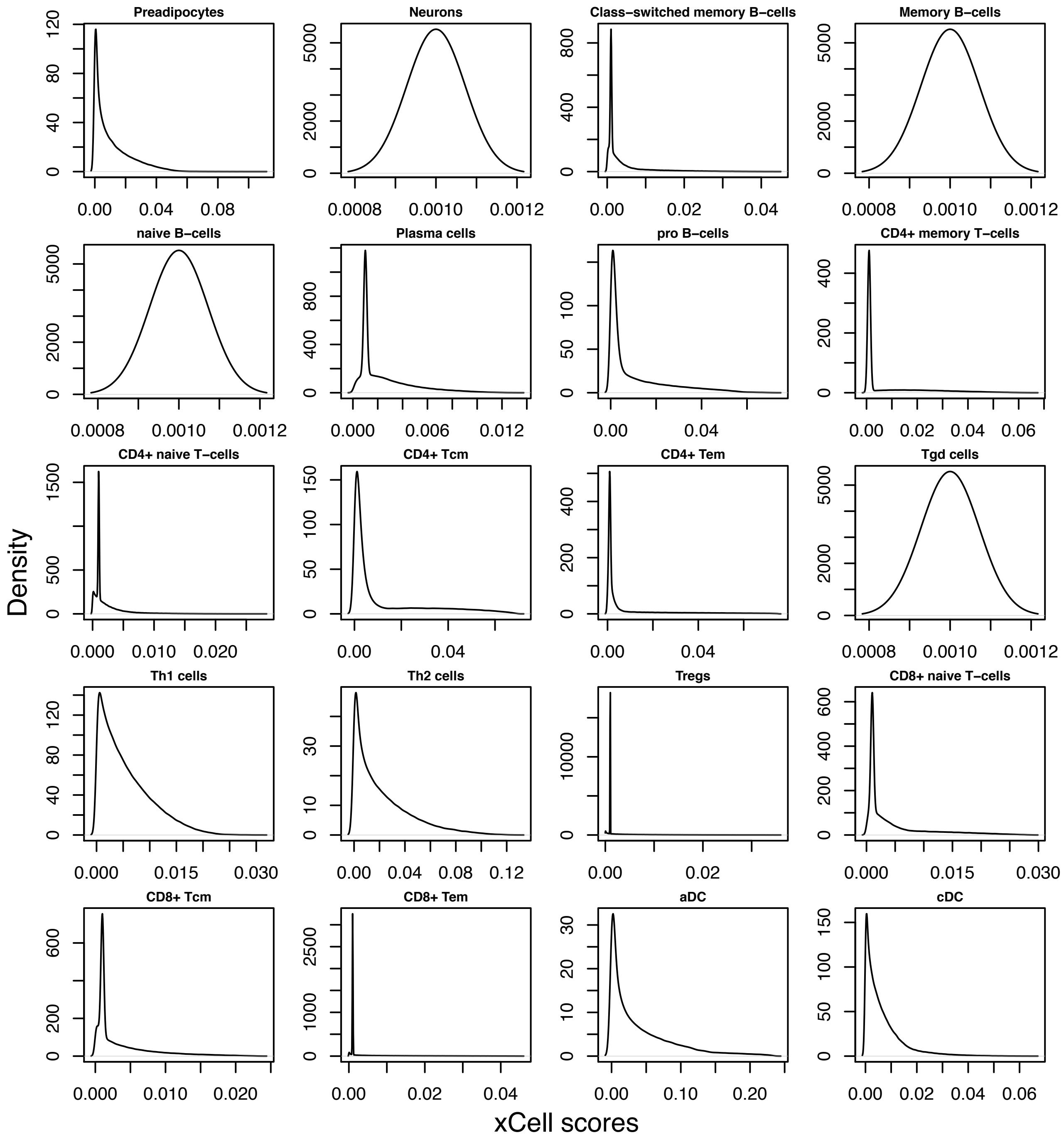

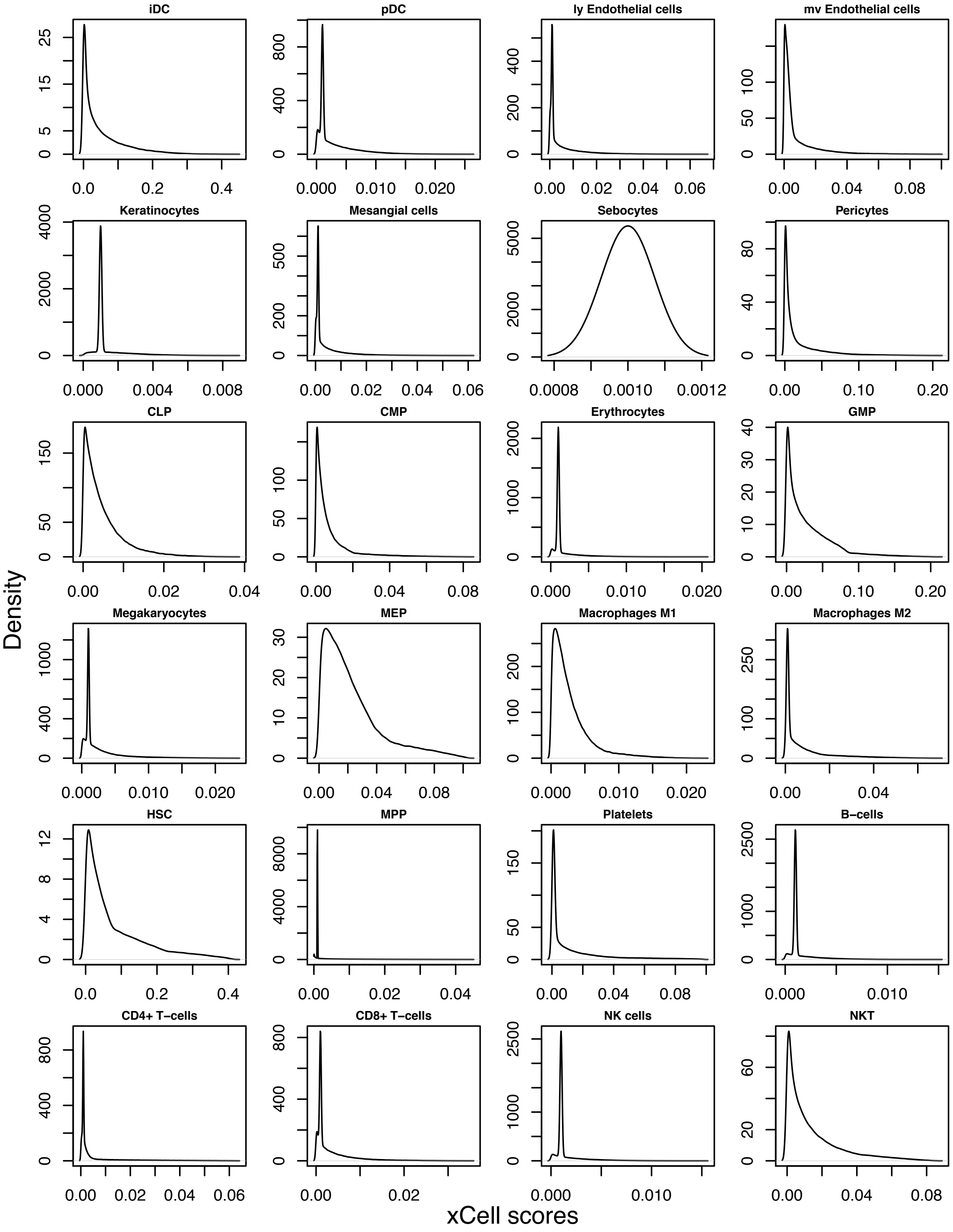
iDC
pDC
ly Endothelial cells
mv Endothelial cells
Keratinocytes
Mesangial cells
Sebocytes
Pericytes
CLP
CMP
Erythrocytes
GMP
Megakaryocytes
MEP
Macrophages M1
Macrophages M2
HSC
MPP
Platelets
B-cells
CD4+ T-cells
CD8+ T-cells
NK cells
NKT
Density
xCell scores

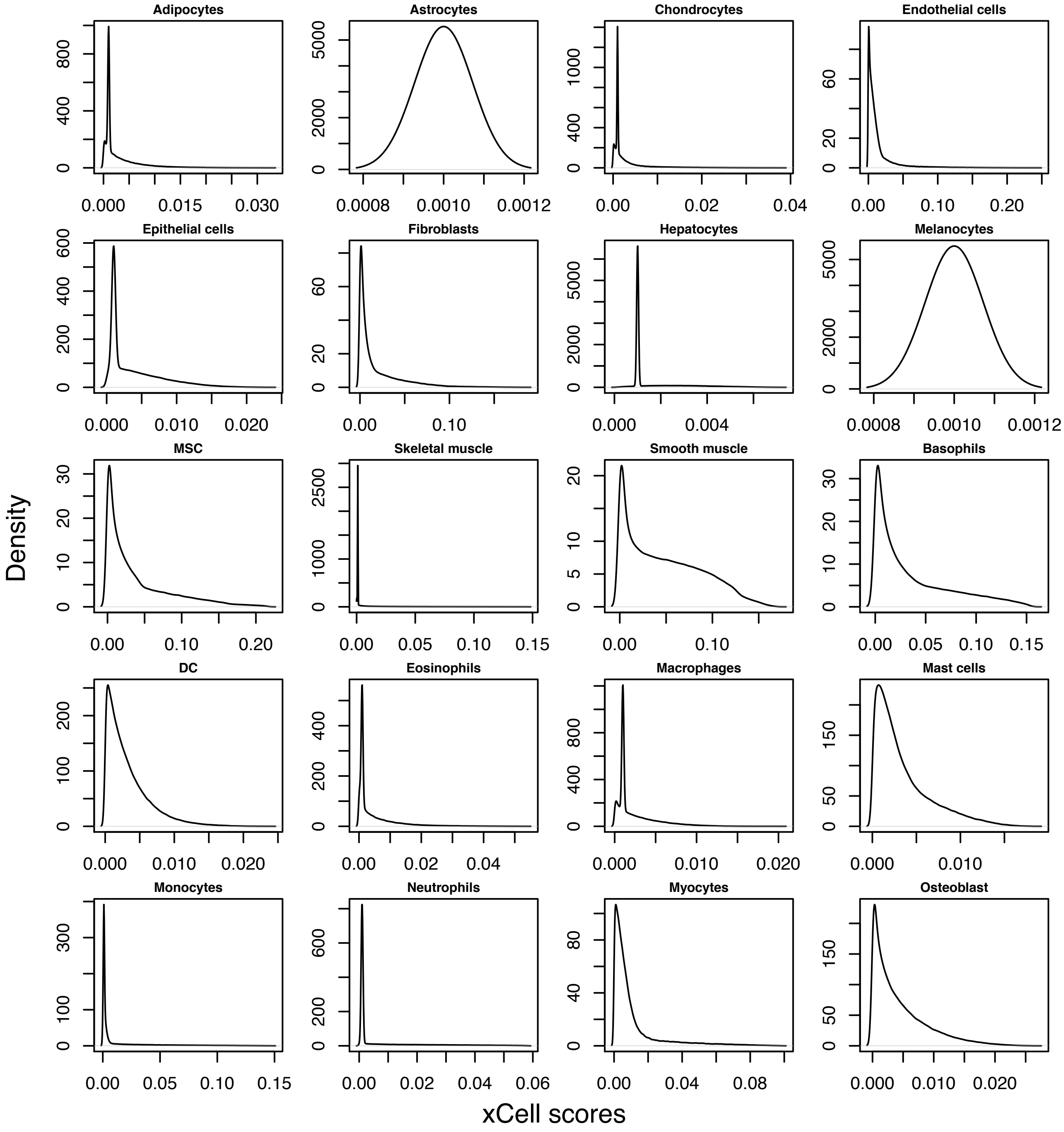

Adipocytes
Astrocytes
Chondrocytes
Endothelial cells
Epithelial cells
Fibroblasts
Hepatocytes
Melanocytes
MSC
Skeletal muscle
Smooth muscle
Basophils
DC
Eosinophils
Macrophages
Mast cells
Monocytes
Neutrophils
Myocytes
Osteoblast
Density
xCell scores

Array-based distributions

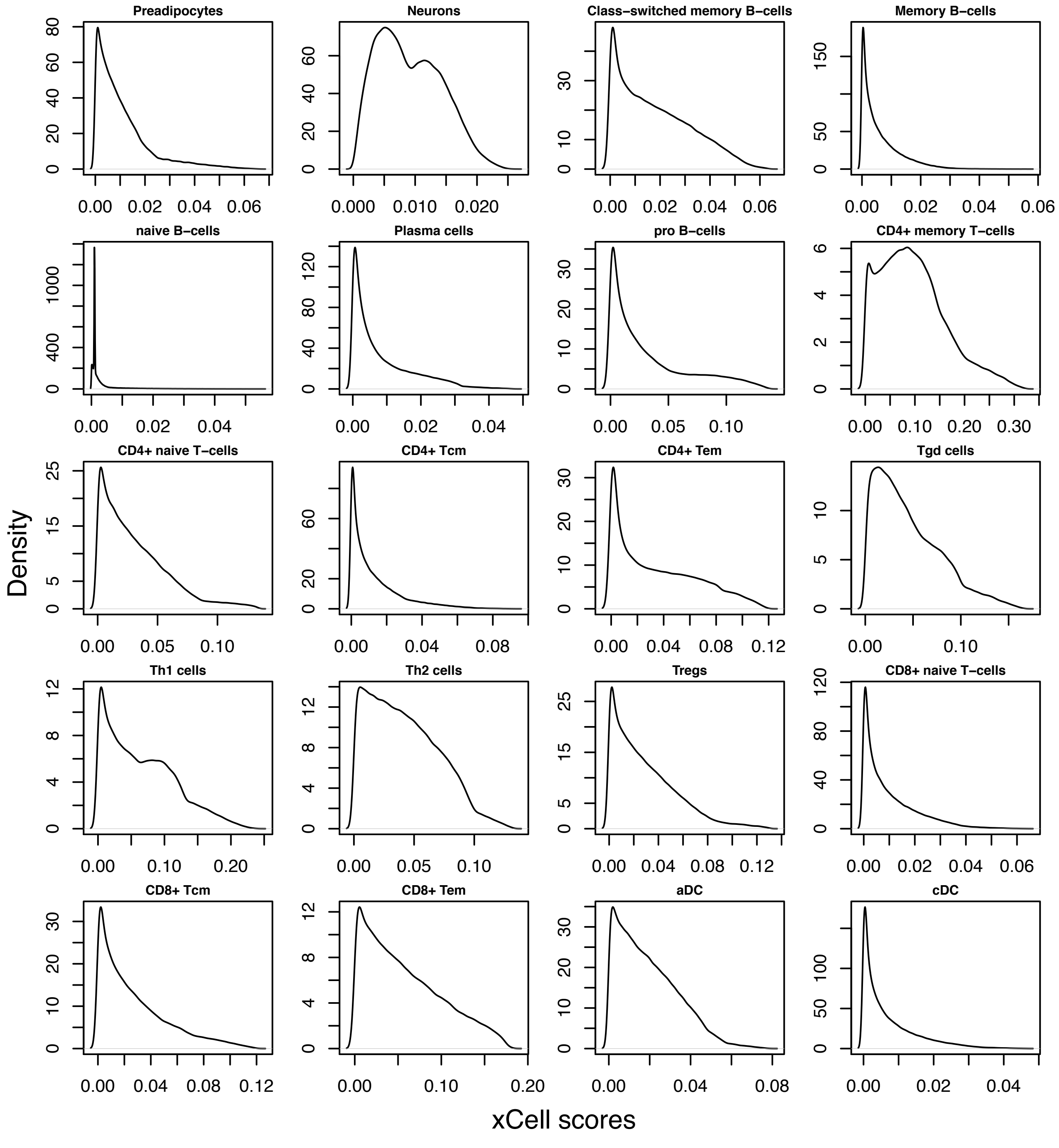

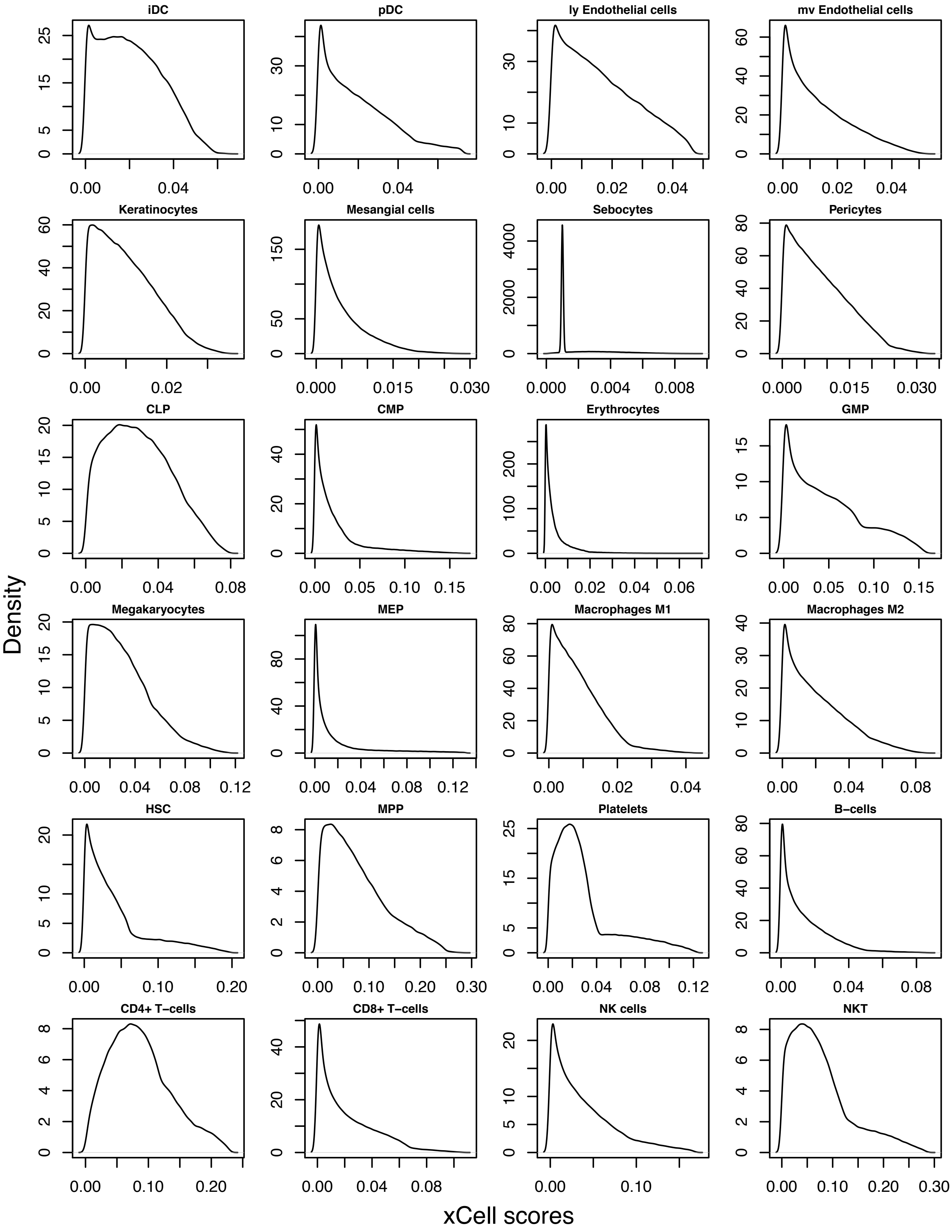

iDC
pDC
ly Endothelial cells
mv Endothelial cells
Keratinocytes
Mesangial cells
Sebocytes
Pericytes
CLP
CMP
Erythrocytes
GMP
Megakaryocytes
MEP
Macrophages M1
Macrophages M2
HSC
MPP
Platelets
B−cells
CD4+ T−cells
CD8+ T−cells
NK cells
NKT
Density
xCell scores

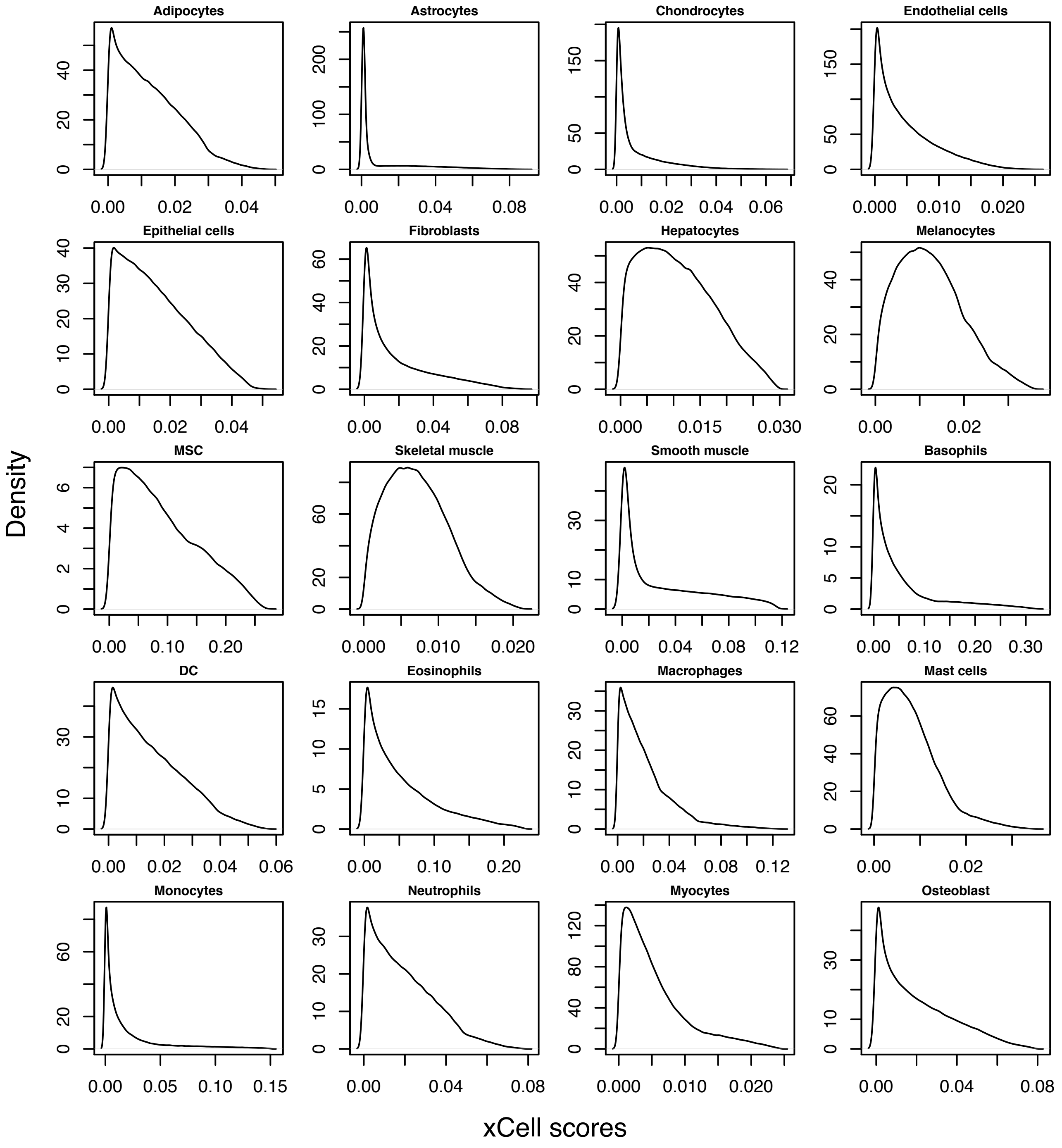

Figure S7. Distributions of cell types' scores from random mixtures. Beta distributions were learned from random mixtures excluding the cell type of interest in each of the 6 reference data sets (training samples only). The construction of the random mixture is described in the methods section. The distributions presented here, and are used for the statistical significance assessment, are combinations of the beta distributions – FANTOM5, Blueprint and ENCODE for sequencing-based inputs, and IRIS, Novershtern and HPCA for array-based inputs.

Supplementary Figure 8:
Dependencies between CD8+ T-cells and NK cells

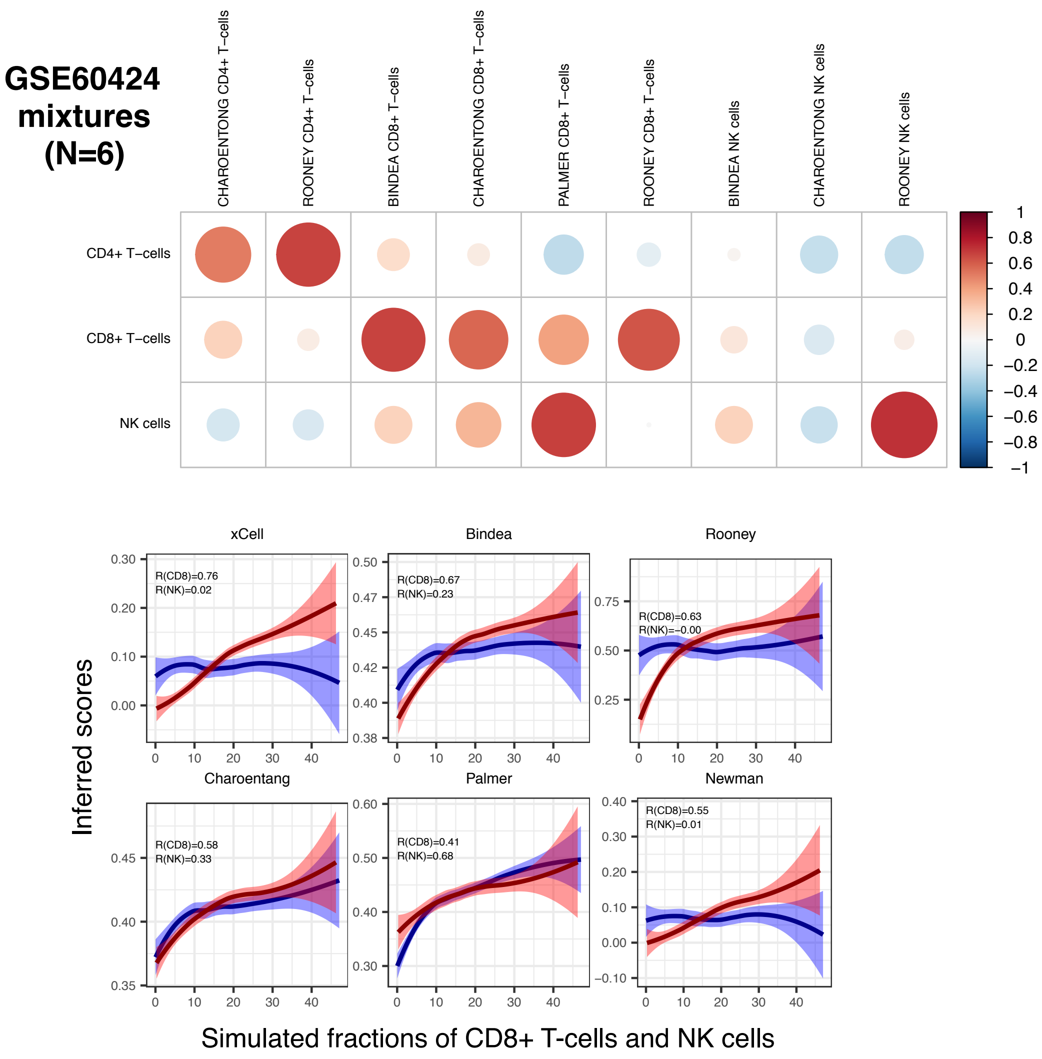

Figure S8. Dependencies between CD8+ T-cells and NK cells. Gene signatures tend to not be reliable in differentiating between closely related cell types. Here we show an example of a dependency between CD8+ T-cells and NK cells using simulated mixtures of GSE60424 RNA-seq expression profiles (which were not part of the generation of the method). The top plot shows the correlations of 9 published signatures with CD4+ T-cells, CD8+ T-cells and NK cells. Only the Rooney signatures was able to reliably infer all three cell types. The bottom plots show a curve fitted to the scores of CD8+ T-cells in each of the methods. The red curves are association with CD8+ T-cells underlying abundances, while the blue curves are with the NK underlying abundances. The blue line is expected to be flat, while the red curve is expected to be linear. Both our method and CIBERSORT (Newman) perform well here. The Rooney signatures is not linearly associated with the abundance.

Supplementary Figure 9:

CD8+ T-cells scores vs. CD8A expression in cancer cell lines

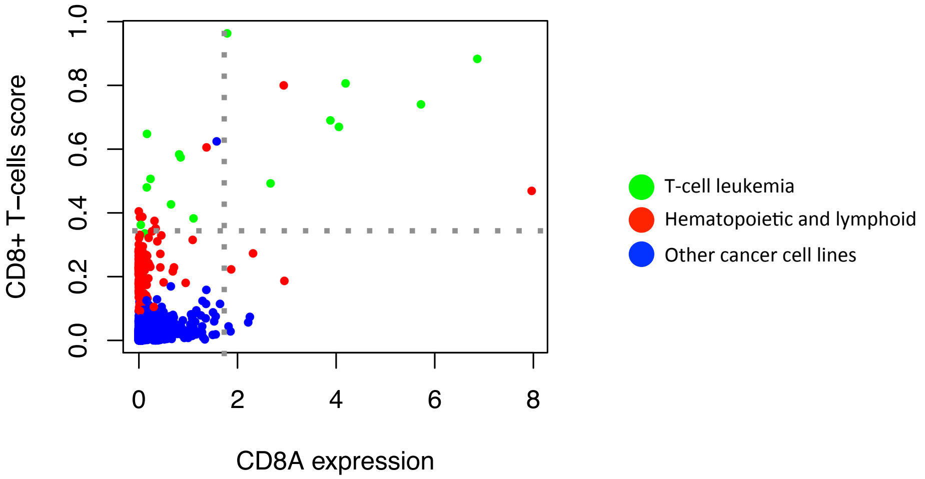

Cell ID	Primary	Subtype	CD8A expression	Rank	CD8+ T-cells scores	Rank
Pfeiffer	H&L	Diffuse Large B Cell Lymphoma	7.968	1	0.47	16
HPB-ALL	H&L	Acute Lymphoblastic T Cell Leukaemia	6.866	2	0.88	2
SUP-T1	H&L	Acute Lymphoblastic T Cell Leukaemia	5.723	3	0.74	5
KE-37	H&L	Acute Lymphoblastic T Cell Leukaemia	4.194	4	0.81	3
ALL-SIL	H&L	Acute Lymphoblastic T Cell Leukaemia	4.058	5	0.67	7
TALL-1	H&L	Acute Lymphoblastic T Cell Leukaemia	3.884	6	0.69	6
RI-1	H&L	B Cell Lymphoma Unspecified	2.954	7	0.19	125
CML-T1	H&L	Blast Phase Chronic Myeloid Leukaemia	2.939	8	0.80	4
PF-382	H&L	Acute Lymphoblastic T Cell Leukaemia	2.67	9	0.49	14
KCL-22	H&L	Blast Phase Chronic Myeloid Leukaemia	2.318	10	0.27	45
GSS	Stomach	Adenocarcinoma	2.251	11	0.07	196
NCI-H2227	Lung	Small Cell Carcinoma	2.217	12	0.06	232
KASUMI-1	H&L	Acute Myeloid Leukaemia	1.873	13	0.22	97
HUH-6-clone5	Liver	Hepatoblastoma	1.859	14	0.03	419
HARA	Lung	Squamous Cell Carcinoma	1.819	15	0.04	296
MOLT-16	H&L	Acute Lymphoblastic T Cell Leukaemia	1.791	16	0.96	1
DU-4475	Breast	Ductal Carcinoma	1.645	17	0.11	162
MOLT-3	Na	Na	1.579	18	0.62	9
DMS-79	Lung	Small Cell Carcinoma	1.56	19	0.02	567
NCI-H522	Lung	Non Small Cell Carcinoma	1.559	20	0.08	190
NCI-H1623	Lung	Adenocarcinoma	1.53	21	0.06	222
CHP-126	Autonomic Ganglia	Ns	1.504	22	0.09	179
MFE-280	Endometrium	Adenocarcinoma	1.498	23	0.02	591
HuT 78	H&L	Mycosis Fungoides-Sezary Syndrome	1.372	24	0.61	10
NCI-H211	Lung	Small Cell Carcinoma	1.371	25	0.16	142

Figure S9. CD8+ T-cells scores vs. CD8A expression in cancer cell lines. Many methods rely solely on the expression of CD8A is a marker for CD8+ T-cells abundance. Here we exemplify the risk in doing so – using 929 CCLE RNA-seq expression profiles we calculated xCell scores and CD8A expression (top). We observed that CD8A is high in some non-T-cells originating tumors, and low in some T-cell leukemias. Looking at the top 25 cancer cell lines, only 7 of the 16 T-cell leukemia's are in the top of expression (bottom), while all of them are in top of xCell's CD8+ T-cells scores.

Supplementary Figure 10:
xCell scores in 37 TCGA & TARGET cancer types

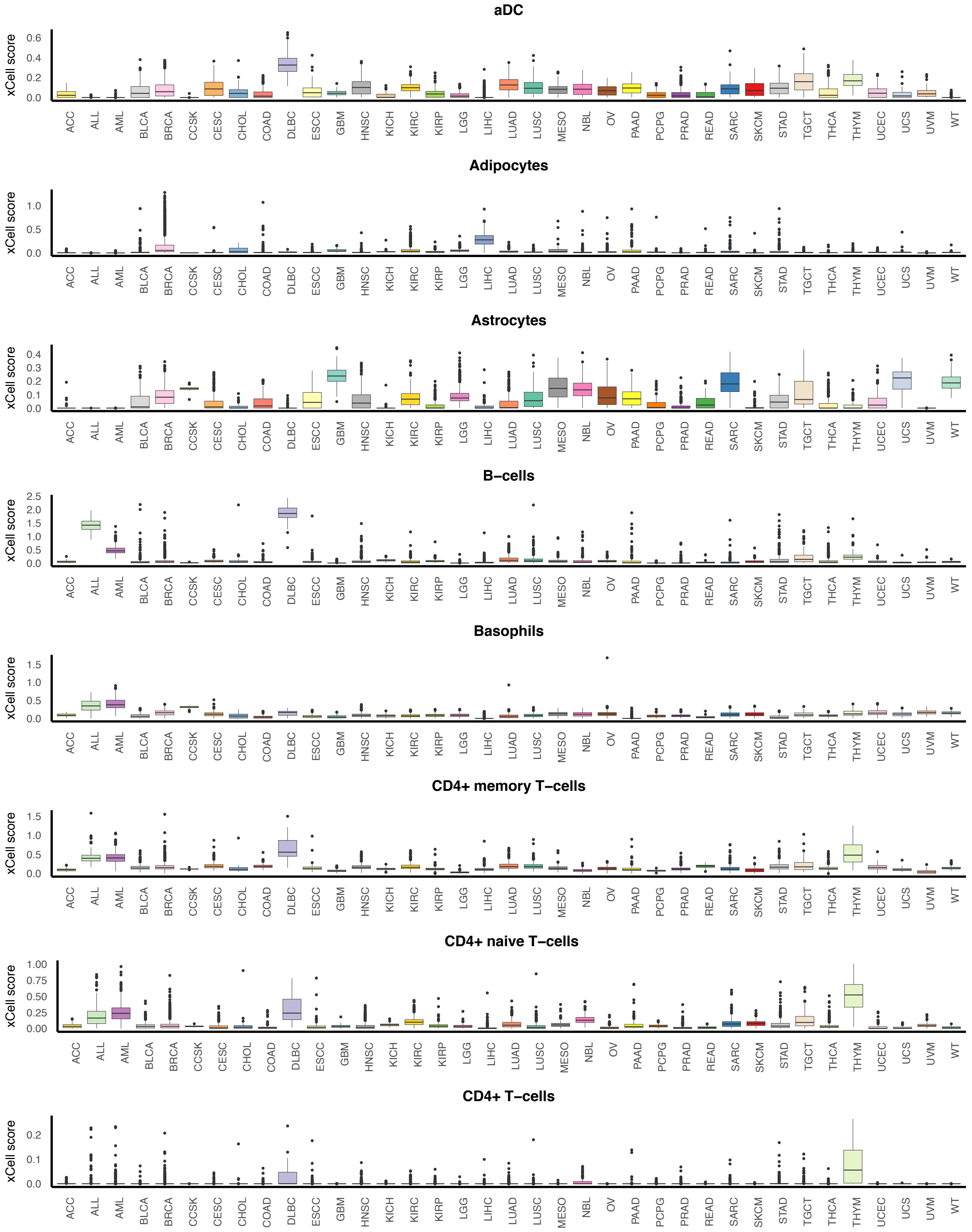

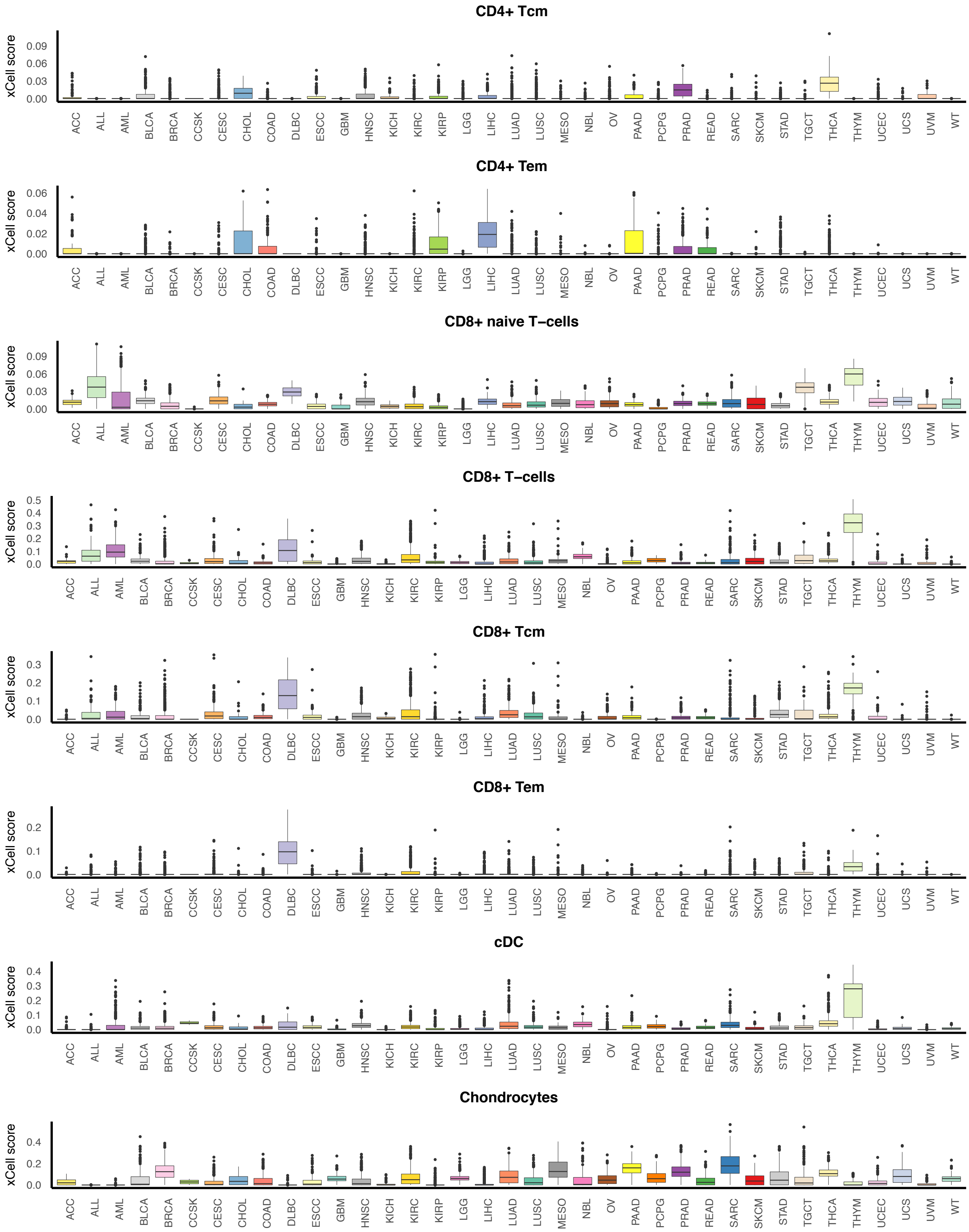

CD4+ Tcm
xCell score
0.00
0.03
0.06
0.09
CD4+ Tem
xCell score
0.00
0.02
0.04
0.06
CD8+ naive T−cells
xCell score
0.00
0.03
0.06
0.09
CD8+ T−cells
xCell score
0.0
0.1
0.2
0.3
0.4
0.5
CD8+ Tcm
xCell score
0.0
0.1
0.2
0.3
CD8+ Tem
xCell score
0.0
0.1
0.2
cDC
xCell score
0.0
0.1
0.2
0.3
0.4
Chondrocytes
xCell score
0.0
0.2
0.4
ACC ALL AML BLCA BRCA CCSK CESC CHOL COAD DLBC ESCC GBM HNSC KICH KIRC KIRP LGG LIHC LUAD LUSC MESO NBL OV PAAD PCPG PRAD READ SARC SKCM STAD TGCT THCA THYM UCEC UCS UVM WT

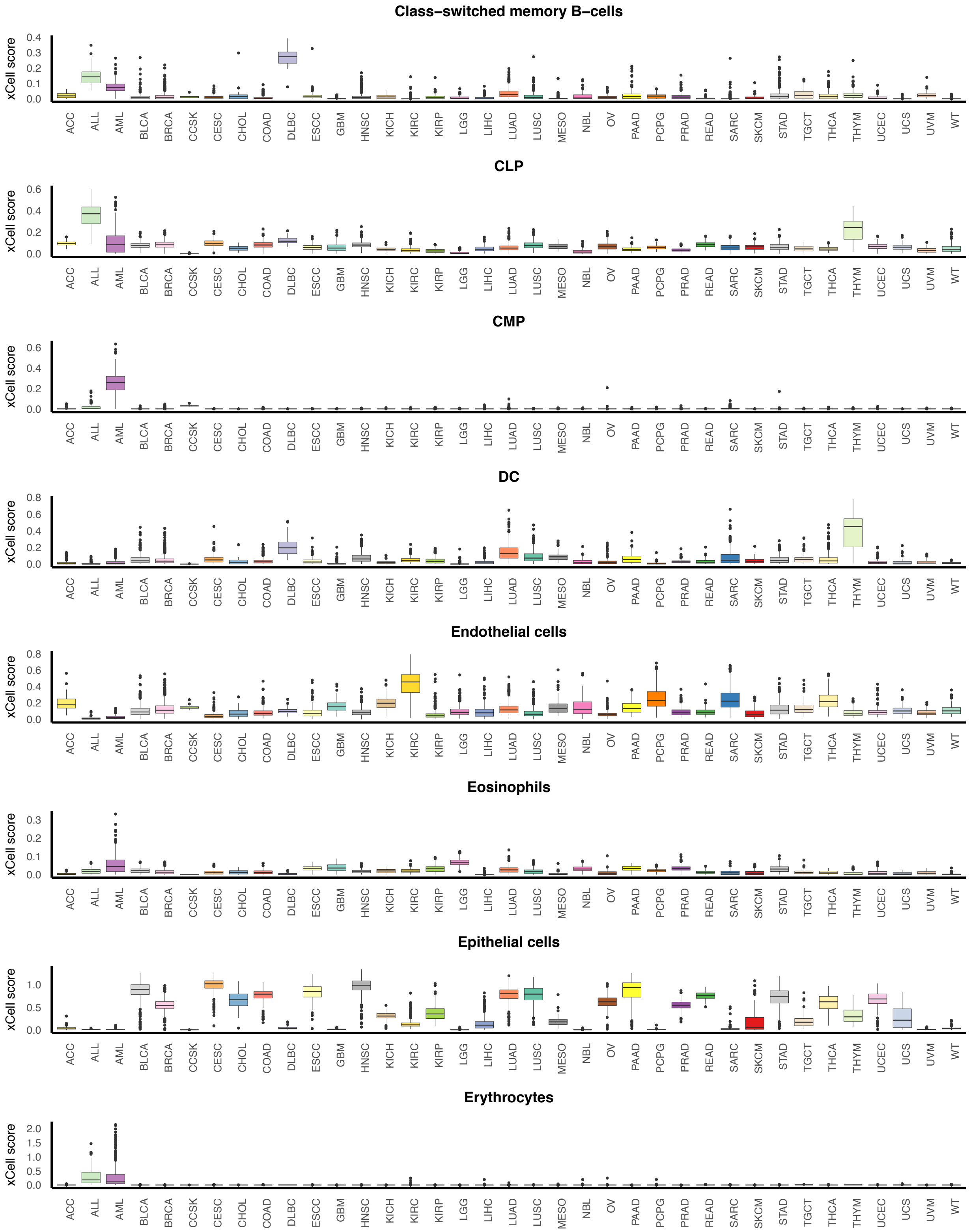
Class−switched memory B−cells
CLP
CMP
DC
Endothelial cells
Eosinophils
Epithelial cells
Erythrocytes
xCell score
ACC ALL AML BLCA BRCA CCSK CESC CHOL COAD DLBC ESCC GBM HNSC KICH KIRC KIRP LGG LIHC LUAD LUSC MESO NBL OV PAAD PCPG PRAD READ SARC SKCM STAD TGCT THCA THYM UCEC UCS UVM WT

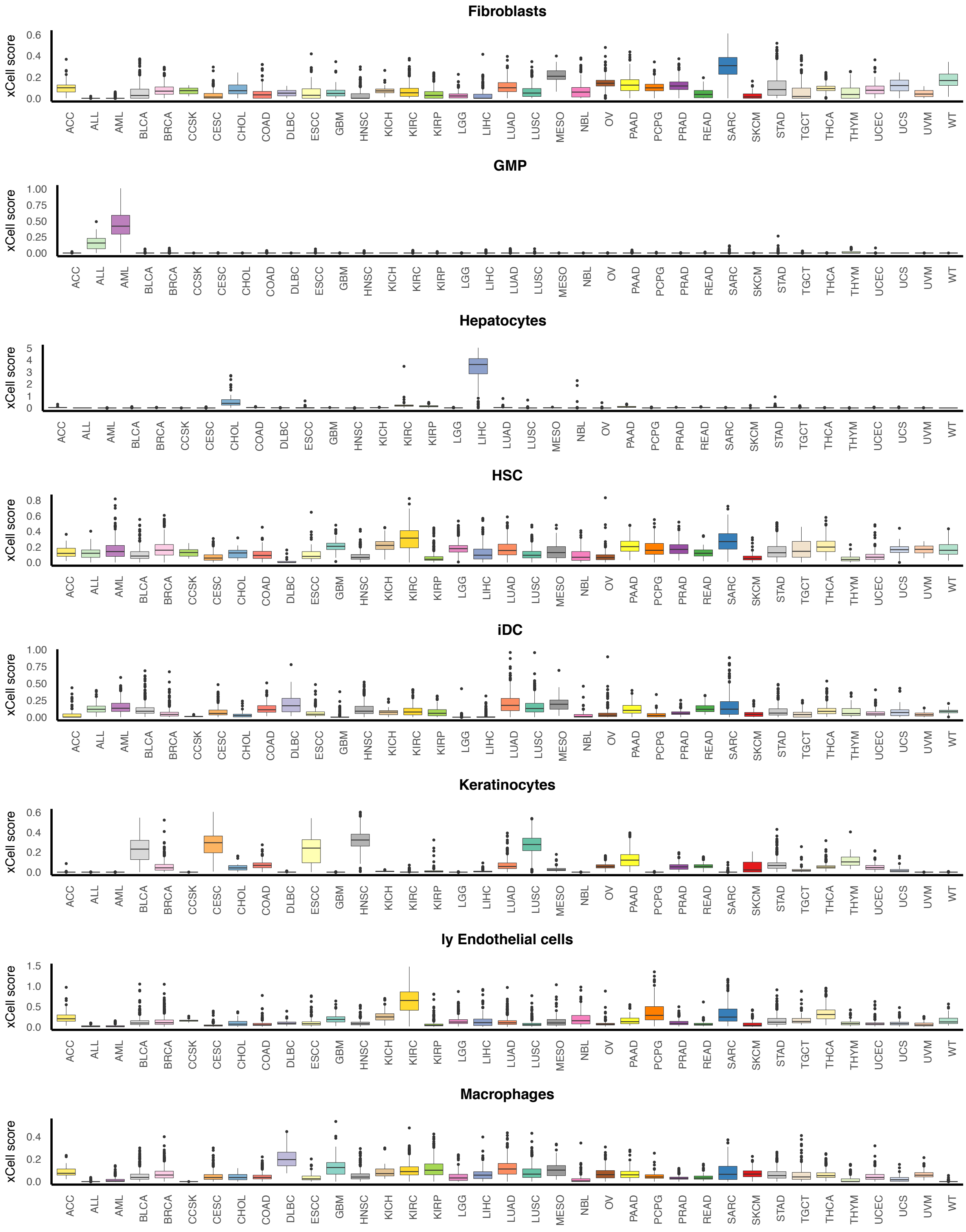
Fibroblasts
GMP
Hepatocytes
HSC
iDC
Keratinocytes
ly Endothelial cells
Macrophages
xCell score
ACC ALL AML BLCA BRCA CCSK CESC CHOL COAD DLBC ESCC GBM HNSC KICH KIRC KIRP LGG LIHC LUAD LUSC MESO NBL OV PAAD PCPG PRAD READ SARC SKCM STAD TGCT THCA THYM UCEC UCS UVM WT

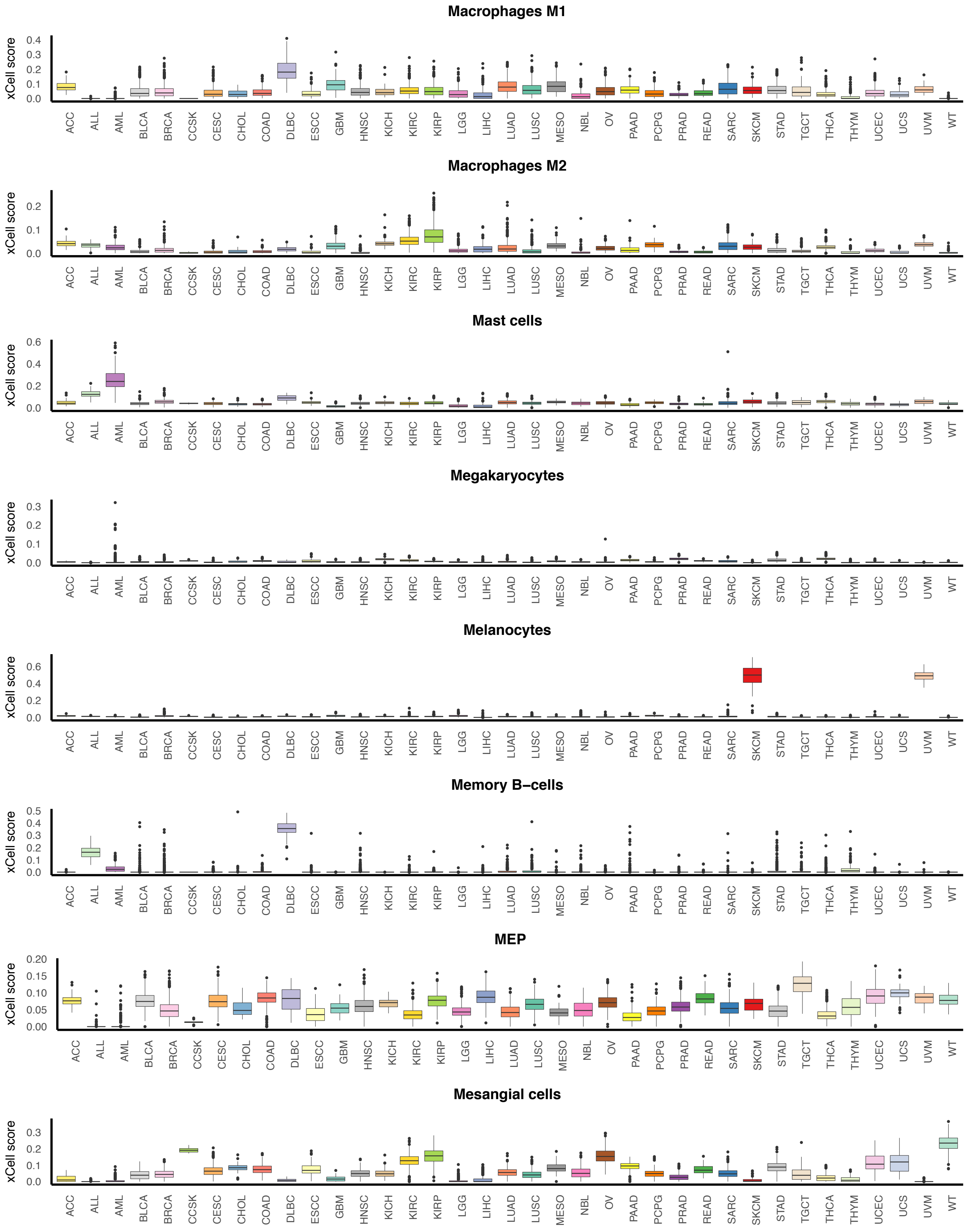

Macrophages M1
xCell score
0.4
0.3
0.2
0.1
0.0
ACC ALL AML BLCA BRCA CCSK CESC CHOL COAD DLBC ESCC GBM HNSC KICH KIRC KIRP LGG LIHC LUAD LUSC MESO NBL OV PAAD PCPG PRAD READ SARC SKCM STAD TGCT THCA THYM UCEC UCS UVM WT
Macrophages M2
xCell score
0.2
0.1
0.0
ACC ALL AML BLCA BRCA CCSK CESC CHOL COAD DLBC ESCC GBM HNSC KICH KIRC KIRP LGG LIHC LUAD LUSC MESO NBL OV PAAD PCPG PRAD READ SARC SKCM STAD TGCT THCA THYM UCEC UCS UVM WT
Mast cells
xCell score
0.6
0.4
0.2
0.0
ACC ALL AML BLCA BRCA CCSK CESC CHOL COAD DLBC ESCC GBM HNSC KICH KIRC KIRP LGG LIHC LUAD LUSC MESO NBL OV PAAD PCPG PRAD READ SARC SKCM STAD TGCT THCA THYM UCEC UCS UVM WT
Megakaryocytes
xCell score
0.3
0.2
0.1
0.0
ACC ALL AML BLCA BRCA CCSK CESC CHOL COAD DLBC ESCC GBM HNSC KICH KIRC KIRP LGG LIHC LUAD LUSC MESO NBL OV PAAD PCPG PRAD READ SARC SKCM STAD TGCT THCA THYM UCEC UCS UVM WT
Melanocytes
xCell score
0.6
0.4
0.2
0.0
ACC ALL AML BLCA BRCA CCSK CESC CHOL COAD DLBC ESCC GBM HNSC KICH KIRC KIRP LGG LIHC LUAD LUSC MESO NBL OV PAAD PCPG PRAD READ SARC SKCM STAD TGCT THCA THYM UCEC UCS UVM WT
Memory B−cells
xCell score
0.5
0.4
0.3
0.2
0.1
0.0
ACC ALL AML BLCA BRCA CCSK CESC CHOL COAD DLBC ESCC GBM HNSC KICH KIRC KIRP LGG LIHC LUAD LUSC MESO NBL OV PAAD PCPG PRAD READ SARC SKCM STAD TGCT THCA THYM UCEC UCS UVM WT
MEP
xCell score
0.20
0.15
0.10
0.05
0.00
ACC ALL AML BLCA BRCA CCSK CESC CHOL COAD DLBC ESCC GBM HNSC KICH KIRC KIRP LGG LIHC LUAD LUSC MESO NBL OV PAAD PCPG PRAD READ SARC SKCM STAD TGCT THCA THYM UCEC UCS UVM WT
Mesangial cells
xCell score
0.3
0.2
0.1
0.0
ACC ALL AML BLCA BRCA CCSK CESC CHOL COAD DLBC ESCC GBM HNSC KICH KIRC KIRP LGG LIHC LUAD LUSC MESO NBL OV PAAD PCPG PRAD READ SARC SKCM STAD TGCT THCA THYM UCEC UCS UVM WT

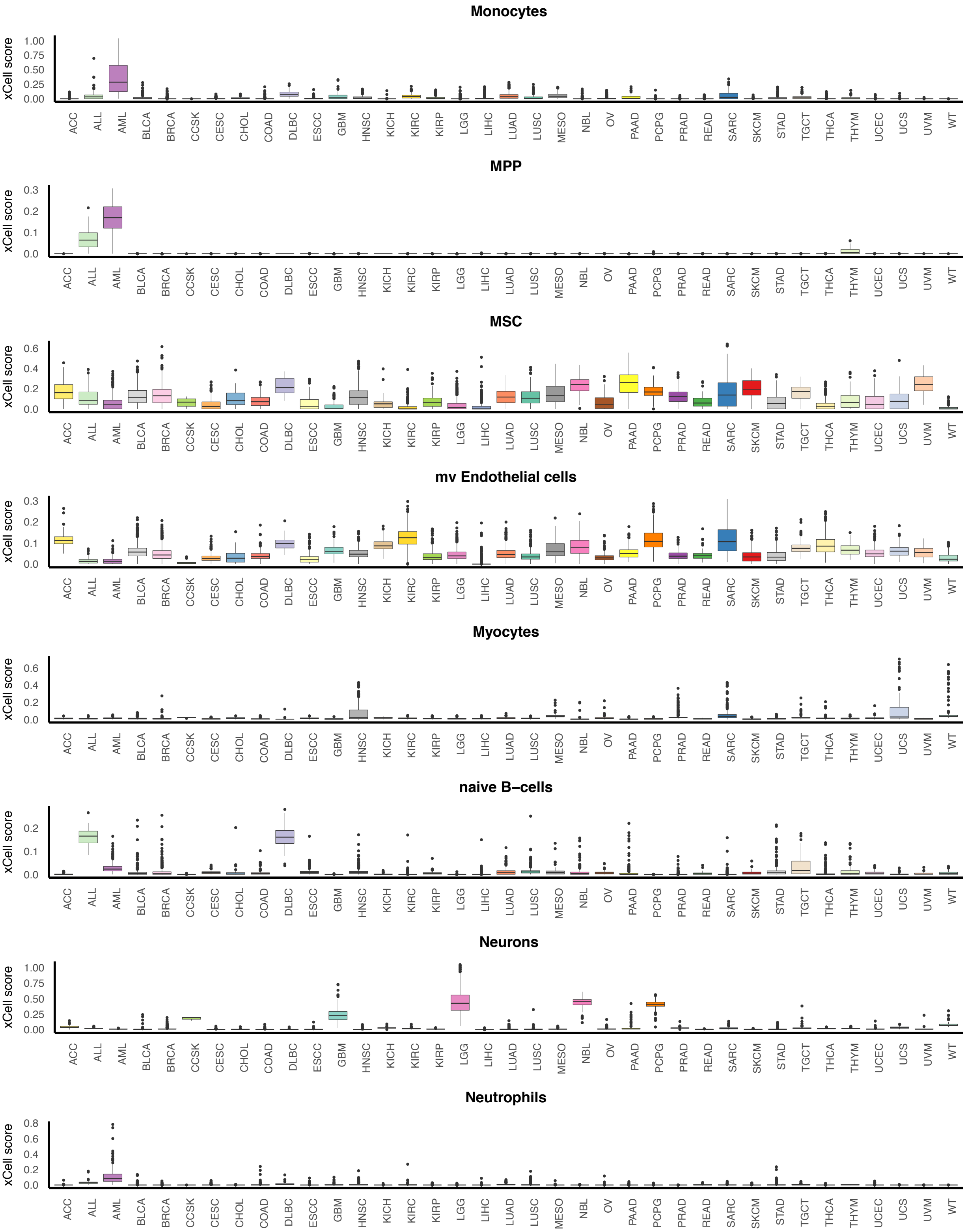

Monocytes
xCell score
1.00 0.75 0.50 0.25 0.00
MPP
0.3 0.2 0.1 0.0
MSC
0.6 0.4 0.2 0.0
mv Endothelial cells
0.3 0.2 0.1 0.0
Myocytes
0.6 0.4 0.2 0.0
naive B−cells
0.2 0.1 0.0
Neurons
1.00 0.75 0.50 0.25 0.00
Neutrophils
0.8 0.6 0.4 0.2 0.0
ACC ALL AML BLCA BRCA CCSK CESC CHOL COAD DLBC ESCC GBM HNSC KICH KIRC KIRP LGG LIHC LUAD LUSC MESO NBL OV PAAD PCPG PRAD READ SARC SKCM STAD TGCT THCA THYM UCEC UCS UVM WT

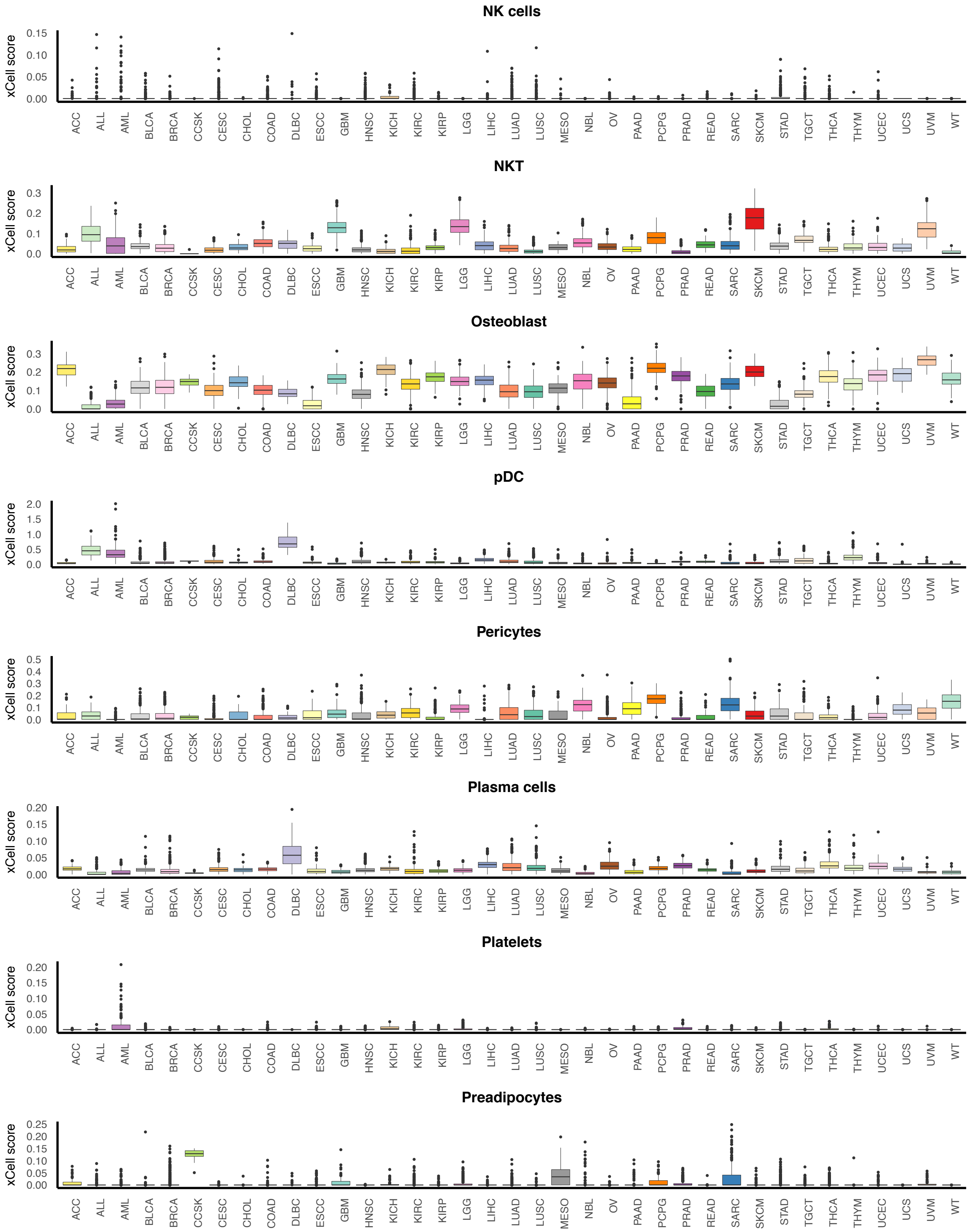
NK cells
NKT
Osteoblast
pDC
Pericytes
Plasma cells
Platelets
Preadipocytes
xCell score
ACC ALL AML BLCA BRCA CCSK CESC CHOL COAD DLBC ESCC GBM HNSC KICH KIRC KIRP LGG LIHC LUAD LUSC MESO NBL OV PAAD PCPG PRAD READ SARC SKCM STAD TGCT THCA THYM UCEC UCS UVM WT

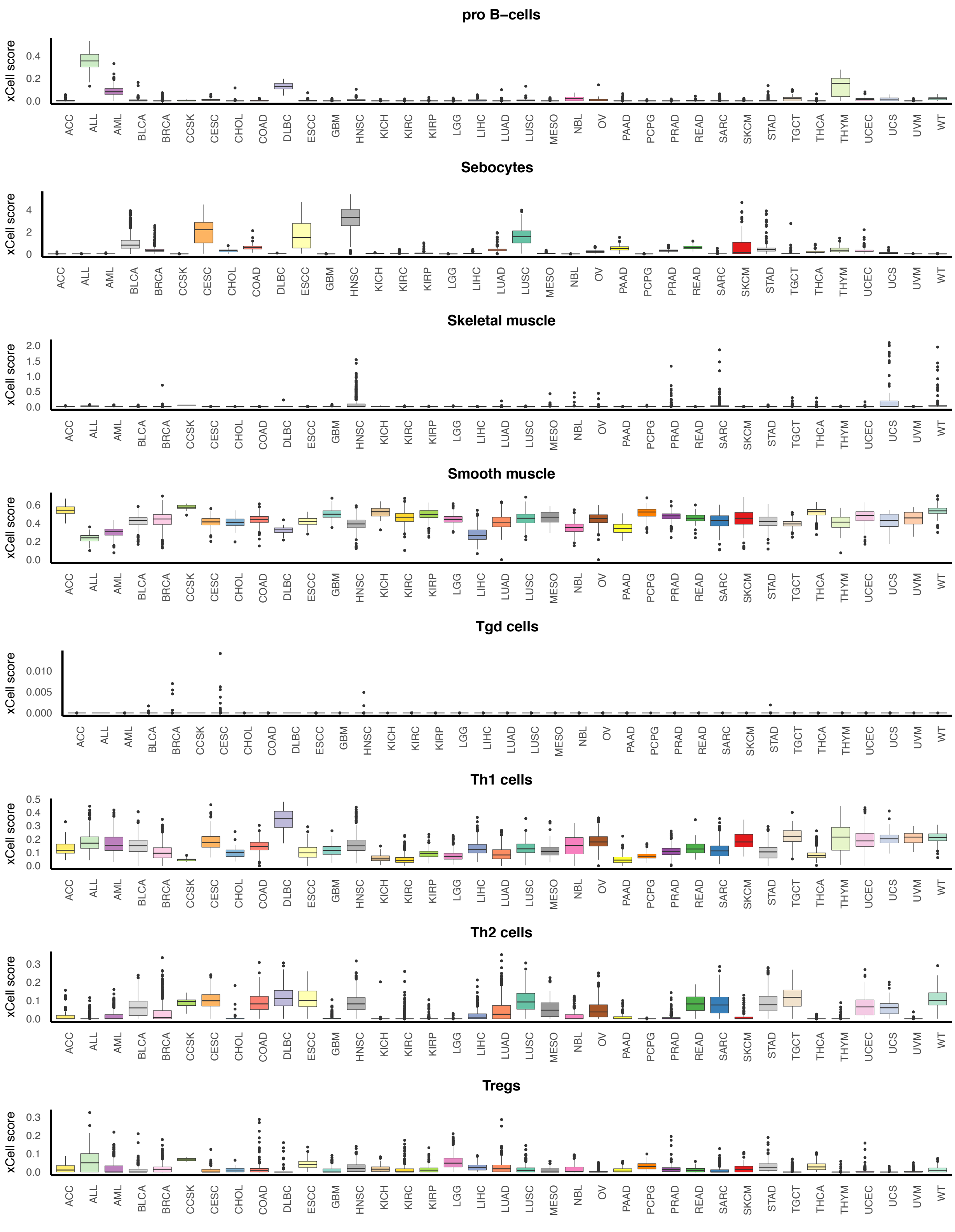

Figure S10. xCell scores in 37 TCGA & TARGET cancer types . Box plots of cell types enrichment scores in 9,947 TCGA & TARGET primary tumor samples across 37cancer types.

Supplementary Figure 11:
Purity estimations using xCell scores

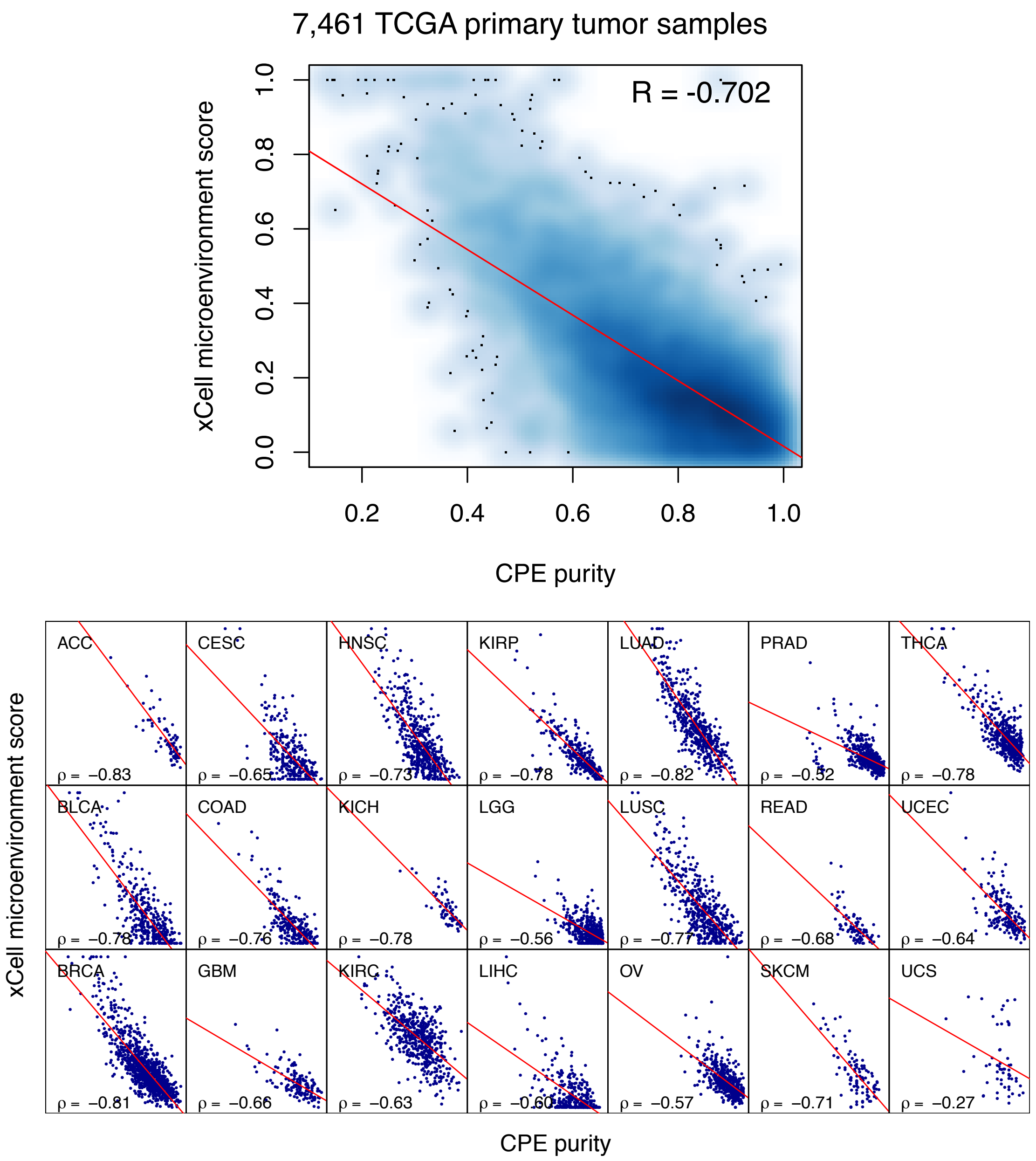

Figure S11. Purity estimations using xCell scores. We derived a microenvironment score as an. We correlated this score with the CPE purity measurements we previously generated using ESTIMATE, ABSOLUTE, LUMP and H&E slides. In all cancer types, with uterine carcinosarcoma as the exception we observed high correlations between our new microenvironment score and CPE.

Supplementary Figure 12:
t-SNE plots based on cell types scores

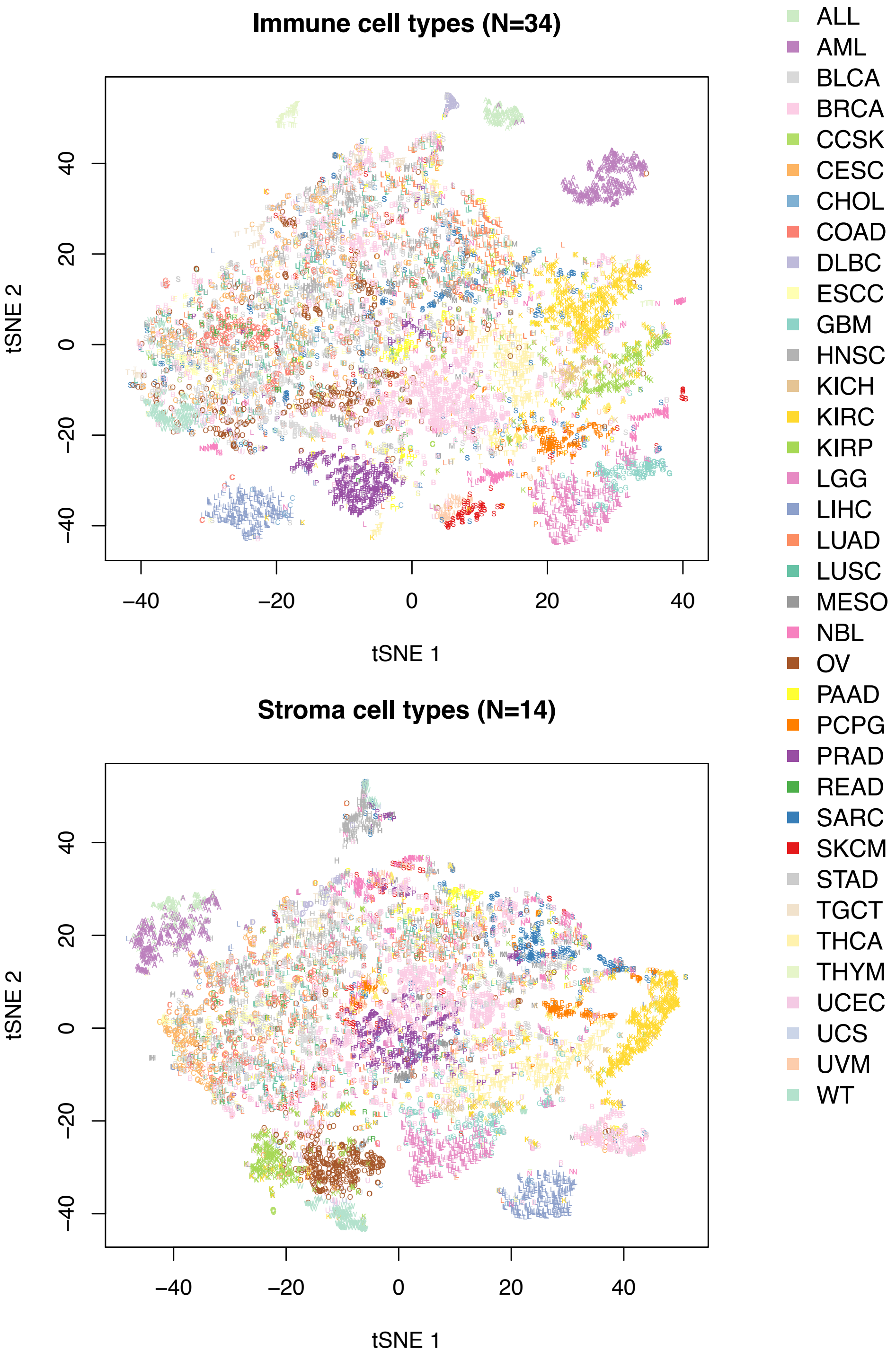

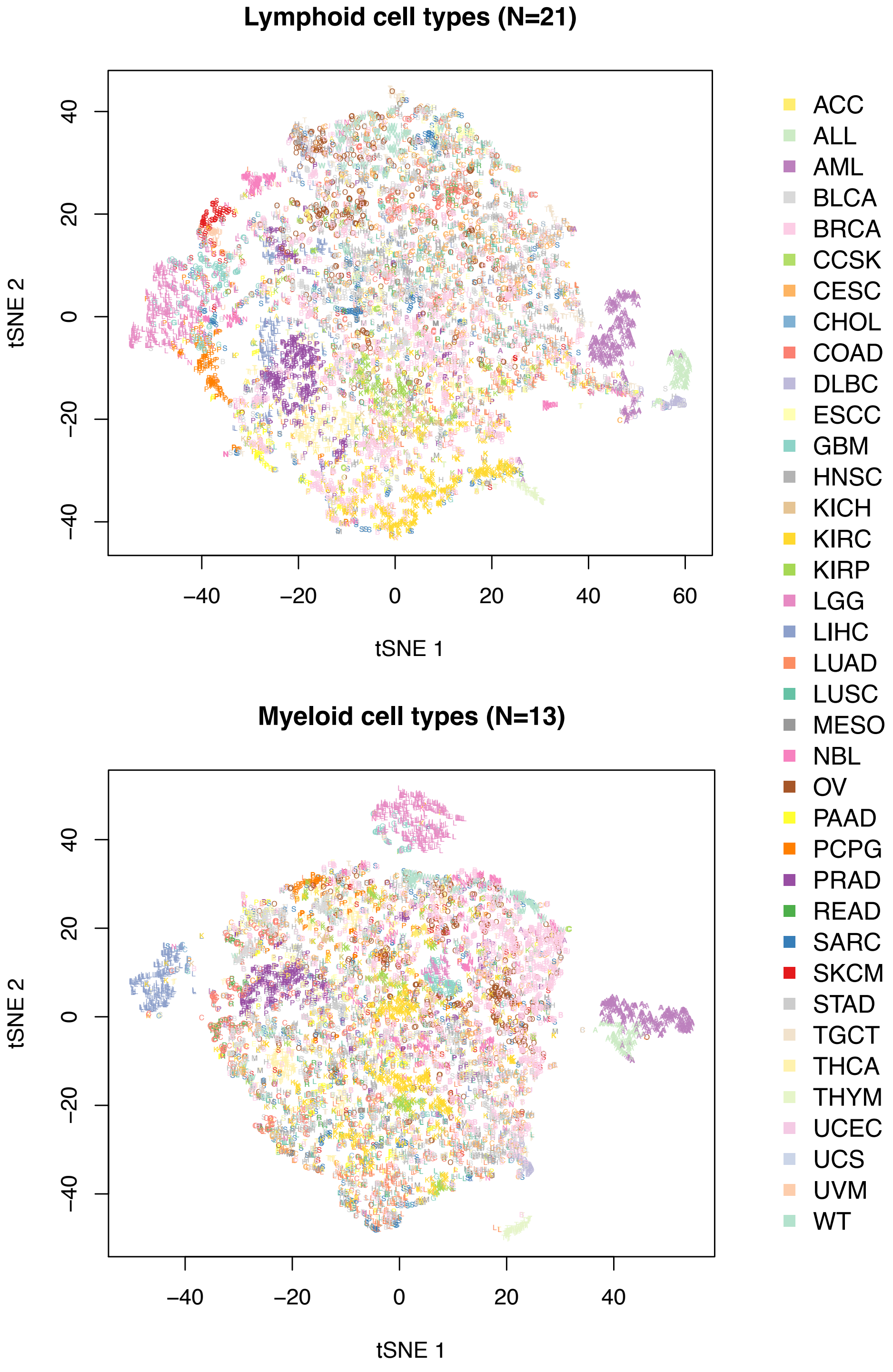

Lymphoid cell types (N=21)
tSNE 2
tSNE 1
40
20
0
−20
−40
−40
−20
0
20
40
60
Myeloid cell types (N=13)
tSNE 2
tSNE 1
40
20
0
−20
−40
−40
−20
0
20
40
ACC
ALL
AML
BLCA
BRCA
CCSK
CESC
CHOL
COAD
DLBC
ESCC
GBM
HNSC
KICH
KIRC
KIRP
LGG
LIHC
LUAD
LUSC
MESO
NBL
OV
PAAD
PCPG
PRAD
READ
SARC
SKCM
STAD
TGCT
THCA
THYM
UCEC
UCS
UVM
WT

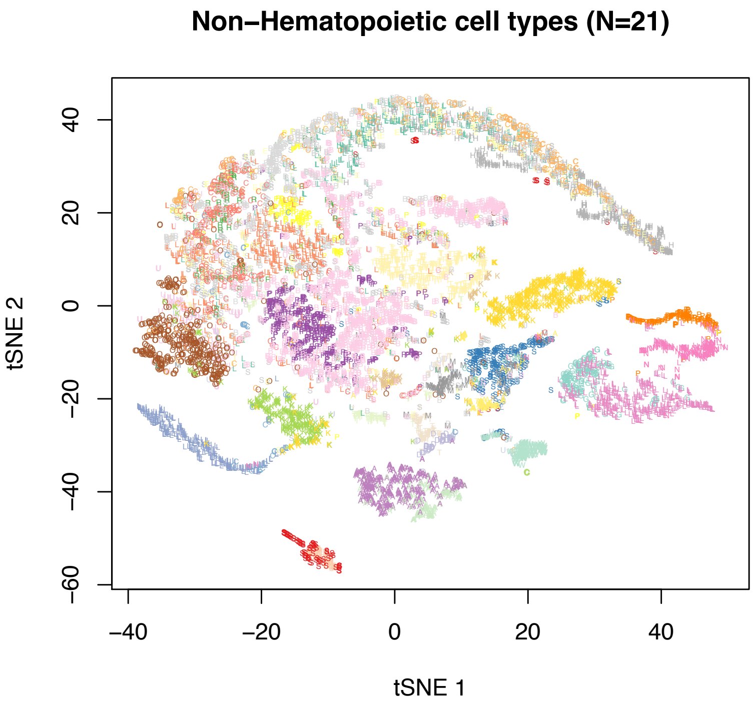

Figure S12. t-SNE plots based on cell types scores. Using the cell types inferences we generated t-SNE plots for 9,947 TCGA & TARGET primary tumor samples across 37 cancer types. In each plot the analyses was performed using a subset of the 64 cell types. The cell types included in each set can be found in supplementary table 1. Each subset of cell types distinguish different cancer types from each other.
